# Predictors of Occupational Burnout: A Systematic Review

**DOI:** 10.3390/ijerph18179188

**Published:** 2021-08-31

**Authors:** Yara Shoman, Emna El May, Sandy Carla Marca, Pascal Wild, Renzo Bianchi, Merete Drevvatne Bugge, Cigdem Caglayan, Dimitru Cheptea, Marco Gnesi, Lode Godderis, Sibel Kiran, Damien M. McElvenny, Zakia Mediouni, Ingrid Sivesind Mehlum, Dragan Mijakoski, Jordan Minov, Henk F. van der Molen, Evangelia Nena, Marina Otelea, Irina Guseva Canu

**Affiliations:** 1 Center of Primary Care and Public Health (Unisanté), University of Lausanne, 1066 Epalinges-Lausanne, Switzerland; e.elmay@gmail.com (E.E.M.); marca.sandy@gmail.com (S.C.M.); pascal.wild@unisante.ch (P.W.); zakia.mediouni@unisante.ch (Z.M.); irina.guseva-canu@unisante.ch (I.G.C.); 2 Institute of Work and Organizational Psychology, University of Neuchâtel, 2000 Neuchâtel, Switzerland; renzo.bianchi@unine.ch; 3 National Institute of Occupational Health (STAMI), 0363 Oslo, Norway; mdb@stami.no (M.D.B.); ingrid.s.mehlum@stami.no (I.S.M.); 4 Department of Public Health, Faculty of Medicine, Kocaeli University, İzmit 41001, Turkey; ccaglayan@kocaeli.edu.tr; 5 Faculty of Medicine and Pharmacy, Nicolae Testemitanu State University of Medicine and Pharmacy, 2004 Chisinau, Moldova; cheptea.dima@gmail.com; 6 Department of Public Health, Experimental and Forensic Medicine, University of Pavia, 27100 Pavia, Italy; marco.gnesi@unimib.it; 7 Department of Primary Care and Public Health, University of Leuven, 3000 Leuven, Belgium; lode.godderis@kuleuven.be; 8 Institute of Public Health, Hacettepe University, Ankara 06800, Turkey; sibelkiran@gmail.com; 9 Institute of Occupational Medicine, University of Manchester, Manchester M13 9WU, UK; Damien.McElvenny@iom-world.org; 10 Institute of Occupational Health of RNM, WHO Collaborating Center, 1000 Skopje, North Macedonia; dmijakoski@yahoo.com (D.M.); minovj@hotmail.com (J.M.); 11 Faculty of Medicine, Ss. Cyril and Methodius, University in Skopje, 1000 Skopje, North Macedonia; 12 Center for Occupational Diseases, Department of Public and Occupational Health, Amsterdam Public Health Research Institute, Amsterdam UMC, University of Amsterdam, Meibergdreef 9, 1105 AZ Amsterdam, The Netherlands; h.f.vandermolen@amsterdamumc.nl; 13 Medical School, Democritus University of Thrace, 68100 Alexandroupolis, Greece; enena@med.duth.gr; 14 Clinical Department 5, Carol Davila University of Medicine and Pharmacy, 020021 Bucharest, Romania; marina.otelea@umfcd.ro

**Keywords:** burnout, etiology, exhaustion, occupational health, prevention

## Abstract

We aimed to review occupational burnout predictors, considering their type, effect size and role (protective versus harmful), and the overall evidence of their importance. MEDLINE, PsycINFO, and Embase were searched from January 1990 to August 2018 for longitudinal studies examining any predictor of occupational burnout among workers. We arranged predictors in four families and 13 subfamilies of homogenous constructs. The plots of z-scores per predictor type enabled graphical discrimination of the effects. The vote-counting and binomial test enabled discrimination of the effect direction. The size of the effect was estimated using Cohen’s formula. The risk of bias and the overall evidence were assessed using the MEVORECH and GRADE methods, respectively. Eighty-five studies examining 261 predictors were included. We found a moderate quality of evidence for the harmful effects of the job demands subfamily (six predictors), and negative job attitudes, with effect sizes from small to medium. We also found a moderate quality of evidence for the protective effect of adaptive coping (small effect sizes) and leisure (small to medium effect sizes). Preventive interventions for occupational burnout might benefit from intervening on the established predictors regarding reducing job demands and negative job attitudes and promoting adaptive coping and leisure.

## 1. Introduction

The etiology of occupational burnout remains unclear, although it has elicited considerable interest in occupational health sciences over the last few decades [1,2,3,4]. Occupational burnout can have adverse consequences not only at an individual level (e.g., physical and mental health problems) [5] but also at an organizational level (e.g., absenteeism, poor performance at work, misjudgments and errors, job turnover) [6]. From both an individual and an organizational perspective, the prevention of occupational burnout has been viewed as the best approach to deal with this phenomenon [7].

Due to a lack of consensus on how occupational burnout should be defined and assessed, identifying the determinants of the syndrome has been challenging [8,9]. The European Network on the Coordination and Harmonization of European Occupational Cohorts (OMEGA-NET) recently proposed a harmonized definition of occupational burnout accepted by a majority of 50 experts from 29 countries [10], together with a systematic assessment of the psychometric quality of five occupational burnout measures [11]. Such work has helped to resolve semantic and methodological issues in assessing occupational burnout, particularly by focusing on exhaustion measurement. Nevertheless, the etiology of burnout still needs to be clarified by considering all predictors studied in longitudinal prospective studies. 

Prior systematic reviews of predictors of occupational burnout [12,13,14,15,16,17,18,19,20,21,22] had some restrictions, either because they focused on a specific occupational group (physicians, nurses, mental health professionals) [12,15,20] or studied only job-related predictors [23,24]; or selected studies with a particular duration of follow-up between two measurement points in longitudinal studies [13]. The duration of follow-up between two measurement points is particularly critical because the latency of occupational burnout onset remains uncertain [10,25,26,27]. Concerning the predictors of occupational burnout, several models have been commonly used in the literature. Along with the most prominent of these models, we found the job demand–control (JD-C) [28], the Demand–Control–Support (DCS) model [29], the Job Demands–Resources Model (JD-R) [30], and Effort–Reward-Imbalance (ERI) model [31]. Given the diversity of these models and uncertainty surrounding the predictors of occupational burnout, a systematic assessment including all longitudinally studied predictors, regardless of the underlying models, appeared essential, particularly for distinguishing between different types of predictors and assessing their respective effects. 

A reassessment of occupational burnout predictors is urgent for at least two main reasons. First, to resolve the between-study inconsistencies and conclude whether a given predictor has a protective or harmful effect on occupational burnout occurrence [32]. Secondly, it is important to know the level of evidence by a systematic analysis of all available findings, on all potential predictors, and in all occupations. We considered a quantitative synthesis for occupational burnout predictors focused on exhaustion the best approach following the OMEGA-NET harmonized definition of occupational burnout as a physical and emotional exhaustion state [10]. Additionally, exhaustion is the only characteristic of burnout that is recognized in all its conceptualization and operationalization [33,34,35]. It is also the only characteristic of burnout that is associated with decreases in objective job performance [36]. In such a context, unsurprisingly, many investigators have chosen to focus only on exhaustion when investigating burnout [12,37,38,39,40].

### Aims of the Current Study

This study aimed to review occupational burnout predictors, considering their type, effect size, and role (protective versus harmful), and the overall evidence of their importance.

## 2. Materials and Methods

We followed the Preferred Reporting Items for Systematic Reviews and Meta-Analyses (PRISMA) checklist [41] and the Synthesis Without Meta-analysis (SWiM) guidelines [42] for reporting this study.

### 2.1. Protocol and Registration

The protocol of this study is available on the international database PROSPERO with the registration number CRD42018105901 from: https://www.crd.york.ac.uk/PROSPERO/display_record.php?ID=CRD42018105901&ID=CRD42018105901 (accessed on 17 August 2018).

### 2.2. Inclusion and Exclusion Criteria

We performed systematic searches for studies examining the predictors of occupational burnout. We included original research studies that examined the effect of any predictors of occupational burnout measured as exhaustion, whatever the instrument used. The included studies were written in any European language, had a longitudinal design enabling exposure assessment before the burnout assessment, and were conducted among active workers (minimum 50 workers per group). The reasons for exclusion were: 1—no full text could be found; 2—studies that only reported an overall burnout score and/or measures other than exhaustion; 3—studies where participants were not professionally employed (e.g., students); 4—Studies where no measure of the variability of the study’s parameters and outcomes was reported (e.g., *p*-value or confidence intervals or the standard error of the mean).

### 2.3. Data Sources and Search Terms

The literature search was conducted over the period from 1 January 1980 to 8 August 2018 in three databases: MEDLINE, PsycINFO, and Embase via Ovid. We implemented the search strategy with the help of an experienced librarian; the full strategy can be found in Figure S1. We validated this literature search by achieving sufficient exhaustiveness of studies included in the latest systematic review on burnout, at the time of conducting the literature search, ref. [13] in working populations. In addition, we checked the reference lists from articles and reviews retrieved in our electronic search for any additional studies to include. In cases where we identified multiple publications describing a single study, we included the study only once, choosing one of the publications as the primary reference (the most complete one that included the latest follow-up) under which we listed all the others. We did not search the gray literature in order to avoid systemic bias and to guarantee the reproducibility and openness of our search and study selection strategy.

### 2.4. Data Collection and Analysis

#### 2.4.1. Study Selection 

We used the bibliography software EndNote X8 to import the collected studies. Then two independent reviewers screened the imported references. The reviewers removed remaining duplicates within each database, and between databases before they started the screening process. They used the above-mentioned inclusion and exclusion criteria to retain or reject articles and documented their decisions in a standardized form designed specifically for this study. The reference screening was performed in two steps: the title and abstract screening and full-text screening. In both steps of the screening, the references were equally distributed between 14 reviewers, while a second independent reviewer examined all of them independently. All discrepancies between the two reviewers’ assessments were discussed and solved by consensus, consulting a third reviewer when required.

#### 2.4.2. Data Extraction and Management 

We specially designed a standardized data extraction form in MS Excel, which we validated with a random sample of ten included studies. Five reviewers extracted the data independently, compared their data, discussed the discrepancies and flows, and improved the form until reaching an unambiguous valid format. The reviewers used this form for extracting data from studies assigned to them. The following data were extracted: study details (date of study, title, authors, and research question); methods (study design, primary outcome, predictor variables, exposures, potential confounders, and any other outcomes); participants population demographics (age, sex, socioeconomic background, and co-morbidities), inclusion and exclusion criteria and participation rate; outcomes (name and definition, how it was measured and reported), and statistics (beta coefficients from linear regressions, their standard errors (ideally), *p*-values or confidence intervals (CI), missing data and reasons for missing data). All extracted data were cross-checked by a second reviewer.

#### 2.4.3. Data Synthesis

First, we sorted and grouped all predictors into families corresponding to similar constructs or using similar measures. This enabled us to synthesize the abundant amount of information and make each family of predictors as homogeneous as possible. For example, based on a review on job burnout [43], we considered two main families of predictors: situational and individual. Job characteristics and organizational characteristics were included in the former, whereas personality characteristics and work attitudes were included in the latter. Non-occupational factors were grouped based on the type of predictor. Moreover, at the intersection between work and personal life, we considered a third family of predictors, the work-life interface [44,45], which refers to factors of personal life that overlap with work factors or vice versa. Finally, we classified other variables, either considered as predictors of occupational burnout not included in the other three main families or as intermediate outcomes or consequences of some working conditions, such as stress or satisfaction, in a fourth main family named “Perceived intermediate work consequences”. Secondly, we categorized predictors within each family into subfamilies in order that all predictors of one subfamily meet the conditions as follows: 1—related to the same or similar construct; 2—had the same theoretical valence/direction (e.g., two subfamilies “maladaptive coping style” and “adaptive coping style” instead of one subfamily “coping style”). 

#### 2.4.4. Statistical Analysis

**Figure 1:**
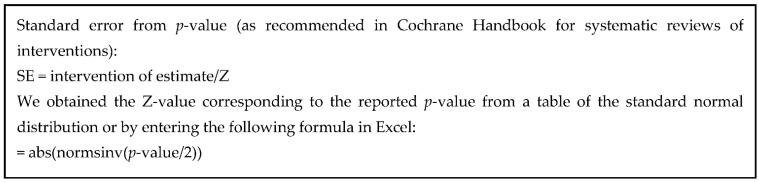
The formula for computing z-scores.

In this analysis, we only considered the direct path showing the effect of each predictor on the outcome. We also considered only the unadjusted effects whenever possible. By dividing the effect estimate (beta coefficient) by its standard error, we calculated the z-score for each study and each predictor. If the uncertainty parameter associated with the beta estimate was a *p*-value or confidence interval, we applied a formula (Figure 1) to convert them into standard errors. We plotted the z-scores per predictor type which enabled graphical discrimination of those associated with significantly increasing or decreasing occupational burnout rate. We further implemented the vote-counting method to identify the predominant direction of effect within a group of predictors [46]. In this analysis, the number of studies showing harmful effects was compared with the number of studies showing protective effects, regardless of the statistical significance [47]. The statistical significance of the predominant effect was then tested using the binomial test [46]. This method enabled us to test whether the subfamily effect was harmful (or protective) in less than 50% of studies. Finally, we computed effect sizes by extracting the correlation coefficients (for each exposure at time 1 correlating with the outcome at time 2), and then we used the formula suggested by Cohen [48]. An effect size less than or equal to 0.02, 0.15, 0.35 can be considered as “small”, “medium”, and “large”, respectively. We used R 3.6.2 statistical software (R Foundation for Statistical Computing, Vienna, Austria) for generating z-plots and STATA version 16.1 (StataCorp. LP, College Station, TX, USA) for all other analyses. 

#### 2.4.5. Risk of Bias Assessment

We assessed the risk of bias of each study included in the synthesis using the Methodological Evaluation of Observational Research Checklist (MEVORECH) [49]. This checklist provides separate examinations of external and internal validities with the labeling of major and minor flaws or poorly reported data on the study methodology. We performed the assessment using an MS Excel standardized form to report all elements of the MEVORECH, which we further analyzed using STATA. This allowed us to calculate an overall risk of bias score for each study and classify the studies into three categories, as follows: high risk of bias (i.e., the score > 43); moderate risk of bias (i.e., scores between 36 and 43), and low risk of bias (i.e., the score < 36). This step is necessary to evaluate the overall risk of bias in studies of the same predictor or (sub)family of predictors when assessing the overall quality of evidence.

#### 2.4.6. Quality of Evidence Assessment

We assessed the overall quality of evidence using the Grading of Recommendations Assessment, Development, and Evaluation (GRADE) approach [50]. The GRADE consists of five domains: risk of bias; inconsistency; indirectness; imprecision, and publication bias. The reviewers started with the assumption that the quality of evidence from the studies on a predictor or (sub)family of predictors was high, and then they downgraded the evidence in cases of high risk of bias, inconsistency, indirectness, imprecision, and publication bias. The resulting overall level of evidence per predictor or (sub)family of predictors was labeled as: high, moderate, low, or very low based on the total GRADE score.

## 3. Results

### 3.1. Selected Studies

**Figure 2:**
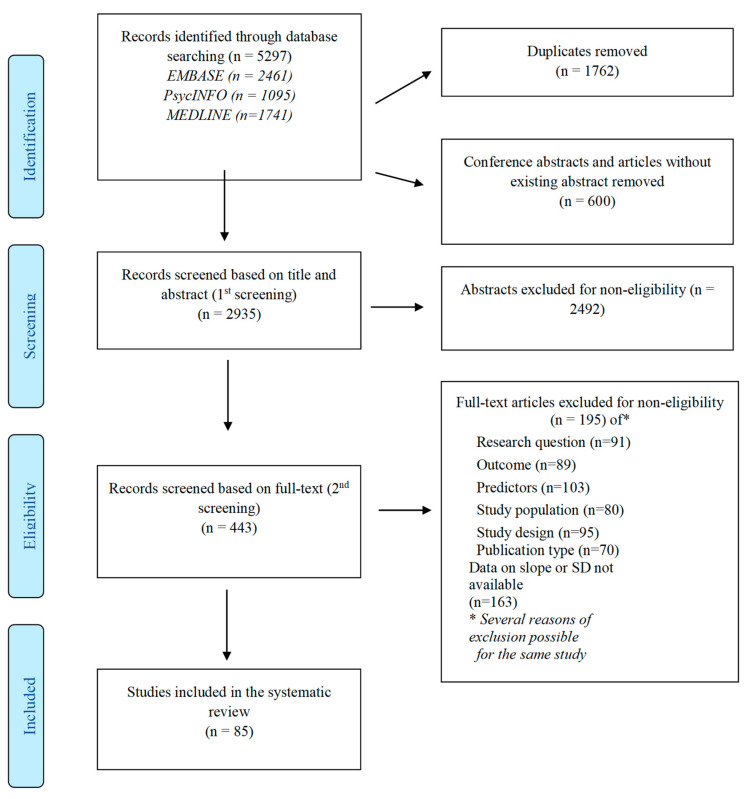
Flow-chart of the included studies.

Figure 2 Summarizes the study selection process. From 5297 identified references, 2935 were screened based on the title and abstract after duplicates, conference abstracts, and articles without abstracts had been removed. The rate of disagreement between reviewers regarding the eligibility of abstracts was less than 20%, and once solved, 443 references were retained for the full-text screening. In this step, the rate of disagreement regarding the eligibility of studies was less than 9%, and once solved, 85 articles were finally included in the review (Figure 2, and Table S1). 

### 3.2. Description of the Included Studies

The included studies were conducted between 1993 and 2018 (Table S1), mainly in European countries (Europe 71%, North America 23%, and Asia 6%). Teachers (15%), healthcare and social workers (13%), nurses (11%), physicians (6%), and police officers (5%) were the most studied occupations, though 9% of studies were based on the mixed sample of different occupations. Regarding the used time lags, 31% of the 85 studies used time lags (the time between the measurement points or so-called waves in the longitudinal study) less than one year, 44% used one-year time lag, and only 25% used more than one-year time lags. Regarding the hypothesis tested, 17 included studies tested the strain hypothesis for the JD-C, JD-R, and JDCS models. Four studies showed that their results were consistent with the JDCS model [12,51,52,53], whereas the results of two studies were in partial consistency (at least one dimension of the JDCS scale predicted exhaustion) [54,55]. Additionally, results from one study were not consistent with the JDCS strain hypothesis [56]. For the JD-C, we found four studies, with consistent [57], partially consistent [58], and not consistent results [59,60]. Among studies testing the JD-R strain hypothesis, four were in line with it [61,62,63,64], while three others were against [60,65,66].

We also found six studies which examined the buffer hypothesis, five of which were negative. These studies concluded that high job control or high job recourses do not alleviate the harmful effect of high job demands [53,56,60,65,66]. Only the results from the study of Feuerhahn et al. were in line with the buffer effect hypothesis [51]. Regarding the ERI model, the results from two studies were in line with this model [54,67].

### 3.3. Predictor (Sub)Families and Associated Z-Scores

**Figure 3:**
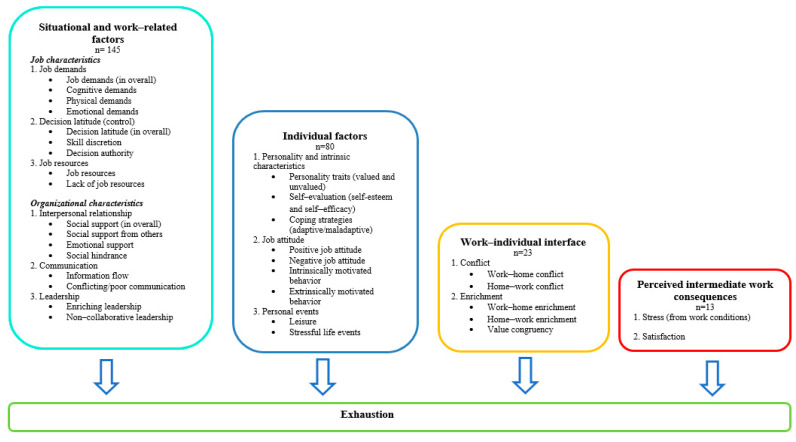
Description of predictor’s families and subfamilies.

**Figure 4:**
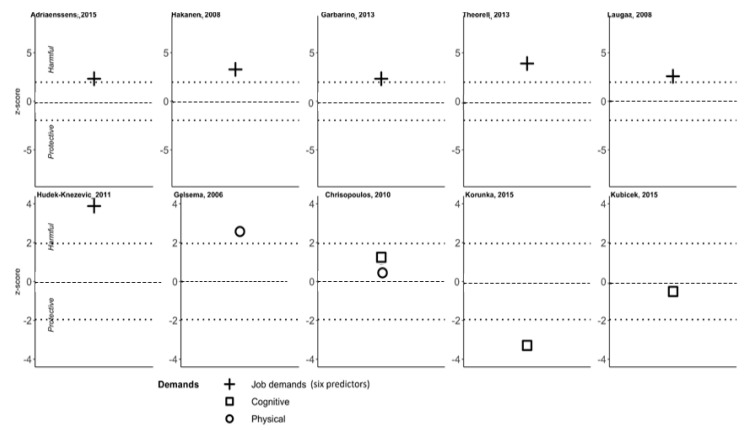
Z-scores for three types of Job demands (six predictors), Cognitive demands, and Physical demands subfamilies’ predictors.

In this review, we identified 261 predictors, which we grouped into four families and 13 subfamilies). Figure 3 depicts the content of each family of predictors, while Table S2 provides the definitions of predictors within each family and/or subfamily and their theoretical background. For each family and subfamily of predictors, we plotted z-scores calculated from studies investigating these predictors. Figure 4 shows that ten plots corresponding to 10 studies investigating at least one of the predictors belonging to the Job demands subfamily, Cognitive demands, and Physical demands subfamilies are presented together to facilitate the overall view of the z-scores distribution in this family of predictors. Z-score values higher than zero correspond to a positive association between the predictor and exhaustion, which is labeled as a harmful effect. Conversely, z-score values less than zero correspond to a negative association between the predictor and exhaustion, which is labeled as a protective effect. If the value of the predictor is outside the 95%CI (i.e., 1.96, −1.96; indicated by the dotted lines in Figure 4) then the effect is statistically significant. At zero, there is no association between the predictor and outcome (exhaustion). Figure 4 thus shows that within the Job demands subfamily, three studies [62,68,69] out of ten found a significantly harmful effect of high job demands overall with respect to exhaustion increase, and three studies [12,54,70] found this effect at borderline statistical significance. Gelsema et al. [52] found that physical job demand was harmful, while Korunka et al., found that Cognitive job demand was protective against exhaustion [71], and two other studies were inconclusive [61,63]. The complete set of plots for all (sub)families of predictors are available in Table S3. 

### 3.4. Results per (Sub)Family

#### 3.4.1. Job Demand, Decision Latitude (Job Control), and Job Resources

We found a moderate quality of evidence of harmful effects of small to moderate sizes for high Job demands (overall) based on six studies (Table 1). The quality of evidence for the high Quantitative demands (examined in 24 studies), harmful effects, and Job recourses (19 studies) protective effects was low, while the effects ranged between small and large sizes. The quality of evidence for the harmful effects of high Emotional demands (11 studies) was very low and the effect ranging between small and large sizes with a considerable variation across studies. For the Decision latitude (Job control) subfamily, we did not find any statistically significant effects (Table 1). 

#### 3.4.2. Interactions at Work, Communication, and Leadership

As shown in Table 1, the quality of evidence for high social support (21 studies) protective effects and high conflicting/poor communication (five studies) harmful effects was very low, with effect sizes ranging from small to medium but the majority of studies showed small sizes. We also found a very low quality of evidence for high social hindrance (11 studies) with harmful effects of sizes ranging from small to large, but the majority of studies showed small sizes. For the leadership subfamily, we did not find any statistical significance effects. 

#### 3.4.3. Personality Traits, Coping, Self-Evaluation, Job Attitudes, and Personal Events

The personality traits and self-evaluation subfamilies did not show any significant effects (Table 1). However, we found a moderate quality of evidence for high adaptive coping (six studies) protective effects of small effect sizes, high leisure such as relaxation, social activity, physical exercise, and relaxation (five studies) protective effects of sizes ranging from small to medium, and high negative job attitude (nine studies), harmful effects of sizes ranging from small to medium. The quality of evidence was very low for high positive job attitude (eight studies) protective effects of small sizes, and high self-esteem protective effects of sizes ranging between small to large.

#### 3.4.4. Work–Family Interface and Perceived Intermediate Work Consequences

In the Work–family interface family, there is only low quality of evidence for the work–family conflict (13 studies) harmful effect of sizes ranging from small to medium. We found a low quality of evidence for high stress from work conditions (ten studies) harmful effects of sizes ranging from small to large.

### 3.5. Results per Individual Predictor

Focusing on individual predictors (before grouping them into subfamilies), we found only six out of 261 predictors had a statistically significant effect of large size (Cohen’s f2 raging between 0.39 and 0.69) on occupational burnout rate (Table S1). Three of them had a low risk of bias, including effort–reward imbalance and work and time demands (having a harmful effect) and core self-evaluation, having a protective effect. The other three predictors were of a moderate risk of bias, with workload and class disruption having a harmful effect and increased emotional competencies having a protective effect.

## 4. Discussion

### 4.1. Main Findings

Performing this systematic review of 85 studies and 261 predictors led us to conclude that the evidence for any previously established risk or protective factor does not reach a high level. We found a moderate quality of evidence for only four subfamilies of predictors, namely the harmful effects of job demands (overall) and negative job attitudes, as well as for the protective effects of adaptive coping and leisure. Low quality of evidence was found for the harmful effects of quantitative demands, Work–family conflict, and stress from work conditions. 

The grouping of the predictors was performed depending on the theory or framework behind the predictors. However, for some predictors, namely “Satisfaction” and “Stress” from work conditions, we encountered some disagreements. Some authors considered them as situational predictors (related to work conditions), while for others they represented a consequence of work conditions and therefore an intermediate/moderate effect on the pathway between the exposures and occupational burnout. Nevertheless, it is noteworthy that these predictors were measured using different instruments than the ones applied for predictors in the “Situational factors” family. Accordingly, we decided to group them as an independent family entitled “Perceived intermediate work consequences”. 

The Job Demand Control model (JD-C model) is among the most studied models for occupational burnout [72] and our results indicated a moderate quality of evidence for job demands as a harmful effect of large size. Otto et al. [73] suggested increasing the job control of employees and reducing job demands. Nevertheless, Konze et al. raised the question that job control could be a double-edged sword [58], and by taking a closer look at skill discretion and autonomy, we observed that for these two predictors the direction of effects varied across studies, with small effect sizes, no significant results, and with a very low quality of evidence and no significant results. Apparently, these predictors require further investigation with representative samples and multiple wave studies to assess their effects on occupational burnout. Increasing job resources can serve as a protective factor [73], as shown by this review. Social support had a protective effect also supported by work-related stress literature [74]. Social hindrance had a harmful effect in line with the finding of Schilpzand et al., which suggested that hindrance affects the employees’ well-being [75]. There is an assumption that communication (i.e., the quality and effectiveness of communications between workers) can be an important predictor of occupational burnout [76], specifically communication climate and communication satisfaction, and the results of this review showed that conflicting/poor communication has an important harmful effect on occupational burnout. 

Coping strategies and self-efficacy could prevent occupational burnout onset, and previous systematic reviews [77,78] also supported this. However, we found that adaptive coping is particularly protective against occupational burnout. Alarcon et al., 2011 performed a meta-analysis and studied the association between job attitudes and burnout [23], and showed that adaptive organizational attitudes (such as organizational commitment) were associated with occupational burnout, which is consistent with our results, although their review included cross-sectional studies. A systematic review suggested that physical activity could reduce occupational burnout [79], which is supported by our results. However, we found a moderate quality of evidence for all the leisure subfamily (including physical activity).

Among predictors belonging to the work–family interface subfamily, occupational burnout was found to be associated only with high work–family conflict, the most studied predictor in this subfamily. A meta-analysis by Amstad et al. concluded that work interface with family and family interface with work are both related to occupational burnout [80]. While our conclusion is based on longitudinal studies exclusively, Amstad et al. also considered cross-sectional studies, which can explain the observed inconsistency between the results. Work stress was positively related to occupational burnout in this review and reinforces the concept that occupational burnout is a response to excessive stress at work [81]. 

### 4.2. Strengths and Limitations

This systematic review study has several strengths. One is the focus on exhaustion as an outcome as it is the main component of occupational burnout [10,81,82,83]. Other strengths are the inclusion of only longitudinal studies but with a different duration of follow-up and with various occupations (e.g., healthcare employees, teachers, police officers, civil servants, etc.). Since cross-sectional studies do not consider temporality [84], and therefore are inconvenient for causal inference [85], we included only longitudinal studies. This ensured that the exposure preceded the occupational burnout onset for at least 87% of the included studies. Only 13% of the included studies did not report whether the association between the predictors and exhaustion was temporal, but this was taken into account when assessing the risk of bias of the studies. Based on our results concerning the latency of occupational burnout, we recommend that future research considers a longitudinal design with multiple waves [86] with at least one-year follow-up of exposed workers. 

Finally, we managed to review occupational burnout predictors, considering their type, effect size, and role (protective versus harmful), and the overall evidence of their importance. For the quantitative synthesis, each assessment was performed independently of the other in order to avoid biased conclusions. As the vote-counting method accounting for the significance of the results is criticized, to control bias, we used the vote-counting based on the direction [87]. Moreover, we complemented the quantitative synthesis with a comprehensive risk of bias assessment and the grading of the overall quality of evidence according to PRISMA guidelines and the most validated and appropriate tools (MEVORECH and GRADE). However, we should also consider limitations when interpreting the results of this review.

Out of the 85 included studies, 34 (40%) did not control for confounding factors. The sampling method did not ensure obtain a representative sample in the majority (84%) of the studies. The included studies used a longitudinal design, but 11% did not include the same sample in all the waves. As most included studies were conducted and published before the harmonized occupational burnout definition was released, the occupational burnout measurements, even for exhaustion, were highly heterogeneous. 

The literature search was not extended to the gray literature for three main reasons: there is no consensus on a standardized method for conducting these searches, the full-text studies may be unavailable after the initial search has taken place, and the gray literature is not published in peer-reviewed journals, which is a fundamental indicator of quality [88]. 

Due to a large number of references screened and reviewed, on the one hand, and the multiple methodological approaches implemented in this review on the other hand, several studies were published during or after compiling this review. When checking databases for new publications up to November 2020, thirteen eligible studies were identified, and four new predictors in addition to the 261 predictors that we reviewed [89,90,91,92]. Due to time and resource constraints, these studies were not reviewed. However, their results were assessed, and we believe that their inclusion would not change the results and conclusions of the present review. 

### 4.3. Study Implications and Further Perspectives

Predictors with protective effects, e.g., job resources, could act as a buffer for the harmful effects of other predictors, e.g., job demands [93]; this means that increasing some predictors with protective effects, such as social support, could reduce mental health problems among workers even with high “Job demands” [28,94]. Hence, decreasing harmful factors may not necessarily increase protection, and it may not be sufficient to reduce predictors with harmful effects without increasing predictors with protective effects [95]. A recent systematic review of preventive interventions with work-focused components showed that implementing these interventions has economic benefits for employers and society through reducing sick leave duration and accelerating recovery from mental health conditions such as depression or improving supervisors’ communication with employees suffering from mental health problems [96]. Nevertheless, preventive interventions can also take into account personal-focused components along with the work-focused ones. Therefore, combined interventions are more beneficial [97,98]. Occupational burnout results in low self-esteem, feelings of guilt, dissatisfaction with the work, reduction in the quality of work, absenteeism, intention to quit the job, turnover, family problems, work–home conflict, and reduction in the quality of life [99,100]. Thus, it is beneficial to implement and evaluate strategies targeting the including protective factors (i.e., predictors with protective effects) and reducing risk factors (i.e., predictors with harmful effects).

The need to improve the methodological quality of future studies addressing occupational burnout etiology is an important research avenue. All the included studies used self-assessment instruments for both exposures (predictors) and outcome (occupational burnout), and this can produce a common method bias [101]. Using more objective hetero-evaluation methods along with the most validated PROMs for occupational burnout [11] is a priority for this area of research. Future research should address all the above-mentioned methodological issues and focus on longitudinal studies with multiple waves of at least one year. Unanswered questions and inconsistencies between results, e.g., age and sex effect [102], should also be addressed.

Before concluding, it is noteworthy that the Maslach Burnout Inventory (MBI), by far the most widely used measure of occupational burnout, is largely “preset” to correlate with job-related factors. Indeed, many MBI items involve causal attributions to work (e.g., “I feel burned out from my work”; “I feel frustrated by my job”; see Maslach et al., 2016 [103]). Because many MBI items relate burnout symptoms to work-related determinants in their very content, MBI-based research on the links between burnout and job-related factors is at risk of producing self-fulfilling predictions. It is worth bearing this in mind when interpreting our findings as well as previous findings pertaining to burnout and its job-related predictors. 

## 5. Conclusions

Preventive interventions for occupational burnout might benefit from intervening on the established predictors regarding the promotion of adaptive coping and leisure and reducing job demands and negative job attitudes. More research on the other predictors using high methodological standards is necessary to increase the scientific evidence regarding burnout etiology and prevention.

## Figures and Tables

**Table 1:** Summary of quantitative and qualitative analysis of the included studies predictors grouped per (sub)family.

Studies Predictors Grouped per (Sub)Family	Overall Risk of Bias Results	Inconsistency	Indirectness	Imprecision	Overall Quality of Evidence ^1^	Number of Studies	Binomial Test ^2,3^	Effect Sizes Range ^3^
Job demands								
Job demands (overall)	Moderate	No	No	No	Moderate	6	Harmful (0.02)	0.1–0.33
Cognitive demands	Moderate	Yes	No	Yes	Very low	3	0.5	0.01–0.11
Physical demands	High	No	No	Yes	Low	2	0.25	0.06–0.09
Quantitative demands	Low	Yes	No	Yes	Low	24	Harmful (0.00)	0.01–1.14
Emotional demands	Moderate	No	Yes	Yes	Very Low	11	Harmful (0.01)	0.07–0.52
Decision latitude (job control)								
Decision latitude	High	No	No	Yes	Low	9	0.09	0.01–0.25
Skill discretion	Low	Yes	Yes	Yes	Very low	5	0.5	0.003–0.02
Decision authority	Low	Yes	Yes	Yes	Very low	5	0.19	0.01–0.09
Autonomy	Low	Yes	Yes	Yes	Very low	2	0.75	0.06–0.09
Job resources								
Job resources	Moderate	Yes	No	Yes	Very Low	19	Protective (0.03)	0.02–0.56
Lack of job resources	High	No	No	No	Moderate	4	0.06	0.07–0.56
Interactions at work								
Social support	Moderate	Yes	Yes	Yes	Very low	21	Protective (0.04)	0.0004–0.32
Good interpersonal relations	Low	Yes	No	Yes	Very low	6	0.34	0.01–0.17
Social hindrance	Low	Yes	Yes	Yes	Very low	11	Harmful (0.03)	0.002–0.69
Communication								
Informational climate	Moderate	No	No	Yes	Low	8	0.15	0.02–0.25
Conflicting/ poor communication	Moderate	No	Yes	Yes	Very Low	5	Harmful (0.03)	0.02–0.32
Leadership								
Enriching leadership	Moderate	Yes	Yes	Yes	Very low	5	0.5	0.04–0.17
Non collaborative leadership	High	Yes	No	Yes	Very Low	3	0.5	0.05–0.1
Personality								
Unvalued trait/characteristics	Moderate	No	No	Yes	Low	2	0.25	0.02
Valued trait/characteristics	Moderate	Yes	Yes	Yes	Very low	15	0.15	0.0001–0.52
Coping								
Adaptive coping	Low	No	No	Yes	Moderate	6	Protective (0.02)	0.002–0.03
Maladaptive coping	Low	No	No	Yes	Moderate	4	0.31	0.11–0.20
Self-evaluation								
Self-esteem	Low	Yes	Yes	Yes	Very low	6	Protective (0.02)	0.02–0.41
Self-efficacy	Moderate	Yes	Yes	Yes	Very low	9	0.08	0.01–0.39
Job attitude								
Positive job attitude	Low	No	Yes	Yes	Very low	8	Protective (0.00)	0.0001–0.14
Negative job attitude	Low	No	No	Yes	Moderate	9	Harmful (0.02)	0.03–0.24
Intrinsically motivated behavior	Low	Yes	No	Yes	Low	8	0.36	0.005–0.02
Extrinsically motivated behavior	Low	Yes	No	Yes	Low	6	0.11	0.003–0.23
Personal events								
Leisure	Low	No	Yes	No	Moderate	5	Protective (0.03)	0.03–0.19
Stressful life events	Low	No	Yes	Yes	Low	5	0.19	0.07–0.51
Work family interface								
Family–work conflict	Low	No	No	Yes	Moderate	3	0.13	0.03–0.3
Work–family conflict	Low	Yes	Yes	No	Low	13	Harmful (0.00)	0.07–0.27
Family–work enrichment	Low	No	No	Yes	Moderate	1	0.5	0.005–1.08
Work–family enrichment	Low	Yes	No	Yes	Low	3	0.5	0.002–0.02
Value congruence	Low	Yes	No	Yes	Low	3	0.5	0.04–0.59
Perceived intermediate work consequences								
Stress	Low	No	Yes	Yes	Low	10	Harmful (0.05)	0.003–0.66
Satisfaction	Low	No	Yes	No	Moderate	3	0.13	0.23–0.25

^1^ Based on the GRADE, which takes into account the risk of bias, inconsistency, indirectness, and imprecision of all studies for a given predictor; ^2^ if the value of this test is <0.05 then the effect is significant and for values > the effect is not significant, ^3^ based on the Cohen’s f2 formula; an effect size less than or equal to 0.02, 0.15, 0.35 can be considered as “small”, “medium”, and “large”, respectively.

## Supplementary Materials

The following are available online at https://www.mdpi.com/1660-4601/18/17/9188/s1, Figure S1: The full literature search strategy; Table S1: Description of the included studies in the systematic review; Table S2: Description of the grouping of predictors into (sub)families with the theory behind; Table S3: The plots of z-scores per predictor.
