# Supplementary material for: Predictors of Occupational Burnout: A Systematic Review
Source: Int J Environ Res Public Health. 2021 Aug 31;18(17):9188. doi: 10.3390/ijerph18179188 (PMC8430894; doi:10.3390/ijerph18179188)
Supplement: Supplementary file 1 [file ijerph-18-09188-s001.zip › Figure S1.pdf]

# ▲	Searches	Results
1	(Burnout or burn-out).mp.	25667
2	limit 1 to human	23479
3	(occupation* or job* or employment or work-related or working or "at work" or workplace* or work-place* or "work place" or worke* or workload or "work site*" or worksite* or industry or organizational characteristics).mp.	1398137
4	2 and 3	17323
5	(burnout measure or MBS or BM or Copenhagen Burnout Inventory or CBI or Maslach Burnout Inventory or MBI or Shirom Melamed Burnout Measure or SMBM or Oldenburg Burnout Inventory or OLBI or Utrechts Burn-out Schaal or UBOS or teacher attitude scale or TAS or staff burnout for health professionales or SBS-HP or meier burnout assessment or MBA or emer-luck burnout scale or ELBOS or energy depletion index or EDI or nursing stress scale or TSI or teacher stress inventory or TSI or psychologist's burnout inventory or PBI or medical personnel stress survey or MPSS or burnout clinical subtype questionnaire or BCSQ-36).mp.	53641
6	4 and 5	5450
7	validation.mp.	270251
8	6 and 7	149
9	(cohort or longitudinal study or longitudinal studies or longitudinal test* or longitudinal examination* or longitudinal analys* or longitudinal survey*).mp.	755844
10	4 and 9	711
11	6 and 9	294
