# Supplementary material for: Predictors of Occupational Burnout: A Systematic Review
Source: Int J Environ Res Public Health. 2021 Aug 31;18(17):9188. doi: 10.3390/ijerph18179188 (PMC8430894; doi:10.3390/ijerph18179188)
Supplement: Supplementary file 1 [file ijerph-18-09188-s001.zip › Table S1.pdf]

Author, year, country, population	Outcome assessment	Exposure definition	Exposure assessment	Beta coefficient	Standard error	p-value	Effect	Effect size numeric value	Effect size label	Risk of bias result
(Adriaenssens et al., 2015),Belgium, Emergency room nurses, n= 170	MBI (Dutch version), NR	Job demands (9 items)<br>Job control (8 items)<br>Social support (8 items)<br>Material recourses (3 items)<br>Social harassment (4 items)<br>Reward (8 items)<br>Work agreements (4 items)	LQWQ-N: from 1 (totally disagree) to 4 (totally agree)	0.17<br>0.01<br>-0.07<br>-0.01<br>-0.05<br>0.05<br>0.01	0.07<br>0.03<br>0.02<br>0.04<br>0.03<br>0.03<br>0.04	0.02<br>NR<br>NR<br>NR<br>NR<br>NR<br>NR	Harmful *<br>Harmful<br>Protective*<br>Protective<br>Harmful<br>Protective<br>Harmful	0.1<br>0.08<br>0.02<br>0.07<br>0.002<br>0.16<br>0.25	Small<br>Small<br>Small<br>Small<br>Small<br>Medium<br>Medium	Low
(Angelo & Chambel, 2015), Portugal, firefighters, n=1610	MBI (the general version), NR	Conflicts and interpersonal problems	Organizational demands scale constructed by the authors.	0.1	0.04	0.02	Harmful*	0.05	Small	High
(Birkeland et al., 2018), Norway, technical workers, n= 1263	MBI-GS (the Norwegian version), NR	Harmonious passion<br>Obsessive passion<br>Support from supervisor<br>Support from colleagues	The Passion Scale (Vallerand et al., 2003), which included 12 items, half of which measured harmonious passion and the other half-obsessive passion. Support was assessed with four questions from the Perceived Organizational Support Questionnaire.	0.09<br>0.09<br>-0.03<br>-0.02	0.01<br>0.01<br>0.01<br>0.01	NR<br>NR<br>NR<br>NR	Protective*<br>Harmful *<br>Protective*<br>Protective	0.1<br>0.05<br>0.07<br>0.03	Small<br>Small<br>Small<br>Small	Low
(Boamah et al., 2017), Canada, new graduate nurses, n=405	MBI=GS, NR	Lack of personnel resources	A single-item, which assesses how often working short-staffed, affects nurses' ability to provide quality patient care.	0.14	0.05	0.01	Harmful*	0.07	Small	Low

(Childs & Stoeber, 2012), UK, healthcare service provision employees, n=69	MBI-GS, NR	Self-oriented perfectionism<br>Socially prescribed perfectionism	Socially prescribed perfectionism: 15-item scale from the Multidimensional Perfectionism Scale (MPS; Hewitt & Flett, 1991, 2004). Self-oriented perfectionism: the 15-item scale.	0.05<br>0.16	0.03<br>0.12	0.2<br>0.2	Harmful<br>Harmful	0.05<br>0.23	Small<br>Medium	Moderate
(Chrisopoulos et al., 2010), Australia, police officers, n=179	MBI-GS, NR	Cognitive demands<br>Emotional demands<br>Physical demands<br>Cognitive resources<br>Physical resources<br>Emotional support	Demand-Induced Strain Questionnaire (DISQ): each of the tasks rated on a five-point scale (1=very rarely/never, 5= very often/always).	0.1<br>0.03<br>0.04<br>0.05<br>-0.02<br>-0.1	0.08<br>0.07<br>0.07<br>0.1<br>0.09<br>0.09	NR<br>NR<br>NR<br>NR<br>NR<br>NR	Harmful<br>Harmful<br>Harmful<br>Harmful<br>Protective<br>Protective	0.11<br>0.15<br>0.09<br>0.28<br>0.21<br>0.25	Small<br>Small<br>Small<br>Medium<br>Medium<br>Medium	Moderate
(Dahlin et al., 2010), Sweden, physicians, n=186	OLBI the (Swedish translation), NR	Working hours<br>Positive learning climate<br>Performance-based self-esteem<br>Worries about endurance/competence	Performance based self-esteem (4 items)<br>Worries about future endurance/competence (3 items)<br>Learning climate (10 items)	0.008<br>-0.03<br>0.07<br>0.17	0.005<br>0.01<br>0.06<br>0.05	NR<br>0.02<br>0.001<br>0.2	Harmful<br>Protective*<br>Harmful<br>Harmful*	NA<br>NA<br>NA<br>NA	NA<br>NA<br>NA<br>NA	High
(Dubois et al., 2014), Canada, health and social service employees, n=96	MBI, NR	Autonomy<br>Skill discretion<br>Decision authority<br>Support from supervisor<br>Support from colleagues	Autonomy was assessed using three items from the Job Descriptive Scale. Decision latitude and skill discretion were measured with the corresponding subscales of the Conditions of Work Effectiveness Questionnaire-II. Support from colleagues was measured with the three-item scale from Podsakoff and MacKenzie, and supervisor support with the six items used by Stinglhamber and Vandenberghe.	-0.36<br>-0.24<br>-0.45<br>-0.13<br>-0.32	0.16<br>0.10<br>0.12<br>0.12<br>0.14	0.02<br>0.02<br>0.0001<br>NR<br>NR	Protective*<br>Protective*<br>Protective*<br>Protective<br>Protective*	0.06<br>0.01<br>0.02<br>0.05<br>0.004	Small<br>Small<br>Small<br>Small<br>Small	Moderate

(Fernet et al., 2010), Canada, college employees, n=276	MBI (The French-Canadian version), NR	Quality of social interactions at work<br>Self-determined work motivation	The short version of the Blais Work Motivation Inventory. 4-item Quality of Interpersonal Relationships Scale.	-0.13<br>-0.02	0.05<br>0.01	0.01<br>0.2	Protective*<br>Protective	0.13<br>0.02	Small<br>Small	Moderate
(Fernet et al., 2014), Canada, teachers, n=175	MBI (The French-Canadian version), NR	Harmonious passion<br>Obsessive passion	The short form (Lafrenière et al., 2013) of the Passion Scale (Vallerand et al., 2003)	-0.54<br>0.28	0.02<br>0.1	0.01<br>0.01	Protective*<br>Harmful *	0.11<br>0.23	Small<br>Medium	Moderate
(Feuerhahn et al., 2013), Germany, teachers, n=56	MBI-ES (the German version), NR	Conflicts and interpersonal problems<br>Emotional dissonance<br>Emotional support<br>Classroom disruption<br>Time pressure<br>Self-efficacy	The teacher-specific measure Fragebogen zur Arbeitssituation an Schulen (Kaempf & Krause, 2004). Frankfurt Emotion Work Scales (Zapf et al., 1999). Four items of the Instrument zur stressbezogenen Tätigkeitsanalyse (ISTA) developed by Semmer, Zapf, and Dunckel (1999). Seven items from the teacher-specific measure FASS developed by Krause and colleagues (Kaempf & Krause, 2004). A self-efficacy scale developed specifically for teachers by Schmitz and Schwarzer (2000). The social support questionnaire Fragebogen zur Sozialen Unterstützung (F-SozU; Fydrich, Sommer, & Brähler, 2002).	0.01<br>0.02<br>-0.21<br>0.38<br>-0.03<br>-0.69	0.17<br>0.14<br>0.13<br>0.18<br>0.13<br>0.23	NR<br>NR<br>NR<br>NR<br>NR<br>NR	Harmful<br>Harmful<br>Protective<br>Harmful*<br>Protective<br>Protective*	0.59<br>0.51<br>0.19<br>0.69<br>0.12<br>0.39	Large<br>Large<br>Medium<br>Large<br>Small<br>Large	Moderate

(Fida et al., 2018), Canada, nurses, n=596	MBI-GS, NR	Work incivility from supervisor<br>Work incivility from coworker<br>Work incivility from physician<br>Relational self-efficacy	Incivility: the Straightforward Incivility Scale (Leiter & Day, 2013). Self-efficacy: the relational subscale of the Occupational Coping Self<Efficacy Questionnaire for Nurses (Pisanti et al., 2008)	0.02<br>0.13<br>0.23<br>-0.16	0.01<br>0.06<br>0.09<br>0.06	0.2<br>0.02<br>0.01<br>0.01	Harmful<br>Harmful*<br>Harmful*<br>Protective*	0.02<br>0.08<br>0.08<br>0.04	Small<br>Small<br>Small<br>Small	Low
(Figueiredo-Ferraz et al., 2012), Spain, nursing professionals, n=316	MBI (the Spanish version), NR	Work satisfaction	"Satisfaction Questionnaire S20/23" (Melia and Peiró, 1989): 11 items.	-0.09	0.07	0.2	Protective	0.25	Medium	High
(Firoozabadi et al., 2018), Iran, health and social welfare employees, n=123	OLBI, NR	Affective rumination<br>Problem solving	Work-Related Rumination Questionnaire (Cropley, Michalianou, Pravettoni, & Millward, 2012).	0.23<br>-0.19	0.1<br>0.08	0.02<br>0.02	Harmful*<br>Protective*	0.11<br>0.001	Small<br>Small	Moderate
(Fong, 1993), USA, nursing educators, n=84	MBI, NR	Work and time demands	Mueller's role overload scale: a 4-point Likert scale ranging from 1 (not a source of pressure) to 4 (a great source of pressure)	0.39	0.17	0.02	Harmful*	NA	NA	High
(Fritz & Sonnentag, 2005), Germany, emergency medical services employees, n=87	OLBI, NR	Social activity<br>Positive work reflection<br>Non-work hassles	Social activity (3 items)<br>Positive work reflection (3 items)<br>Non-work hassles: a measure developed by Bolger et al. (9 items).	-0.14<br>-0.17<br>-0.08	0.01<br>0.09<br>0.06	0.2<br>0.05<br>0.2	Protective<br>Protective<br>Protective	0.19<br>0.14<br>0.2	Medium<br>Small<br>Medium	High

(Fritz & Sonnentag, 2006), Germany, non-academic university employees, n=221	OLBI, NR	Positive work reflection<br>Negative work reflection<br>Non-work hassles<br>Relaxation<br>Mastery<br>Workload after vacation	Workload after vacation: seven items were adapted from Karasek's (1979) Negative work reflection (3 items). Non-work hassle by adapting nine items from a measure developed by Bolger, DeLongis, Kessler, and Schilling (1989). Positive work reflection (3 items). Mastery (3 items). Relaxation (3 items).	0.04<br>0.16<br>0.05<br>-0.07<br>-0.03<br>0.18	0.03<br>0.06<br>0.03<br>0.05<br>0.02<br>0.07	0.2<br>0.01<br>0.2<br>0.2<br>0.2<br>0.01	Protective<br>Harmful*<br>Harmful<br>Protective<br>Protective<br>Harmful*	0.002<br>0.24<br>0.07<br>0.03<br>0.0001<br>0.15	Small<br>Medium<br>Small<br>Small<br>Small<br>Small	High
(Fusilier & Manning, 2005), USA, chemical corporation and health insurance employees, n=260	MBI, NR	Stress<br>Social support<br>Hardiness<br>Type A behavior<br>Stressful life events<br>Physical exercises	Stressful life events were measured by the Life Events Inventory (Cochrane and Robertson, 1973). Type A Behavior.(9-item) type A personality scale developed by Sales. Hardiness. This was measured by five scales developed through prior research of Kobasa. Stressful Work Events were assessed using the Organizational Readjustment Rating Scale. Social support was assessed with the Social Support Questionnaire (SSQ) developed by Sarason et al. A measure of physical exercise was indexed by having respondents check various forms of exercise and their frequency and	0.05<br>-0.02<br>-0.09<br>0.20<br>0.13<br>-0.06	0.04<br>0.02<br>0.07<br>0.08<br>0.06<br>0.05	0.2<br>0.2<br>0.2<br>0.01<br>0.02<br>0.2	Harmful<br>Protective<br>Protective<br>Harmful*<br>Harmful*<br>Protective	NA<br>NA<br>NA<br>NA<br>NA<br>NA	NA<br>NA<br>NA<br>NA<br>NA<br>NA	Low

			duration of participation on a weekly basis.							
(Garbarino et al., 2013), Italy, police officers, n=289	MBI (Italian version), >23 on Professional Exhaustion (PE)	Job demands<br>Work and time demands<br>Decision latitude<br>Reward<br>Over-commitment<br>Extraversion<br>Agreeableness<br>Conscientiousness<br>Emotional stability<br>Openness	BFQ: a five-point Likert-type scale ranging from complete disagreement (1, absolutely false for me) to complete agreement (5, very true for me).<br>DCS consists of three sub-scales: psychological job demand, (5 items), job control/decision latitude (6 items), social support (6 items).	0.12<br>0.21<br>-0.09<br>-0.21<br>0.27<br>-0.09<br>-0.09<br>-0.04<br>-0.21<br>0.03	0.05<br>0.19<br>0.07<br>0.06<br>0.08<br>0.07<br>0.07<br>0.03<br>0.06<br>0.02	0.02<br>0.02<br>0.2<br>0.001<br>0.001<br>0.2<br>0.2<br>0.2<br>0.001<br>0.2	Harmful*<br>Harmful*<br>Protective<br>Protective*<br>Harmful*<br>Protective<br>Protective<br>Protective<br>Protective*<br>Harmful	NA<br>NA<br>NA<br>NA<br>NA<br>NA<br>NA<br>NA<br>NA<br>NA	NA<br>NA<br>NA<br>NA<br>NA<br>NA<br>NA<br>NA<br>NA<br>NA	Moderate
(Gelsema et al., 2006), the Netherlands, nurses, n=381	MBI (the Dutch client version), NR	Work and time demands<br>Physical demands<br>Nurse-doctor collaboration<br>Material resources<br>Personal resources<br>communication / information flow<br>Work agreements<br>Reward	LQWLQ-N: from 1 (totally disagree) to 4 (totally agree)	0.001<br>0.16<br>0.05<br>-0.01<br>0.16<br>-0.06<br>-0.08<br>-0.03	0.0007<br>0.06<br>0.04<br>0.007<br>0.12<br>0.05<br>0.06<br>0.02	0.2<br>0.01<br>0.2<br>0.2<br>0.2<br>0.2<br>0.2<br>0.2	Harmful<br>Harmful*<br>Harmful<br>Protective<br>Protective<br>Protective<br>Harmful<br>Harmful	0.11<br>0.06<br>0.02<br>0.03<br>0.04<br>0.04<br>0.05<br>0.02	Small<br>Small<br>Small<br>Small<br>Small<br>Small<br>Small<br>Small	Low
(Gil-Monte & Garcia-Juesas, 2008), Spain, nurses, n=316	MBI, NR	Work and time demands<br>Self-efficacy	Work and time demands: the Spanish adaption of the Karasek scale (7 items), the sclae ranging from 1 "Very often" and 5 "Very rarely."<br>Self-efficacy: the Spanish adaptation of the Baessler scale and Schwarzer (9 items). The scale was completed through a 5-degree frequency format in which 1	0.12<br>-0.06	0.05<br>0.05	0.02<br>0.2	Harmful*<br>Protective	0.21<br>0.05	Medium<br>Small	Low

			"Strongly disagree" and 5 "Strongly agree".							
(Goddard et al., 2006), Australia, teachers, n=316	MBI, NR	Workplace innovation<br>Time pressure	Work Environment Scale (WES; Moos, 1994) modified for teacher respondents according to Fisher and Fraser (1983, 1991).	-0.3<br>0.29	0.08<br>0.09	0.0001<br>0.001	Protective*<br>Harmful*	0.86<br>1.14	Large<br>Large	High
(Gonzalez-Morales et al., 2010), Spain, teachers, n=555	MBI-GS (the Spanish version), NR	Social support coping<br>Direct action coping<br>Teaching stressors	For the coping strategies, two different dimensions were built from the original Occupational Stress Indicator (OSI) [Cooper, Sloan, & Williams, 1988]. Dimensions. Teaching stressors were measured by means of an experimental scale built from a previous qualitative study in which various focus groups were undertaken to identify the sources of stress experienced by teachers (Peiro´, Rodrı´guez, & Bravo, 2003). This scale was composed of 15 items.	0.08<br>-0.004<br>-0.03	0.09<br>0.13<br>0.02	0.39<br>0.97<br>0.63	Harmful<br>Protective<br>Protective	0.004<br>0.03<br>0.15	Small<br>Small<br>Medium	Low

(Gonzalez-Morales et al., 2012), Spain, teachers, n=555	MBI-GS (the Spanish version), NR	Personnel resources<br>Material resources<br>Absenteeism<br>Colleagues/team exhaustion<br>Workload stressors	The indicator of physical resources comprised four items (classrooms, teachers' rooms, playground, and other facilities). Personal resources were computed by dividing the number of teachers in a school by the number of students in the school; therefore it is a continuous variable from 0 (1 teacher–∞ students) to 1 (1 teacher–1 student). Absenteeism: the percentage of non-worked days in a school due to sickness in relation to the total annual working days. The measure for team exhaustion was built following the referent-shift consensus model (Chan, 1998). The items of this measure are the result of the transformation of the MBI-GS burnout measure from an individual referent to a group-level. According to Lyne, Barrett, Williams, and Coaley's (2000) psychometric evaluation of the Occupational Stress Indicator (OSI; Cooper, Sloan, & Williams, 1988) and the	-6<br>-0.03<br>-0.02<br>0.33<br>0.09	3.58<br>0.07<br>0.02<br>0.15<br>0.07	0.1<br>0.7<br>0.25<br>0.03<br>0.16	Protective<br>Protective<br>Protective<br>Harmful*<br>Harmful	NA<br>NA<br>NA<br>0.15<br>0.08	NA<br>NA<br>NA<br>Medium<br>Small	Low

			revised score key they propose, the 61 items of the ‘‘Sources of Pressure’’ scale can be scored in three dimensions: managerial pressures, employee pressures, and workload.							
(Gregersen et al., 2014), Germany, healthcare employees, n=339	MBI (the German version), NR	Transformational leadership<br>Occupational self-efficacy	Occupational self-efficacy was assessed by a short version of the scale by Rigotti et al. (2008). Transformational leadership: a version of the MLQ5X-Short by Bass and Avolio (1995), as translated and modified by Felfe (2006).	-0.061<br>-0.024	0.05<br>0.02	0.2<br>0.2	Protective<br>Protective	0.04<br>0.07	Small<br>Small	Moderate
(Gregory & Menser, 2015), USA, physicians, n=153	MBI, NR	Value congruency<br>Workload	AWS (6 key dimensions)	-0.61<br>1.29	0.16<br>0.33	0.0001<br>0.0001	Protective*<br>Harmful*	NA<br>NA	NA<br>NA	High
(Hakanen et al., 2008), Finland, dentistry, n=3035	MBI, NR	Job demands<br>Job recourses	DEWSS (8 items). DEJRS: Craftsmanship (3 items) and Professional contacts (4 items), Long-term and immediate results (6 items). All items were scored from 1 ‘‘very little or not at all’’ to 5 ‘‘very much.’	0.23<br>(-0.05)	0.07<br>0.02	0.01<br>0.02	Harmful*<br>Protective*	NA<br>NA	NA<br>NA	Low

(Hertzberg et al., 2016), Norway, doctors, n=274	OLBI, NR	Time pressure<br>Working hours<br>Emotional demands<br>Support from partners<br>Support from colleagues<br>Work-family conflict	The number of working hours per week included the regular work schedule as well as self-imposed or imposed overtime. Emotional demands, time pressure, and work-home conflict was measured using a modified and validated version of Cooper's Job Stress Questionnaire. Collegial support was assessed on a scale from 1 (not at all) to 7 (a very high degree) using two previously validated questions.	0.12<br>0.06<br>0.1<br>-0.05<br>-0.06<br>-0.15	0.07<br>0.07<br>0.07<br>0.41<br>0.04<br>0.05	0.09<br>0.4<br>0.19<br>NR<br>NR<br>0.001	Harmful<br>Harmful<br>Harmful<br>Protective<br>Protective<br>Protective*	NA<br>NA<br>NA<br>NA<br>NA<br>NA	NA<br>NA<br>NA<br>NA<br>NA<br>NA	Moderate
(Hochwalder, 2008), Sweden, health care employees, n = 838	MBI (Swedish translation), NR	Psychological empowerment	Psychometrically evaluated Swedish translation of Spreitzer's empowerment scale.	0.15	0.06	0.01	Harmful*	0.11	Small	Low
(Hornung et al., 2013), Germany, physicians, n=95	MBI (German version), NR	Workload<br>Stressful interactions with patients<br>Work-family conflict	Work overload and patient demands were assessed with two 3-item scales by Bssing and Glaser. Work-family conflict was measured with Netemeyer et al.'s (1996) 5-item scale. All three scales used a 5-point response format (1 = Not at all to 5 = To a very great extent).	-0.05<br>-0.05<br>0.11	0.09<br>0.02<br>0.09	0.59<br>0.01<br>0.2	Protective<br>Protective<br>Harmful*	0.07<br>0.08<br>0.27	Small<br>Small<br>Medium	Moderate
(Huang et al., 2012), Taiwan, customs office employees, n=299	MBI-GS, NR	Work and time demands<br>Job control	The measures of job demands and job control that we used were developed and validated by Van Veldhoven (1996) in	0.05<br>-0.06	0.04<br>0.05	0.2<br>0.2	Harmful<br>Protective	0.16<br>0.1	Medium<br>Small	Low

			his dissertation research. A four-point response scale (1 : 'never', 2 : 'sometimes', 3: 'often', 4 : 'always')							
(Hudek-Knezevic et al., 2011), Croatia, female hospital nurses, n= 118	MBI "Croatian language", NR	Extraversion<br>Agreeableness<br>Conscientiousness<br>Neuroticism<br>Openness to experience<br>Organizational commitment<br>Job demands (role conflict and workload)<br>Continuance commitment	Personality measure. Big Five Inventory (BFI). Perceived Organizational Stress Inventory was developed by combining items of several well-known questionnaires measuring work overload, role conflict, and role ambiguity as predictors of stress at work. It consist of 15 items on a five-point scale.. The organizational commitment questionnaire was translated and adapted to Croatian language. It consists of 18 items measuring different aspects of commitment to work organization. Answers are scored on a 5-point scale (1 – definitely disagree; 5 – strongly agree).	0.17<br>(-0.06)<br>0.07<br>0.07<br>0.05<br>-0.21<br>-0.1<br>0.47	0.1<br>0.1<br>0.11<br>0.11<br>0.1<br>0.1<br>0.1<br>0.12	0.09<br>0.53<br>0.53<br>0.53<br>0.6<br>0.05<br>0.31<br>0.0001	Harmful<br>Protective<br>Harmful<br>Harmful<br>Harmful<br>Protective*<br>Harmful*<br>Protective	0.02<br>0.02<br>0.0001<br>0.02<br>0.001<br>0.06<br>0.23<br>0.00	Small<br>Small<br>Small<br>Small<br>Small<br>Small<br>Medium<br>Small	Moderate
(Idris et al., 2014), Malaya, private sector employees, n=117	MBI (Malay translation), NR	Emotional demands<br>Managment commitment & communication	The Copenhagen Psychosocial Questionnaire (COPSOQ; Kristensen, Hannerz, Hogh, & Borg, 2005). The 12-item PSC scale (PSC-12) (Hall, Dollard, & Coward, 2010).	0.17<br>-0.25	0.08<br>0.1	NR<br>0.02	Harmful*<br>Protective*	0.07<br>0.05	Small<br>Small	Moderate

(Innstrand et al., 2008), Norway, church ministers, n=308	OLBI (the Norwegian version), NR	Work-home conflict<br>Work-home facilitation<br>Family-work conflict<br>Family-work facilitation	Work family interaction (WFI) was assessed using a four-dimensional scale from a study by Wayne, Musisca, and Fleeson (2004). The instrument was translated into Norwegian by one of the authors.	0.34<br>-0.24<br>0.06<br>-0.21	0.06<br>0.05<br>0.02<br>0.07	0.2<br>0.2<br>0.2<br>0.2	Harmful*<br>Protective*<br>Harmful<br>Harmful	0.17<br>0.03<br>0.03<br>0.005	Medium<br>Small<br>Small<br>Small	High
(Innstrand et al., 2011), Norway, various working populations, n=2235	OLBI (Norwegian version), NR	Performance-based self-esteem<br>Autonomy<br>goal orientation<br>Workload<br>Value congruency<br>Work-family conflict<br>Work-family facilitation<br>Family-work conflict<br>Family-work facilitation	Job performance–based self-esteem (3 items), goal-orientation (4 items), and value congruency (4 items) were developped for this study and the items were scored on a five-point scale ranging from totally disagree (1) to totally agree (5). Workload (3 items) and autonomy (4 items) scale has been used in a previous studies conducted by the Research Institute of the Norwegian Medical Association (i.e. Aasland, Olff, Falkum, Schweder, & Ursin, 1997). Work–home interaction was measured by a four-dimensional scale on work–home interaction based on the study by Wayne et al. (2004) and adapted to Norwegian conditions (Innstrand, Langballe, Falkum,	-0.06<br>0.16<br>0.03<br>-0.06<br>0.06<br>-0.14<br>0.07<br>0.03<br>0.09	0.05<br>0.06<br>0.02<br>0.05<br>0.02<br>0.15<br>0.1<br>0.03<br>0.05	0.2<br>0.01<br>0.2<br>0.2<br>0.2<br>0.02<br>0.02<br>0.02<br>0.02	Protective<br>Harmful*<br>Harmful<br>Protective<br>Harmful<br>Protective*<br>Harmful<br>Harmful<br>Harmful	0.05<br>0.09<br>0.02<br>0.15<br>0.09<br>0.16<br>0.002<br>0.05<br>0.00	Small<br>Small<br>Small<br>Medium<br>Small<br>Medium<br>Small<br>Small<br>Small	High

			Espnes, & Aasland, 2009).							
(Jensen & Knudsen, 2016), Norway, oil and gas company employees, n=1702	MBI-GS (the Norwegian translation), NR	Psychological health complaints<br>Work-family conflict	The scale measuring psychological health complaints comprised seven questions from the Subjective Health Complaint Inventory. Five items developed by Netemeyer, Boles, and McMurrian (1996) measured Wrok-home conflict.	0.16<br>0.07	0.05<br>0.02	0.001<br>0.001	Harmful*<br>Harmful*	0.79<br>0.14	Large<br>Small	High
(Jimenez & Dunkl, 2017), Austria, various working populations, n=141	MBI-GS (the German version), NR	Value congruency<br>Workload<br>Quality of social interactions at work<br>Job control<br>Job resources<br>Fairness<br>Reward	AWS (6 key dimensions)<br>RESTQ-Work (27 items)	0.02<br>0.17<br>0.02<br>-0.02<br>-0.02<br>-0.06<br>-0.08	0.08<br>0.08<br>0.06<br>0.09<br>0.09<br>0.09<br>0.09	0.82<br>0.04<br>0.76<br>0.82<br>0.82<br>0.36<br>0.50	Harmful<br>Harmful*<br>Harmful<br>Protective<br>Protective<br>Protective<br>Protective	0.17<br>0.51<br>0.06<br>0.16<br>0.28<br>0.17<br>0.28	Medium<br>Large<br>Small<br>Medium<br>Medium<br>Medium<br>Medium	Moderate
(Konze et al., 2017), Germany, energy supplying employees, n=139	MBI (the German translation), NR	Work and time demands<br>Emotional dissonance<br>Job control	Work and time demands (3 itwms based on the Short Questionnaire for Job Analysis by Prümber and colleagues)<br>Emotional dissonance (5 items from the Frankfurt Emotion Work Scale)<br>Job control was measured by combining 3 items from the timing control and method control (4 items) subscales developed by Jackson and colleagues.	0.07<br>0.18<br>0.14	0.05<br>0.05<br>0.05	NR<br>NR<br>NR	Harmful<br>Harmful*<br>Harmful	0.11<br>0.32<br>0.07	Small<br>Medium<br>Small	Moderate

(Korunka et al., 2015), Austria, eldercare workers, n=587	MBI (German version), NR	Work and time demands<br>Cognitive demands	Each type of the demands was measured with three items adapted from Ulferts et al. The respondents had to indicate if the respective demand had decreased (1: decreased strongly) or increased (5: increased strongly) in recent years.	0.34<br>-0.25	0.1<br>0.07	0.001<br>0.001	Harmful*<br>Harmful*	0.06<br>0.01	Small<br>Small	Moderate
(Kubicek & Korunka, 2015), Austria, employees in nursing homes or outpatient care organizations, n=559	MBI (the German version), NR	Cognitive demands<br>Emotional dissonance	Cognitive demands: 4-item subscale of Büssing and Glaser's Emotional dissonance: 5-item subscale of the Frankfurt Emotion Work Scales	-0.03<br>0.07	0.17<br>0.04	NR<br>NR	Protective<br>Harmful	0.00<br>0.1	Small<br>Small	Moderate
(Kubicek et al., 2014), Austria, employees in nursing homes for elderly people, n=591	MBI (the German version), NR	Job control	Instrument for stress-related job analysis (5 items)	-0.07	0.05	0.2	Protective	0.02	Small	Moderate
(Kutney-Lee et al., 2013), USA, nurses, n=5957	MBI, >27	Personnel resources	The Practice Environment Scale of the Nursing Work Index (PES-NWI).	-3.63	1.67	0.03	Protective*	NA	NA	Low

(Langballe et al., 2011), Norway, physicians, n=523	OLBI (the Norwegian version), NR	Work-home conflict<br>Work-home facilitation<br>Family-work conflict<br>Family-work facilitation<br>Performance-based self-esteem<br>Autonomy<br>goal orientation<br>Workload<br>Value congruency	JPBSE, GO and VC items were developed for the present study based on Hallsten's burnout theory. The workload and autonomy scales used in this study were based on the scales used in previous studies conducted by Aasland, Olff, Falkum, Schweder, and Ursin (1997) and Cooper, Rout, and Faragher (1989). Work–home interaction was measured by 12 items based on Wayne et al.'s study (2004).	M:(-0.09), F:(-0.02)<br>M:0.03, F:(-0.1)<br>M:(-0.07), F:(-0.02)<br>M:(-0.01), F:(-0.01)<br>M:(-0.05), F:(-0.09)<br>0.15 , 0.07<br>0.05 , 0.04<br>M:(-0.07),F: 0.02<br>M:0.12, F:0.11	M:0.07, F:0.02<br>M:0.02, F:0.08<br>M:0.05, F:0.02<br>M:0.08, F:0.08<br>M:0.04, F:0.07<br>M:0.06, F:0.03<br>M:0.04, F:0.03<br>M:0.06, F:0.02<br>M:0.05,F:0.05	M:0.2 , F:0.2<br>M:0.2 , F: 0.2<br>M:0.2 , F:0.2<br>M:0.2 , F:0.2<br>M:0.2 , F:0.2<br>M:0.02 ,F:0.2<br>M:0.2 , F:0.2<br>M:0.2 , F:0.2<br>M:0.02, F:0.02	Protective<br>Harmful,<br>Protective<br>Protective<br>Protective<br>Protective<br>Harmful*<br>Harmful<br>Protective,<br>Harmful<br>Harmful*	0.25<br>0.02<br>0.04<br>0.01<br>0.07<br>0.06<br>0.01<br>0.17<br>0.04	Medium<br>Small<br>Small<br>Small<br>Small<br>Small<br>Small<br>Medium<br>Small	Moderate
(Lapointe et al., 2013), Canada, various working populations, n=224	MBI-GS, NR	Psychological contract breach<br>Organizational commitment<br>Commitment to the supervisor	Robinson and Morrison's (2000) 5-item scale was used to measure psychological contract breach. Organizational commitment was measured using Bentein, Vandenberg, Vandenberghe, and Stinglhamber's (2005) version of Meyer, Allen, and Smith's (1993) 6-item scale. Stinglhamber, Bentein, and Vandenberghe's (2002) 6-item scale was used to measure affective commitment to the supervisor.	0.11<br>-0.2<br>-0.06	0.09<br>0.09<br>0.05	0.2<br>0.02<br>0.2	Harmful<br>Protective*<br>Protective	0.04<br>0.06<br>0.07	Small<br>Small<br>Small	Low

(Laugaa et al., 2008), France, French elementary school teachers, n=259	MBI, NR	Job demands<br>Conflict and interpersonal problems<br>Stress<br>Self-efficacy<br>Traditional teaching coping<br>Coping centered on the problem	The frequency of professional stressors was evaluated by using a scale specifically constructed for this research. Self-efficacy was measured with a scale by Jerusalem and Schwarzer. Coping strategies were evaluated with a validated French version of the coping specific to teachers scale by Dewe (1985).	0.17<br>0.14<br>0.28<br>-0.17<br>-0.18<br>0.13	0.07<br>0.05<br>0.11<br>0.07<br>0.05<br>0.07	0.01<br>0.01<br>0.01<br>0.01<br>0.01<br>0.01	Harmful*<br>Harmful*<br>Harmful*<br>Protective*<br>Harmful*<br>Protective*	NA<br>NA<br>NA<br>NA<br>NA<br>NA	NA<br>NA<br>NA<br>NA<br>NA<br>NA	High
(Laugaa & Bruchon-Schweitzer, 2005), France, teachers, n=410	MBI, NR	Problem oriented coping	The French version of the Dewe measurement coping questionnaire.	-0.15	NR	0.02	Protective*	NA	NA	Moderate
(Lavigne et al., 2012), Canada, professionals for the Quebec government, n=325	MBI (the validated French version), NR	Obsessive passion	Vallerand and colleagues' (2003) Passion Scale	0.15	0.06	0.01	Harmful*	0.04	Small	High
(Leiter et al., 2013), Finland, forestry workers, n=4396	MBI-GS (The Finnish version), NR	Communication / information flow<br>Predictability<br>Decision authority<br>Skill discretion	Predictability, decision authority, skill discretion: Occupational Stress Questionnaire (Elo, Leppänen, Lindström, &Roponen, 1992). Information flow was operationalized by five items (Väänänen, Pahkin, Kalimo, & Buunk, 2004).	-0.04<br>-0.01<br>-0.02<br>0.02	0.016<br>0.008<br>0.016<br>0.016	0.01<br>0.2<br>0.2<br>0.2	Protective*<br>Protective<br>Protective<br>Harmful	0.03<br>0.03<br>0.01<br>0.002	Small<br>Small<br>Small<br>Small	Moderate

(Lindeman et al., 2017), USA, general surgery residents, n=143	MBI, high burnout ≥27,moderate burnout 17-26, and low/no burnout ≤16	Agreeableness<br>Emotional stability<br>Conscientiousness<br>Emotional competences<br>Work satisfaction	The TIPIisa10-item measure of the Big Five (or Five-Factor Model) of personality The Trait Emotional Intelligence Questionnaire Short Form (TEI-Que-SF) The job resources scale of the job demands and resources questionnaire	-2.41<br>-0.76<br>0.62<br>-4.62<br>-0.67	0.94<br>1.78<br>1.09<br>1.99<br>0.23	0.01<br>0.67<br>0.57<br>0.02<br>0.003	Protective*<br>Protective<br>Harmful<br>Protective*<br>Protective*	NA<br>NA<br>NA<br>NA<br>NA	NA<br>NA<br>NA<br>NA<br>NA	Moderate
(Lizano & Barak, 2012), USA, public child welfare workers, n=335	MBI, NR	Work-family conflict<br>Support from supervisor<br>Organizational support<br>Stress	Beatty's (1996) three-item work–family conflict scale. Organizational and supervisory support variables were measured using eight items developed by Eisenberger, Huntington, Hutchison, and Sowa (1986). Stress was measured using Rizzo, House, and Lirtzman's (1970).	0.09<br>-0.03<br>-0.07<br>0.08	0.03<br>0.04<br>0.03<br>0.03	0.01<br>0.43<br>0.02<br>0.02	Harmful*<br>Protective<br>Harmful<br>Harmful	0.08<br>0.01<br>0.00<br>0.06	Small<br>Small<br>Small<br>Small	Moderate
(Lizano & Barak, 2015), USA, urban public child welfare employee, n=361	MBI-HSS, NR	Role ambiguity<br>Work-family conflict	Rizzo, House, and Lirtzman (1970) role ambiguity scale. Work–family conflict is measured using three items from Beatty (1996) work–family conflict scale.	0.14<br>0.19	0.05<br>0.07	0.01<br>0.01	Harmful*<br>Harmful*	NA<br>NA	NA<br>NA	Moderate

(Lorente Prieto et al., 2008), Spain, teachers n=274	MBI-GS (the Spanish version), NR	Work and time demands<br>Emotional demands<br>Emotional competences	Work and time demands were measured with the questionnaire of Beehr, Walsh and Taber (3 items) Emotional competences and emotional demands were measured with the questionnaire of Van Veldhoven and Meijman (6- and 7-item scales)	0.27<br>0.16<br>(-0.15)	0.05<br>0.06<br>0.06	NR<br>NR<br>NR	Harmful*<br>Harmful*<br>Protective*	NA<br>NA<br>NA	NA<br>NA<br>NA	Moderate
(Lu et al., 2013) , Taiwan, different organizations of diverse industries, n=245	MBI (the Chinese version), NR	Presenteeism	Tow items were developed to access the behavioral frequency of presenteeism, similar to the construct of “sickness presenteeism” (Aronsson et al., 2000; Demerouti et al., 2009).	0.27	0.08	0.001	Harmful*	0.005	Small	High

(Makikangas & Kinnunen, 2003), Finland, various working populations, n=457	MBI, NR	Decision latitude<br>Poor social climate<br>Job insecurity<br>Self-esteem<br>Optimism	Quantitative Workload Inventory: the subjects responded on a five-point scale (1=not at all, 5=very often). Lack of control was assessed through two scales derived by Jackson, Wall, Martin, and Davids. Job insecurity (4 items) (Caplan, Cobb, French, van Harrison, & Pinneau, 1980). Poor organizational climate was measured by a 10-item scale; the scale was a modification of items based on the study 'Quality of Working Life in Finland 1977–1997' (Lehto & Sutela, 1998; see Kinnunen & Natti, 1994; Litwin & Stringer, 1968). Self-esteem was measured by Rosenberg's (1965) 10-item scale and items were rated on a five-point Likert scale (1=totally agree, 5=totally disagree). Optimism was assessed through an abbreviated version of ''The Revised Life Orientation Test'' developed by Scheier, Carver, and Bridges (1994).	M:(-0.06) ,F:(0.08)<br>M:(-0.01) ,F:(-0.05)<br>M:(-0.06) , F:0.1<br>M:(-0.19) , F:(0.001)<br>M:(-0.12) , F:(0.09)	M:0.05 , F:0.06<br>M:0.01 , F:0.04<br>M:0.05 , F:0.08<br>M:0.06 , F:0.0007<br>M:0.05 ,F:0.07	M:0.2 , F:0.2<br>M:0.2 , F:0.2<br>M:0.2 , F:0.2<br>M:0.001 , F:0.2<br>M:0.02 , F:0.2	M: Harmful, F:Protective<br>M:Protective<br>M: Harmful, F:Protective<br>M: Harmful, F:Protective*<br>M: Harmful, F:Protective*	0.01,0.05<br>0.06,0.04<br>0.04,0.003<br>0.2,0.004<br>0.08,0.03	Small<br>Small<br>Small<br>Small , medium<br>Small	Low

(Martinez-Inigo & Totterdell, 2016), Spain., primary healthcare professionals, n=233	MBI (the Spanish version), NR	Surface acting<br>Deep acting	The surface acting measure was a five-item scale. Three items were drawn from Brotheridge and Lee's (2003) Emotional Labor Scale (ELS). Deep acting was originally measured with five items, three items from Brotheridge and Lee's (2003) ELS.	0.04<br>0.01	0.12<br>0.08	NR<br>NR	Harmful<br>Protective	0.11<br>0.00	Small<br>Small	High
(Mauno et al., 2015), Finland, university employees, n=814	MBI, NR	Work-family conflict<br>Work-family facilitation	Work-family conflict: Carlson et al., 2000<br>Work-family facilitation: Carlson et al., 2006	0.14<br>(-0.08)	0.04<br>0.034	0.001<br>0.02	Harmful*<br>Protective*	NR<br>NR	NR<br>NR	Moderate
(McManus et al., 2002), UK, doctors, n=365	An abbreviated nine-item version of the MBI, NR	Stress	The 12-item version of the general health questionnaire GHQ (GHQ-12).	0.175	0.07	0.02	Harmful*	0.25	Medium	Moderate
(Meier et al., 2015), Switzerland, general practitioners, n=246	MBI, NR	Lack of reward	Measure by Bakker and colleagues (3 item)	0.15	0.06	0.02	Harmful*	NR	NR	Low
(Park et al., 2016), USA, Veterans Health Administration, Peer specialists, n= 152	MBI, ≥21	Self-efficacy<br>Mental heatlh<br>Working hours	The General Self-Efficacy Scale<br>The Behavior and Symptom Identification Scale (BASIS-24)	0.06<br>0.29<br>(-0.08)	0.02<br>0.15<br>0.11	NR<br>NR<br>NR	Harmful*<br>Harmful<br>Protective	NR<br>NR<br>NR	NR<br>NR<br>NR	Low
(Petrou et al., 2015), the Netherlands, police officers, n=580	OLBI, NR	Sensitivity to change<br>Self-initiated resources seeking<br>Self-initiated challenges seeking<br>Self-initiated reducing demands	Job crafting was measured with items by Petrou et al. (2012).<br>Willingness to change was measured with a 4-item scale developed by Metselaar (1997).	0.09<br>(-0.03)<br>(-0.07)<br>0.08	0.03<br>0.04<br>0.03<br>0.03	0.01<br>0.43<br>0.02<br>0.02	Harmful*<br>Protective*<br>Harmful*<br>Protective*	0.03<br>0.06<br>0.03<br>0.07	Small<br>Small<br>Small<br>Small	Moderate

(Philipp & Schupbach, 2010), Germany, teachers, n=102	MBI (German version), NR	Surface acting<br>Deep acting	The three surface acting items and two deep acting items were taken from the Emotional Labor Scale (ELS, Brotheridge & Lee, 2003) and translated into German.	-0.12<br>-0.26	0.09<br>0.1	0.2<br>0.01	Protective<br>Protective*	0.20<br>0.02	Medium<br>Small	Moderate
(Pisanti et al., 2016), Italy, nurses, n=217	MBI-HSS (Italian version), NR	Work and time demands<br>Social support<br>Decision latitude	LQWLQ-N: from 1 (totally disagree) to 4 (totally agree)	-0.04<br>0.22<br>-0.08	0.03<br>0.07<br>0.06	0.2<br>NR<br>0.2	Protective<br>Harmful*<br>Protective	0.01<br>0.0004<br>0.03	Small<br>Small<br>Small	Moderate
(Pomaki et al., 2009), the Netherlands, nurses, n=172	MBI (the Dutch version), the cutoff point is 18	Goal self-efficacy<br>Perception of goal attainability	the Work Goal Processes inventory and new items (described later) had to be developed based on scales from existing questionnaires.	-0.18<br>-0.02	0.1<br>0.1	NR<br>NR	Protective<br>Protective	0.01<br>0.0001	Small<br>Small	High
(Poulin & Walter, 1993), USA, social workers, n=879	MBI, NR	Self-esteem<br>Stress<br>Support from supervisor<br>Satisfaction with clients	Self-esteem is measured with a 10-item index (Rosenberg, 1965). The job stress index consists of two items that assess job stress and time pressure on five-point scales. The supervisor support index has five items that rate the respondents' supervisors on four-point scales. Satisfaction with clients is measured with single-item five-point rating scales.	-0.1<br>0.43<br>-0.23<br>-0.14	0.04<br>0.13<br>0.07<br>0.06	0.02<br>0.001<br>NR<br>0.02	NR<br>NR<br>NR<br>NR	NR<br>NR<br>NR<br>NR	Small<br>Medium<br>Small<br>Medium	Low

(Ragsdale & Hoover, 2016), USA, various working populations, n=313	MBI, NR	Cell phone attachment<br>Work related cell phone use during non-work time	Work-related cell phone (WRCP) use (a=0.95) and cell phone attachment (a=0.95) were measured with self-constructed scales including 7 items and 11 items, respectively. Items were assessed on a seven-point scale.	-0.37<br>-0.02	0.14<br>0.02	0.01<br>0.2	Protective*<br>Protective	NR<br>NR	NR<br>NR	High
(Ramarajan et al., 2008), USA, certified nursing assistants, n=108	MBI, NR	Organizational respect	The 5-item scale that was generated directly from a cross-section of organizational members (through the interviews, focus groups, and town hall meetings).	-0.23	0.1	0.02	Protective*	0.11	Small	High
(Richter et al., 2015), Sweden, various working populations, n=3378	MBI-GS, NR	Performance-based self-esteem<br>Work-family conflict	A four-item scale by Hallsten et al. (2005) measured performance-based selfesteem. Work-family conflict was measured with a single item measure ('Do the demands placed on you at work interfere with your home and family life?').	0.04<br>0.02	0.02<br>0.02	0.02<br>0.2	Harmful*<br>Harmful	0.07<br>0.16	Small<br>Medium	Low
(Rubio et al., 2015), Spain, Spanish army subjects, n=242	MBI-GS (The Spamish version), NR	Work-family conflict	A sub-scale of the Occupational Stress Indicator (OSI) byCooper, Sloan, and Williams (1988), translated into Spanish.	0.16	0.07	0.02	Harmful*	0.07	Small	Moderate
(Salanova et al., 2009), Spain, teachers, n=274	MBI-GS, NR	Decision authority<br>Skill discretion<br>Social support<br>Technical obstacles<br>Conflicts and interpersonal problems	A scale was developed for this study	0.01<br>-0.07<br>0.03<br>0.01<br>-0.01	0.04<br>0.07<br>0.05<br>0.04<br>0.04	NR<br>NR<br>NR<br>NR<br>NR	Harmful<br>Protective<br>Harmful<br>Harmful<br>Protective	0.01<br>0.01<br>0.004<br>0.1<br>0.07	Small<br>Small<br>Small<br>Small<br>Small	High

(Spence Laschinger & Finegan, 2008), Canada, nurse managers, n=134	MBI-GS, NR	Effort-reward imbalance<br>Core self-evaluation	The extrinsic effort and the rewards subscale of the Effort Reward Imbalance (ERI) Questionnaire designed to measure components of Siegrist's model. Judge et al.'s (2003) 12-item Core Self-evaluation Scale (CSES).	-0.17<br>0.37	0.08<br>0.1	0.03<br>0.0001	Harmful*<br>Protective*	0.56<br>0.41	Large<br>Large	Low
(Taris et al., 2001), the Netherlands, teachers, n=828	MBI (the Dutch version), NR	Stressful interaction with students<br>Stressful interaction with colleagues	Subscales were taken from Kamphuis and Van Poppel (1994) School Health Questionnaire.	-0.1<br>-0.11	0.04<br>0.04	0.01<br>0.01	Protective*<br>Protective*	0.05<br>0.01	Small<br>Small	Low
(Taris et al., 2004), The Netherlands, teachers, n=920	MBI-ES (Dutch adaptation), NR	Lack of reciprocity from colleagues<br>Lack of reciprocity from students<br>Lack of reciprocity from organization	Two measures were available for each relationship. The first of these was a variation on Hatfield et al.'s (1985) well-used single-item equity measure. The second item was computed as the ratio of the scores on two other items. The first of these tapped the subjective investments in a particular relationship: ''How much do you invest in the work relationship with your students?'' (1:''very little'', 5: 'very much''). The second item measured the perceived benefits of this relationship: ''How much do you receive in return in this relationship?''	-0.16<br>-0.16<br>0.32	0.02<br>0.02<br>0.03	NR<br>NR<br>NR	Protective<br>Protective<br>Harmful	NA<br>NA<br>NA	NA<br>NA<br>NA	Moderate

(Taris et al., 2010), the Netherlands, police officers, n=828	MBI – General Survey, NR	Work and time demands<br>Professional efficacy	Job demands were measured with four items from a Dutch adaptation of the psychological demands scale of the job content questionnaire. Professional efficacy was measured using the six-item professional efficacy scale of the MBI-GS.	0.08<br>-0.06	0.03<br>0.03	0.01<br>0.02	Harmful*<br>Protective*	0.03<br>0.07	Small<br>Small	Low
(Theorell et al., 2012), Sweden, various working populations, n=3285	MBI, NR	Non-listening manager<br>Non listening leadership<br>Self centered leadership	The two selected leadership dimensions were: Does your manager listen to you and pay attention to what you say? In this one-item ‘‘Non-listening leadership’’ variable there were four response categories ranging from ‘‘to a very high extent’’ to ‘‘a very small extent or not at all’’. High score indicates poor condition (non-listening). Range 1–4 with mean 2,15 to 2,20 (2006, 2008 and 2010) and standard deviation 0,7–0,8. ‘‘Self-centred leadership’’: This factor was calculated from three questions (non-participating, asocial and loner) according to results from factor analysis.	-0.43<br>-0.43<br>0.002	0.81<br>0.81<br>0.06	NR<br>NR<br>NR	Protective<br>Protective<br>Protective	NA<br>NA<br>NA	NA<br>NA<br>NA	Low

(Theorell et al., 2013), Sweden, various working populations, n=11525	MBI-GS, ≥75th percentile	Job demands<br>Decision latitude<br>Cultural activity at work	Demand-decision latitude questionnaire<br>Job demands (5 items)<br>Decision latitude (6 items)<br>Cultural activity at work (1 item)	0.14<br>-0.06<br>0.16	0.04<br>0.02<br>0.09	0.0001<br>NR<br>NR	Harmful*<br>Harmful*<br>Harmful	NA<br>NA<br>NA	NA<br>NA<br>NA	Low
(Tonjes & Dickhauser, 2009), Germany, teachers, n=96	MBI the German translation, NR	Performance-avoidance goal<br>Learning goals orientation	Adopted from Dickhäuser et al., 2007 using five-level rating scale from is not true at all (1) to is true exactly (5).	0.16<br>-0.12	0.06<br>0.05	0.01<br>0.02	Harmful*<br>Protective*	0.02<br>0.005	Small<br>Small	High
(Travis et al., 2015), USA, urban public child welfare employees, n=362	MBI, NR	Role ambiguity<br>Role conflict<br>Work-family conflict	Role conflict and role ambiguity were measured using Rizzo,House and Lirtzman's (1970) scale items. Beatty's (1996) three-item work–family conflict scale was used to measure conflict between work and family demands.	0.26<br>0.21<br>0.65	0.16<br>0.1<br>0.2	NR<br>NR<br>NR	Harmful<br>Harmful*<br>Harmful*	0.02<br>0.04<br>0.07	Small<br>Small<br>Small	Moderate
(Turgut et al., 2016), Germany, employees in a company in the automotive industry, n=709	MBI (German version), NR	Organizational support<br>Communication / information flow<br>Resistence to change	Organizational support using five items of a scale by Eisenberger et al. (1986)Informational team climate using three items of the participative safety subscale from the German version (Brodbeck & Maier, 2001) of the team climate inventory (Anderson & West, 1996). Resistance to change: two subscales of the resistance to change scale (Oreg, 2003).	-0.37<br>0.08<br>0.11	0.16<br>0.21<br>0.03	NR<br>NR<br>NR	Protective*<br>Harmful<br>Harmful*	0.04<br>0.02<br>0.21	Small<br>Small<br>Medium	Moderate

(Van de Ven et al., 2013), Belgium, technology employees, n=711	MBI-GS, NR	Emotional demands<br>Emotional support<br>Emotional support seeking	Emotional job demands and emotional job resources were measured using the DISC Questionnaire. Emotional support seeking was measured with five items derived from the Emotional Support-Seeking subscale in the Proactive Coping Inventory.	0.1<br>-0.02<br>0.03	0.04<br>0.02<br>0.02	0.01<br>NR<br>NR	Harmful*<br>Harmful<br>Protective	0.09<br>0.01<br>0.03	Small<br>Small<br>Small	Moderate
(van der Ploeg & Kleber, 2003), the Netherlands, paramedics and drivers (ambulance services), n=123	MBI (the Dutch version), NR	Physical strain<br>Communication / information flow	Physical strain: subscale of the Questionnaire on the Experience and Assessment of Work (QEAW)	0.17<br>0.16	0.07<br>0.07	0.02<br>0.02	Harmful*<br>Harmful*	0.14<br>0.32	Small<br>Medium	Moderate
(Vegchel et al., 2004), Sweden, social insurance organisation employees, n=2255	MBI (the Swedish translation), NR	Work and time demands<br>Emotional demands<br>Job control<br>Social support	Work and time demands: the questions are partly derived from standard measures used in applications of the DC model and the response scale ranged from 1 (always) to 5 (never).<br>Emotional demands. This scale, developed by M. Soderfeldt. The response scale ranged from 1 (not particularly) to 5 (very much).<br>Job control was measured by an eight-item questionnaire with a 5-point response scale ranging from 1 (never) to 5	0.12<br>0.09<br>-0.1<br>-0.07	0.05<br>0.03<br>0.04<br>0.03	0.01<br>0.001<br>0.02<br>NR	Harmful*<br>Harmful*<br>Protective*<br>Protective*	0.51<br>0.16<br>0.11<br>0.19	Large<br>Medium<br>Small<br>Medium	Low

			(very often), based on the work of Harenstam.<br>Social support. Social support was measured with the 7-item questionnaire (5-point response scale ranging from 1 [always] to 5 [never]) from Harenstam.							
(Welp et al., 2016), Switzerland, nurses and physicians, n=493	MBI-HSS (German, French, and Italian translations), NR	Quality of social interactions at work	The three-item nurse-physician relationship scale from the nursing work index revised (NWI-R)	-0.03	0.02	NR	Protective	0.06	Small	Moderate
(Westman et al., 2008), Israel, business travelers, n=66	MBI-GS, NR	Work-family conflict<br>Family-work conflict	Work-family conflict and family-work conflict were measured by eight items developed by Vinokur, Pierce, and Buck (1999).	0.31<br>0.33	0.13<br>0.12	0.02<br>0.005	Harmful*<br>Harmful*	0.1<br>0.3	Small<br>Medium	High
(Wirtz et al., 2017), Germany, leaders and team members of different organizations in banking, auditing, social services, education, and facility management, n=67	MBI, NR	Colleagues/team exhaustion	The three highest loading items of the Maslach Burnout inventory (cf. Kinnunen, Mäkikangas, Mauno, De Cuyper, & De Witte, 2014)	0.11	0.09	0.2	Harmful	0.004	Small	High

(Zwingmann et al., 2016), Germany, services company, n=2324	MBI-GS, NR	Transformational leadership<br>Laissez-faire leadership	Transformational leadership was assessed at time 1 (2010) with five selected items of the German adapted version of the Multifactor Leadership Questionnaire (MLQ 5x; Bass & Avolio, 1995; Felfe & Goihl, 2002). Laissez-faire leadership was measured at time 1 (2010) with one item (MLQ 5x; Bass & Avolio, 1995; Felfe & Goihl, 2002).	0.57<br>0.38	0.24<br>0.16	0.02<br>0.02	Harmful<br>Harmful	0.05<br>0.04	Small<br>Small	Low

LQWQ-N: Leiden Quality of Work Questionnaire for Nurses, DEWSS: Dentists' Experienced Work Stressors Scales, DEJRS: Dentists' Experienced Job Resources Scale, BFQ: Big Five Questionnaire, DCS: Demand-Control-Support, *MBI-GS: Maslach Burnout Inventory general survey, LQWLQ-N: The Leiden Quality of Work Life Questionnaire for nurses, MBI-HSS: Maslach Burnout Inventory Human Service Survey, DC model: Job Demand Control model, OLBI: Oldenburg Burnout Inventory, AWS: Areas of Work life Scale, RESTQ-Work: The Recovery-Stress-Questionnaire for Work, PBSE: Performance-based self-esteem, GO: goal orientation, VC: Value congruency, OBSE: Organizational based self-esteem, BFI: Big Five Inventory, MBI-ES: Maslach Burnout Inventory Educators Survey,* COPSOQ*: The Copenhagen Psychosocial Questionnaire, PSC: Psychosocial safety climate, Practice Environment Scale of the Nursing Work Index PES-NWI, NWI-R: Nursing work index revised, MLQ: Multifactor Leadership Questionnaire, WRCP: Work-related cell phone, ERI: Effort Reward Imbalance, CSES: Core Self-evaluation Scale, NR: not reported, NA: not applicable..*

References

Adriaenssens, J., De Gucht, V., & Maes, S. (2015). Causes and consequences of occupational stress in emergency nurses, a longitudinal study. *J Nurs Manag*, *23*(3), 346-358. https://doi.org/10.1111/jonm.12138

Angelo, R. P., & Chambel, M. J. (2015). The Reciprocal Relationship Between Work Characteristics and Employee Burnout and Engagement: A Longitudinal Study of Firefighters. *Stress and Health*, *31*(2), 106-114. https://doi.org/10.1002/smi.2532

Birkeland, I. K., Richardsen, A. M., & Dysvik, A. (2018). The Role of Passion and Support Perceptions in Changing Burnout: A Johnson-Neyman Approach. *International Journal of Stress Management*, *25*(2), 163-180. https://doi.org/10.1037/str0000057

Boamah, S. A., Read, E. A., & Laschinger, H. K. S. (2017). Factors influencing new graduate nurse burnout development, job satisfaction and patient care quality: a time-lagged study. *J Adv Nurs*, *73*(5), 1182-1195. https://doi.org/10.1111/jan.13215

Childs, J. H., & Stoeber, J. (2012). Do you want me to be perfect? Two longitudinal studies on socially prescribed perfectionism, stress and burnout in the workplace. *Work and Stress*, *26*(4), 347-364. https://doi.org/10.1080/02678373.2012.737547

Chrisopoulos, S., Dollard, M. F., Winefield, A. H., & Dormann, C. (2010). Increasing the probability of finding an interaction in work stress research: A two-wave longitudinal test of the triple-match principle. *Journal of Occupational and Organizational Psychology*, *83*(1), 17-37. https://doi.org/10.1348/096317909x474173

Dahlin, M., Fjell, J., & Runeson, B. (2010). Factors at medical school and work related to exhaustion among physicians in their first postgraduate year. *Nordic Journal of Psychiatry*, *64*(6), 402-408. https://doi.org/10.3109/08039481003759219

Dubois, C. A., Bentein, K., Ben Mansour, J., Gilbert, F., & Bedard, J. L. (2014). Why Some Employees Adopt or Resist Reorganization of Work Practices in Health Care: Associations between Perceived Loss of Resources, Burnout, and Attitudes to Change. *Int J Environ Res Public Health*, *11*(1), 187-201. https://doi.org/10.3390/ijerph110100187

Fernet, C., Gagne, M., & Austin, S. (2010). When does quality of relationships with coworkers predict burnout over time? The moderating role of work motivation. *Journal of Organizational Behavior*, *31*(8), 1163-1180. https://doi.org/10.1002/job.673

Fernet, C., Lavigne, G. L., Vallerand, R. J., & Austin, S. (2014). Fired up with passion: Investigating how job autonomy and passion predict burnout at career start in teachers. *Work and Stress*, *28*(3), 270-288. https://doi.org/10.1080/02678373.2014.935524

Feuerhahn, N., Bellingrath, S., & Kudielka, B. M. (2013). The Interplay of Matching and Non-Matching Job Demands and Resources on Emotional Exhaustion among Teachers. *Applied Psychology-Health and Well Being*, *5*(2), 171-192. https://doi.org/10.1111/aphw.12002

Fida, R., Laschinger, H. K. S., & Leiter, M. P. (2018). The protective role of self-efficacy against workplace incivility and burnout in nursing: A time-lagged study. *Health Care Manage Rev*, *43*(1), 21-29. https://doi.org/10.1097/HMR.0000000000000126

Figueiredo-Ferraz, H., Grau-Alberola, E., Gil-Monte, P. R., & Garcia-Juesas, J. A. (2012). [Burnout and job satisfaction among nursing professionals]. *Psicothema*, *24*(2), 271-276. https://www.ncbi.nlm.nih.gov/pubmed/22420356 (Sindrome de quemarse por el trabajo y satisfaccion laboral en profesionales de enfermeria.)

Firoozabadi, A., Uitdewilligen, S., & Zijlstra, F. R. H. (2018). Should You Switch Off or Stay Engaged? The Consequences of Thinking About Work on the Trajectory of Psychological Well-Being Over Time. *J Occup Health Psychol*, *23*(2), 278-288. https://doi.org/10.1037/ocp0000068

Fong, C. M. (1993). A longitudinal study of the relationships between overload, social support, and burnout among nursing educators. *J Nurs Educ*, *32*(1), 24-29. https://www.ncbi.nlm.nih.gov/pubmed/8380202

Fritz, C., & Sonnentag, S. (2005). Recovery, health, and job performance: effects of weekend experiences. *J Occup Health Psychol*, *10*(3), 187-199. https://doi.org/10.1037/1076-8998.10.3.187

Fritz, C., & Sonnentag, S. (2006). Recovery, well-being, and performance-related outcomes: the role of workload and vacation experiences. *J Appl Psychol*, *91*(4), 936-945. https://doi.org/10.1037/0021-9010.91.4.936

Fusilier, M., & Manning, M. R. (2005). Psychosocial predictors of health status revisited. *J Behav Med*, *28*(4), 347-358. https://doi.org/10.1007/s10865-005-9002-y

Garbarino, S., Cuomo, G., Chiorri, C., & Magnavita, N. (2013). Association of work-related stress with mental health problems in a special police force unit. *BMJ Open*, *3*(7). https://doi.org/10.1136/bmjopen-2013-002791

Gelsema, T. I., van der Doef, M., Maes, S., Janssen, M., Akerboom, S., & Verhoeven, C. (2006). A longitudinal study of job stress in the nursing profession: causes and consequences. *J Nurs Manag*, *14*(4), 289-299. https://doi.org/10.1111/j.1365-2934.2006.00635.x

Gil-Monte, P. R., & Garcia-Juesas, J. A. (2008). Effects of Work Overloading and Self-Efficacy over the Burnout Syndrome: A Longitudinal Survey About Nursing. *Revista Mexicana De Psicologia*, *25*(2), 329-337. <Go to ISI>://WOS:000261236500012

Goddard, R., O'Brien, P., & Goddard, M. (2006). Work environment predictors of beginning teacher burnout. *British Educational Research Journal*, *32*(6), 857-874. https://doi.org/10.1080/01411920600989511

Gonzalez-Morales, M. G., Peiro, J. M., Rodriguez, I., & Bliese, P. D. (2012). Perceived collective burnout: a multilevel explanation of burnout. *Anxiety Stress and Coping*, *25*(1), 43-61. https://doi.org/10.1080/10615806.2010.542808

Gonzalez-Morales, M. G., Rodriguez, I., & Peiro, J. M. (2010). A Longitudinal Study of Coping and Gender in a Female-Dominated Occupation: Predicting Teachers' Burnout. *J Occup Health Psychol*, *15*(1), 29-44. https://doi.org/10.1037/a0018232

Gregersen, S., Vincent-Höper, S., & Nienhaus, A. (2014). The Relation Between Leadership and Perceived Well-Being: What Role Does Occupational Self-Efficacy Play? *Journal of Leadership Studies*, *8*. https://doi.org/10.1002/jls.21318

Gregory, S. T., & Menser, T. (2015). Burnout Among Primary Care Physicians: A Test of the Areas of Worklife Model. *J Healthc Manag*, *60*(2), 133-148.

Hakanen, J., Schaufeli, W., & Ahola, K. (2008). The Job Demands-Resources Model: A three-year cross-lagged study of burnout, depression, commitment, and work engagement. *Work and Stress*, *22*. https://doi.org/10.1080/02678370802379432

Hertzberg, T. K., Ro, K. I., Vaglum, P. J. W., Moum, T., Rovik, J. O., Gude, T., Ekeberg, O., & Tyssen, R. (2016). Work-home interface stress: an important predictor of emotional exhaustion 15 years into a medical career. *Ind Health*, *54*(2), 139-148. https://doi.org/DOI 10.2486/indhealth.2015-0134

Hochwalder, J. (2008). A longitudinal study of the relationship between empowerment and burnout among registered and assistant nurses. *Work-a Journal of Prevention Assessment & Rehabilitation*, *30*(4), 343-352. <Go to ISI>://WOS:000258695100002

Hornung, S., Weigl, M., Glaser, J., & Angerer, P. (2013). Is It So Bad or Am I So Tired? Cross-Lagged Relationships Between Job Stressors and Emotional Exhaustion of Hospital Physicians. *Journal of Personnel Psychology*, *12*(3), 124-131. https://doi.org/10.1027/1866-5888/a000088

Huang, Y. H., Chen, C. H., Du, P. L., & Huang, I. C. (2012). The causal relationships between job characteristics, burnout, and psychological health: a two-wave panel study. *International Journal of Human Resource Management*, *23*(10), 2108-2125. https://doi.org/10.1080/09585192.2011.610934

Hudek-Knezevic, J., Maglica, B. K., & Krapic, N. (2011). Personality, organizational stress, and attitudes toward work as prospective predictors of professional burnout in hospital nurses. *Croat Med J*, *52*(4), 538-549. https://doi.org/10.3325/cmj.2011.52.538

Idris, M. A., Dollard, M. F., & Yulita. (2014). Psychosocial safety climate, emotional demands, burnout, and depression: a longitudinal multilevel study in the Malaysian private sector. *J Occup Health Psychol*, *19*(3), 291-302. https://doi.org/10.1037/a0036599

Innstrand, S. T., Langballe, E., Espnes, G., Falkum, & Aasland. (2008). Positive and negative work-family interaction and burnout: A longitudinal study of reciprocal relations. *Work and Stress*, 1-15. https://doi.org/10.1080/02678370801975842

Innstrand, S. T., Langballe, E., & Falkum, E. (2011). The longitudinal effects of individual vulnerability, organisational factors, and work–home interaction on burnout among male church ministers in Norway. *Mental Health, Religion & Culture*, 241-257. https://doi.org/10.1080/13674670903470621

Jensen, M., & Knudsen, K. (2016). A two-wave cross-lagged study of business travel, work–family conflict, emotional exhaustion, and psychological health complaints. *European Journal of Work and Organizational Psychology*, *26*, 1-12. https://doi.org/10.1080/1359432X.2016.1197206

Jimenez, P., & Dunkl, A. (2017). The Buffering Effect of Workplace Resources on the Relationship between the Areas of Worklife and Burnout. *Frontiers in Psychology*, *8*. https://doi.org/ARTN 1210.3389/fpsyg.2017.00012

Konze, A.-K., Rivkin, W., & Schmidt, K.-H. (2017). Is Job Control a Double-Edged Sword? A Cross-Lagged Panel Study on the Interplay of QuantitativeWorkload, Emotional Dissonance, and Job Control on Emotional Exhaustion. *Int J Environ Res Public Health*, *14*. https://doi.org/10.3390/ijerph14121608

Korunka, C., Kubicek, B., Paskvan, M., & Ulferts, H. (2015). Changes in work intensification and intensified learning: challenge or hindrance demands? *Journal of Managerial Psychology*, *30*(7), 786-800. https://doi.org/10.1108/Jmp-02-2013-0065

Kubicek, B., & Korunka, C. (2015). Does job complexity mitigate the negative effect of emotion-rule dissonance on employee burnout? *Work and Stress*, *29*(4), 379-400. https://doi.org/10.1080/02678373.2015.1074954

Kubicek, B., Korunka, C., & Tement, S. (2014). Too much job control? Two studies on curvilinear relations between job control and eldercare workers' well-being. *Int J Nurs Stud*, *51*. https://doi.org/10.1016/j.ijnurstu.2014.05.005

Kutney-Lee, A., Wu, E. S., Sloane, D. M., & Aiken, L. H. (2013). Changes in hospital nurse work environments and nurse job outcomes: an analysis of panel data. *Int J Nurs Stud*, *50*(2), 195-201. https://doi.org/10.1016/j.ijnurstu.2012.07.014

Langballe, E., Innstrand, S. T., Aasland, O., & Falkum, E. (2011). The Predictive Value of Individual Factors, Work-Related Factors, and Work-Home Interaction on Burnout in Female and Male Physicians: A Longitudinal Study. *Stress and Health*, *27*, 73-87. https://doi.org/10.1002/smi.1321

Lapointe, É., Vandenberghe, C., & Boudrias, J.-S. (2013). Psychological contract breach, affective commitment to organization and supervisor, and newcomer adjustment: A three-wave moderated mediation model. *Journal of Vocational Behavior*, *83*, 528–538. https://doi.org/10.1016/j.jvb.2013.07.008

Laugaa, D., & Bruchon-Schweitzer, M. (2005). L'ajustement au stress professionnel chez les enseignants français du premier degré. *L'Orientation scolaire et professionnelle*. https://doi.org/10.4000/osp.428

Laugaa, D., Rascle, N., & Bruchon-Schweitzer, M. (2008). Stress and burnout among French elementary school teachers: A transactional approach. *European Review of Applied Psychology-Revue Europeenne De Psychologie Appliquee*, *58*(4), 241-251. https://doi.org/10.1016/j.erap.2008.09.007

Lavigne, G. L., Forest, J., & Crevier-Braud, L. (2012). Passion at work and burnout: A two-study test of the mediating role of flow experiences. *European Journal of Work and Organizational Psychology*, *21*(4), 518-546. https://doi.org/10.1080/1359432x.2011.578390

Leiter, M. P., Hakanen, J. J., Ahola, K., Toppinen-Tanner, S., Koskinen, A., & Vaananen, A. (2013). Organizational predictors and health consequences of changes in burnout: A 12-year cohort study. *Journal of Organizational Behavior*, *34*(7), 959-973. https://doi.org/10.1002/job.1830

Lindeman, B., Petrusa, E., McKinley, S., Hashimoto, D. A., Gee, D., Smink, D. S., Mullen, J. T., & Phitayakorn, R. (2017). Association of Burnout With Emotional Intelligence and Personality in Surgical Residents: Can We Predict Who Is Most at Risk? *J Surg Educ*, *74*(6), e22-e30. https://doi.org/10.1016/j.jsurg.2017.11.001

Lizano, E. L., & Barak, M. E. M. (2012). Workplace demands and resources as antecedents of job burnout among public child welfare workers: A longitudinal study. *Children and Youth Services Review*, *34*(9), 1769-1776. https://doi.org/10.1016/j.childyouth.2012.02.006

Lizano, E. L., & Barak, M. M. (2015). Job burnout and affective wellbeing: A longitudinal study of burnout and job satisfaction among public child welfare workers. *Children and Youth Services Review*, *55*, 18-28. https://doi.org/10.1016/j.childyouth.2015.05.005

Lorente Prieto, L., Salanova Soria, M., Martinez Martinez, I., & Schaufeli, W. (2008). Extension of the Job Demands-Resources model in the prediction of burnout and engagement among teachers over time. *Psicothema*, *20*(3), 354-360. https://www.ncbi.nlm.nih.gov/pubmed/18674427

Lu, L., Lin, H. Y., & Cooper, C. L. (2013). Unhealthy and present: motives and consequences of the act of presenteeism among Taiwanese employees. *J Occup Health Psychol*, *18*(4), 406-416. https://doi.org/10.1037/a0034331

Makikangas, A., & Kinnunen, U. (2003). Psychosocial work stressors and well-being: self-esteem and optimism as moderators in a one-year longitudinal sample. *Personality and Individual Differences*, *35*(3), 537-557. https://doi.org/Pii S0191-8869(02)00217-9. Doi 10.1016/S0191-8869(02)00217-9

Martinez-Inigo, D., & Totterdell, P. (2016). The mediating role of distributive justice perceptions in the relationship between emotion regulation and emotional exhaustion in healthcare workers. *Work and Stress*, *30*(1), 26-45. https://doi.org/10.1080/02678373.2015.1126768

Mauno, S., De Cuyper, N., Kinnunen, U., Ruokolainen, M., Rantanen, J., & Makikangas, A. (2015). The prospective effects of work-family conflict and enrichment on job exhaustion and turnover intentions: comparing long-term temporary vs. permanent workers across three waves. *Work and Stress*, *29*(1), 75-94. https://doi.org/10.1080/02678373.2014.1003997

McManus, I. C., Winder, B. C., & Gordon, D. (2002). The causal links between stress and burnout in a longitudinal study of UK doctors. *Lancet*, *359*(9323), 2089-2090. https://doi.org/10.1016/s0140-6736(02)08915-8

Meier, L. L., Tschudi, P., Meier, C. A., Dvorak, C., & Zeller, A. (2015). When general practitioners don't feel appreciated by their patients: prospective effects on well-being and work-family conflict in a Swiss longitudinal study. *Fam Pract*, *32*(2), 181-186. https://doi.org/10.1093/fampra/cmu079

Park, S. G., Chang, B. H., Mueller, L., Resnick, S. G., & Eisen, S. V. (2016). Predictors of Employment Burnout Among VHA Peer Support Specialists. *Psychiatric Services*, *67*(10), 1108-1114. https://doi.org/10.1176/appi.ps.201500239

Petrou, P., Demerouti, E., & Schaufeli, W. B. (2015). Job crafting in changing organizations: Antecedents and implications for exhaustion and performance. *J Occup Health Psychol*, *20*(4), 470-480. https://doi.org/10.1037/a0039003

Philipp, A., & Schupbach, H. (2010). Longitudinal effects of emotional labour on emotional exhaustion and dedication of teachers. *J Occup Health Psychol*, *15*(4), 494-504. https://doi.org/10.1037/a0021046

Pisanti, R., van der Doef, M., Maes, S., Meier, L. L., Lazzari, D., & Violani, C. (2016). How Changes in Psychosocial Job Characteristics Impact Burnout in Nurses: A Longitudinal Analysis. *Frontiers in Psychology*, *7*. https://doi.org/ARTN 108210.3359/fpsyg.2016.01082

Pomaki, G., Karoly, P., & Maes, S. (2009). Linking Goal Progress to Subjective Well-Being at Work: The Moderating Role of Goal-Related Self-Efficacy and Attainability. *J Occup Health Psychol*, *14*(2), 206-218. https://doi.org/10.1037/a0014605

Poulin, J., & Walter, C. (1993). Social-Worker Burnout - a Longitudinal-Study. *Social Work Research & Abstracts*, *29*(4), 5-11. https://doi.org/DOI 10.1093/swra/29.4.5

Ragsdale, J. M., & Hoover, C. S. (2016). Cell phones during nonwork time: A source of job demands and resources. *Computers in Human Behavior*, *57*, 54-60. https://doi.org/10.1016/j.chb.2015.12.017

Ramarajan, L., Barsade, S., & Burack, O. (2008). The influence of organizational respect on emotional exhaustion in the human services. *The Journal of Positive Psychology*, *3*, 4-18. https://doi.org/10.1080/17439760701750980

Richter, A., Schraml, K., & Leineweber, C. (2015). Work-family conflict, emotional exhaustion and performance-based self-esteem: reciprocal relationships. *Int Arch Occup Environ Health*, *88*(1), 103-112. https://doi.org/10.1007/s00420-014-0941-x

Rubio, C., Osca, A., Recio, P., Urien, B., & Peiro, J. M. (2015). Work-family conflict, self-efficacy, and emotional exhaustion: A test of longitudinal effects. *Journal of Work and Organizational Psychology-Revista De Psicologia Del Trabajo Y De Las Organizaciones*, *31*(3), 147-154. https://doi.org/10.1016/j.rpto.2015.06.004

Salanova, M., Schaufeli, W., Martínez, I., & Bresó, E. (2009). How obstacles and facilitators predict academic performance: The mediating role of study burnout and engagement. *Anxiety Stress Coping*, *23*, 53-70. https://doi.org/10.1080/10615800802609965

Spence Laschinger, H. K., & Finegan, J. (2008). Situational and dispositional predictors of nurse manager burnout: a time-lagged analysis. *J Nurs Manag*, *16*(5), 601-607. https://doi.org/10.1111/j.1365-2834.2008.00904.x

Taris, T. W., Kompier, M. A. J., Geurts, S. A. E., Houtman, I. L. D., & van den Heuvel, F. F. M. (2010). Professional efficacy, exhaustion, and work characteristics among police officers: A longitudinal test of the learning-related predictions of the demand-control model. *Journal of Occupational and Organizational Psychology*, *83*(2), 455-474. https://doi.org/10.1348/096317909x424583

Taris, T. W., Peeters, M. C., Le Blanc, P. M., Schreurs, P. J., & Schaufeli, W. B. (2001). From inequity to burnout: the role of job stress. *J Occup Health Psychol*, *6*(4), 303-323. https://www.ncbi.nlm.nih.gov/pubmed/11605825

Taris, T. W., Van Horn, J. E., Schaufeli, W. B., & Schreurs, P. J. G. (2004). Inequity, burnout and psychological withdrawal among teachers: A dynamic exchange model. *Anxiety Stress and Coping*, *17*(1), 103-122. https://doi.org/10.1080/1061580031000151620

Theorell, T., Nyberg, A., Leineweber, C., Magnusson Hanson, L. L., Oxenstierna, G., & Westerlund, H. (2012). Non-listening and self centered leadership--relationships to socioeconomic conditions and employee mental health. *PLoS One*, *7*(9), e44119. https://doi.org/10.1371/journal.pone.0044119

Theorell, T., Osika, W., Leineweber, C., Hanson, L. L. M., Horwitz, E. B., & Westerlund, H. (2013). Is cultural activity at work related to mental health in employees? *Int Arch Occup Environ Health*, *86*(3), 281-288. https://doi.org/10.1007/s00420-012-0762-8

Tonjes, B., & Dickhauser, O. (2009). Longitudinal effects of goal orientation on factors of occupational burden in the teacher profession. *Zeitschrift Fur Entwicklungspsychologie Und Padagogische Psychologie*, *41*(2), 79-86. https://doi.org/10.1026/0049-8637.41.2.79

Travis, D., Lizano, E., & Mor Barak, M. (2015). 'I'm So Stressed!': A Longitudinal Model of Stress, Burnout and Engagement among Social Workers in Child Welfare Settings. *British Journal of Social Work*, *46*. https://doi.org/10.1093/bjsw/bct205

Turgut, S., Michel, A., Rothenhofer, L. M., & Sonntag, K. (2016). Dispositional resistance to change and emotional exhaustion: moderating effects at the work-unit level. *European Journal of Work and Organizational Psychology*, *25*(5), 735-750. https://doi.org/10.1080/1359432x.2016.1157583

Van de Ven, B., van den Tooren, M., & Vlerick, P. (2013). Emotional Job Resources and Emotional Support Seeking as Moderators of the Relation Between Emotional Job Demands and Emotional Exhaustion: A Two-Wave Panel Study. *J Occup Health Psychol*, *18*(1), 1-8. https://doi.org/10.1037/a0030656

van der Ploeg, E., & Kleber, R. J. (2003). Acute and chronic job stressors among ambulance personnel: predictors of health symptoms. *Occup Environ Med*, *60*, 40-46. <Go to ISI>://WOS:000183251600008

Vegchel, N. v., Jonge, J. d., Söderfeldt, M., Dormann, C., & Schaufeli, W. (2004). Quantitative Versus Emotional Demands Among Swedish Human Service Employees: Moderating Effects of Job Control and Social Support. *International Journal of Stress Management*, *11*(1), 21-40. https://doi.org/10.1037/1072-5245.11.1.21

Welp, A., Meier, L., & Manser, T. (2016). The interplay between teamwork, clinicians' emotional exhaustion, and clinician-rated patient safety: A longitudinal study. *Critical Care*, *20*. https://doi.org/10.1186/s13054-016-1282-9

Westman, M., Etzion, D., & Gattenio, E. (2008). International business travels and the work-family interface: A longitudinal study. *Journal of Occupational and Organizational Psychology*, *81*, 459-480. https://doi.org/10.1348/096317908X310265

Wirtz, N., Rigotti, T., Otto, K., & Loeb, C. (2017). What About the Leader? Crossover of Emotional Exhaustion and Work Engagement From Followers to Leaders. *J Occup Health Psychol*, *22*(1), 86-97. https://doi.org/10.1037/ocp0000024

Zwingmann, I., Wolf, S., & Richter, P. (2016). Every light has its shadow: a longitudinal study of transformational leadership and leaders' emotional exhaustion. *Journal of Applied Social Psychology*, *46*(1), 19-33. https://doi.org/10.1111/jasp.12352
