# Supplementary material for: Predictors of Occupational Burnout: A Systematic Review
Source: Int J Environ Res Public Health. 2021 Aug 31;18(17):9188. doi: 10.3390/ijerph18179188 (PMC8430894; doi:10.3390/ijerph18179188)
Supplement: Supplementary file 1 [file ijerph-18-09188-s001.zip › Table S2.pdf]

## Situational characteristics

### Job demands

| Article | Factor's original name | Included in | Sample size | Z-score |
|---|---|---|---|---|
| Chrisopoulos_2010 | cognitive demands | cognitive demands | 179 | 1.250 |
| Korunka_2015 | intensified learning demands | cognitive demands | 587 | -3.289 |
| Kubicek_2015 | job complexity | cognitive demands | 591 | -0.429 |
| Borritz_2005 (CBI) | emotional demands | emotional demands | 952 | -0.470 |
| Borritz_2005 (CBI) | demands for hiding emotions | emotional demands | 952 | 0.600 |
| Chrisopoulos_2010 | emotional demands | emotional demands | 179 | 0.429 |
| Feuerhahn_2013_2 | emotional job demands | emotional demands | 87 | 0.118 |
| Hertzberg_2016 | emotional demands | emotional demands | 274 | 1.316 |
| Idris_2014_1 | emotional demands | emotional demands | 117 | 2.125 |
| Konze_2017 | emotional dissonance | emotional demands | 139 | 3.600 |
| Kubicek_2015 | emotional rule dissonance | emotional demands | 591 | 1.750 |
| Lorente Prieto_2008 | job demands ( emotional demands) | emotional demands | 274 | 2.667 |
| Van_de_ven_2013 | emotional job demands | emotional demands | 4622 | 2.564 |
| Vegchel_2004 | emotional demand | emotional demands | 2255 | 3.333 |
| Adriaenssens_2015 | job demands | job demands | 170 | 2.326 |
| Garbarino_2013 | job demand | job demands | 289 | 2.326 |
| Hakanen_2008 | job demands | job demands | 3035 | 3.286 |
| Hudek-Knezevic_2011_1 | job demand (quantitative overload + role confllict) | job demands | 118 | 3.884 |
| Laugaa_2008 | job demands (quantitative and qualitative) | job demands | 259 | 2.576 |
| Theorell_2013 | psychological demands | job demands | 11525 | 3.891 |
| Chrisopoulos_2010 | physical demands | physical demands | 179 | 0.571 |
| Gelsema_2006 | physical demands | physical demands | 381 | 2.576 |
| Borritz_2005 (CBI) | quantitative demands | quantitative demands | 952 | -0.113 |
| Borritz_2005 (CBI) | work pace | quantitative demands | 952 | 0.912 |
| Dahlin_2010 (OLBI) | weekly working hours | quantitative demands | 186 | 1.600 |
| Feuerhahn_2013_2 | time pressure | quantitative demands | 87 | -0.231 |
| Fong_1993 | job demands (quantitative) | quantitative demands | 84 | 2.321 |
| Fritz_2006 OLBI | workload after vacation | quantitative demands | 221 | 2.571 |
| Garbarino_2013 | Effort | quantitative demands | 289 | 2.581 |
| Gelsema_2006 | work and time demands | quantitative demands | 381 | 1.282 |
| Gil-Monte_2008 | work overload | quantitative demands | 316 | 2.308 |
| Goddard_2006 | work pressure | quantitative demands | 79 | 3.295 |
| Gregory_2015 | workload | quantitative demands | 153 | 3.888 |
| Hertzberg_2016 | time pressure | quantitative demands | 274 | 1.690 |
| Hertzberg_2016 | working hours per week | quantitative demands | 274 | 0.845 |
| Hornung_2013 | work overload | quantitative demands | 95 | -0.543 |
| Huang_2012 | job demands ( work fast and hard, great deal to do, too little time ) | quantitative demands | 299 | 1.297 |
| Instrand_2011_1 | workload | quantitative demands | 308 | -1.277 |
| Jimenez_2017_1 | workload | quantitative demands | 141 | 2.073 |
| Konze_2017 | quantitative workload (time pressure and work volume) | quantitative demands | 139 | 1.400 |
| Korunka_2015 | work intensification demands | quantitative demands | 587 | 3.301 |
| Lorente Prieto_2008 | role overload | quantitative demands | 274 | 5.400 |
| Park_2016 | hours of direct services | quantitative demands | 152 | 1.640 |
| Pisanti_2016 | job demands ( work and time pressure) | quantitative demands | 217 | -1.290 |
| Taris_2010 | work overload/time pressure | quantitative demands | 828 | 2.581 |
| Vegchel_2004 | quantitative demands | quantitative demands | 2255 | 2.553 |

# Decision Latitude (control)

| Article | Factor's original name | Included in | Sample size | Z-score |
|---|---|---|---|---|
| Dubois_2014 | autonomy | autonomy | 96 | -2.3225806 |
| Instrand_2011_1 | autonomy | autonomy | 308 | 2.5806452 |
| Leiter_2013 | decision authority | decision auhtority | 4396 | -1.25 |
| Borritz_2004 | influence | decision authority | 952 | -1.5973856 |
| Dubois_2014 | informal power | decision authority | 96 | -3.8905919 |
| Gelsema_2006 | decision authority | decision authority | 381 | -1.2857143 |
| Salanova_2005 | Indiscipline managment ( defined as the chance of admonishing problematic students) | decision authority | 274 | 0.25 |
| Adriaenssens_2015 | decision lattitiude | decision latitude | 170 | 0.3333333 |
| Garbarino_2013 | decision lattitude | decision latitude | 289 | -1.2857143 |
| Huang_2012 | job control | decision latitude | 299 | -1.2888889 |
| Jimenez_2017_1 | control | decision latitude | 141 | -0.2173913 |
| Konze_2017 | job control | decision latitude | 139 | 2.8 |
| Kubicek_2014 | job control | decision latitude | 591 | -1.2727273 |
| Pisanti_2015 | decision lattitiude | decision latitude | 217 | -1.2903226 |
| Theorell_2013 | decision lattitude | decision latitude | 11525 | -3 |
| Vegchel_2004 | job control | decision latitude | 2255 | -2.3255814 |
| Borritz_2004 | possibilities for development | skill discretion | 952 | -1.4740061 |
| Dubois_2014 | opportunities for stimulating work | skill discretion | 96 | -2.3300971 |
| Gelsema_2006 | skill discretion | skill discretion | 381 | 1.2857143 |
| Leiter_2013 | skill discretion | skill discretion | 4396 | 1.25 |
| Salanova_2005 | cLass managment (chance of changing type or dynamics of the activities) | skill discretion | 274 | -1 |

# Job resources

| Article | Factor's original name | Included in | Sample size | Z-score |
|---|---|---|---|---|
| Boamah_2017 | short-staffing | lack of resources | 405 | 2.5758293 |
| Meier_2015 | lack of reward | lack of resources | 246 | 2.3867077 |
| Salanova_2005 | technical obstacles | lack of resources | 274 | 0.25 |
| Spence-Laschinger_2008 | Effort-reward imbalance | lack of resources | 134 | 3.8905919 |
| Adriaenssens_2015 | material resources | resources | 170 | -0.25 |
| Adriaenssens_2015 | personnel resources | resources | 170 | -0.25 |
| Adriaenssens_2015 | reward | resources | 170 | 1.6666667 |
| Borritz_2005 | meaning of work | resources | 952 | 1.2772544 |
| Chrisopoulos_2010 | cognitive ressources | resources | 179 | -0.2222222 |
| Chrisopoulos_2010 | physical ressources | resources | 179 | 0.5 |
| Dahlin_2010 | learning climate | resources | 186 | -2.326348 |
| Garbarino_2013 | reward | resources | 289 | -2.5758293 |
| Gelsema_2006 | material ressources | resources | 381 | -1.2815516 |
| Gelsema_2006 | personnel resources | resources | 381 | 1.2815516 |
| Gelsema_2006 | reward | resources | 381 | 1.2815518 |
| Goddard_2006 | worplace innovation | resources | 79 | -3.8905919 |
| Gonzales-Morales_2010 | quality of work place facilities | resources | 555 | -1.6759777 |
| Gonzales-Morales_2010 | teacher-students ratio | resources | 555 | -0.4285714 |
| Hakanen_2008 | job ressources | resources | 3035 | -2.3263479 |
| Jimenez_2017_1 | job ressources | resources | 141 | -0.2211187 |
| Jimenez_2017_1 | reward | resources | 141 | -1.2815516 |
| Kutney-Lee_2013 | staffing (number of patients nurses cared for) | resources | 5957 | -2.1700904 |
| Theorell_2013 | cultural activity at work | resources | 11525 | -2 |

# Social interactions at work

| Article | Factor's original name | Included in | Sample size | Z-score |
|---|---|---|---|---|
| Chrisopoulos_2010 | emotional ressources (emotional support) | emotional support | 179 | -1.1111111 |
| Feuerhahn_2013 | emotional ressources (emotional support) | emotional support | 56 | -1.5 |
| Van_de_ven_2013 | emotional job ressources | emotional support | 711 | 1.5 |
| Fernet_2010 | quality of relationships with coworkers | good interpersonal relations | 276 | -2.5758293 |
| Gelsema_2006 | nurse-doctor collaboration | good interpersonal relations | 381 | 1.2815516 |
| Jimenez_2017_1 | community | good interpersonal relations | 141 | 0.3120533 |
| Jimenez_2017_1 | fairness (captures the extent to which decisions and resources allocation at work are perceived as fair and equitable) | good interpersonal relations | 141 | -0.6666433 |
| Ramarajan_2008 | organizational respect | good interpersonal relations | 108 | -2.3263479 |
| Welp_2016 | interpersonal teamwork -> quality of relationships at work | good interpersonal relations | 493 | -1.5 |
| Adriaenssens_2015 | social harassment | social hindrance | 170 | -1.6666667 |
| Angelo_2015 | organizational demands >> conflict and interpersonal problems | social hindrance | 1610 | 2.3263479 |
| Feuerhahn_2013 | classroom disruption | social hindrance | 56 | 0.0769231 |
| Feuerhahn_2013 | parents' criticism | social hindrance | 56 | 2.1111111 |
| Feuerhahn_2013 | conflicts with colleagues | social hindrance | 56 | 1.5 |
| Fida_2018 | work incivilitiy from supervisor | social hindrance | 596 | 1.2815516 |
| Fida_2018 | work incivilitiy from collegues | social hindrance | 596 | 2.3263479 |
| Fida_2018 | work incivilitiy from physician | social hindrance | 596 | 2.5758293 |
| Lapointe_2013 | psychological contract breach (employees' perception that the organization has failed to meet obligations) | social hindrance | 224 | 1.2815516 |
| Laugaa_2008 | conflicts and interperosonal problems | social hindrance | 259 | 2.5758293 |
| Salanova_2005 | parents/students obstacles | social hindrance | 274 | -0.25 |
| Adriaenssens_2015 | social support | social support | 170 | -3.5 |
| Birkeland_2018 | perceived supervision support | social support | 1263 | -3 |
| Birkeland_2018 | perceived coworkers support | social support | 1263 | -2 |
| Borritz_2005 | interpersonal relations and leadership (social support) | social support | 952 | 0.9223141 |
| Dubois_2014 | Ressource loss ( supervisor support) | social support | 96 | -1.0833333 |
| Dubois_2014 | support from colleagues (group cohesion) | social support | 96 | -2.3188406 |
| Fusilier_2005 | social support | social support | 260 | -1.2815516 |
| Garbarino_2013 | occuparional stress variables (support) | social support | 289 | -1.2941176 |
| Gelsema_2006 | social support supervisor | social support | 381 | 1.3333333 |
| Gelsema_2006 | social support collegues | social support | 381 | 1.3333333 |
| Hertzberg_2016 | support from partner | social support | 274 | -0.1219512 |
| Hertzberg_2016 | support from colleagues | social support | 274 | -1.5 |
| Lizano_2012 | supervisory support | social support | 335 | -1.7142857 |
| Lizano_2012 | organizational support | social support | 335 | 1.5 |
| Pisanti_2015 | social support | social support | 217 | 3.1428571 |
| Poulin_1993 | organisational variables (supervisor support) | social support | 879 | -3.2857143 |
| Salanova_2005 | social facilitators | social support | 274 | 0.6 |
| Turgut_2016 | perceived organizational support | social support | 709 | -2.3125 |
| Vegchel_2004 | job ressources ( social support) | social support | 2255 | -2.3333333 |

# Communication & Leadership

| Article | Factor's original name | Included in | Sample size | Z-score |
|---|---|---|---|---|
| Borritz_2005 | role conflict | conflicting/ poor communication | 952 | 0.905 |
| Lizano_2015 | role ambiguity | conflicting/ poor communication | 361 | 2.576 |
| Travis_2016 | role ambiguity | conflicting/ poor communication | 362 | 1.625 |
| Travis_2016 | role conflict | conflicting/ poor communication | 362 | 2.100 |
| Van_der_Ploeg_2003 | poor communication | conflicting/ poor communication | 123 | 2.326 |
| Borritz_2005 | quality of leadership | enriching leadership | 952 | 1.726 |
| Gregersen_2014 | transformational leadership | enriching leadership | 339 | -1.282 |
| Idris_2014_1 | psychosocial safety climate (management commitment, organzational communication) | enriching leadership | 117 | -2.326 |
| Zwingmann_2016 | transformational leadership (leader encourage, inspire and motivate employees) | enriching leadership | 2324 | 2.326 |
| Zwingmann_2016 | laissez-faire leadership ("guided liberty" where employees get all necessary skills to get to their goal) | enriching leadership | 2324 | 2.326 |
| Adriaenssens_2015 | work agreements | informational team climate | 170 | 0.250 |
| Borritz_2005 | role clarity | informational team climate | 952 | -1.264 |
| Borritz_2005 | predictability | informational team climate | 952 | -2.047 |
| Gelsema_2006 | work agreements | informational team climate | 381 | -1.282 |
| Gelsema_2006 | communication | informational team climate | 381 | -1.282 |
| Leiter_2013 | information flow ( amount and quality of information to which respondents had access) | informational team climate | 4356 | -2.576 |
| Leiter_2013 | predictability | informational team climate | 4356 | -1.282 |
| Turgut_2016 | informational team climate | informational team climate | 709 | 0.381 |
| Theorell_2012 | non-listening leadership (Does your manager listen to you and pay attention to what you say? ) | non-collaborative leadership | 3285 | -0.529 |
| Theorell_2012 | self centered leadership ( calculated from three questions (non-participating, asocial and loner) | non-collaborative leadership | 3285 | 0.033 |
| Theorell_2013 | non-listening manager | non-collaborative leadership | 8315 | 1.625 |

# Individual characteristics

## Personality, Coping & Self evaluation

| Article | Factor's original name | Included in | Sample size | Z-score |
|---|---|---|---|---|
| Laugaa_2008 | coping centered on the problem | adaptative coping | 259 | -2.575829 |
| Laungaa_2009 | problem oriented coping | adaptative coping | 410 | -2.326348 |
| Van_de_ven_2013 | emotional support seeking | adaptative coping | 711 | -1.281552 |
| Firoozabadi_2018 (OLBI) | problem solving | adaptative coping | 123 | -2.326348 |
| Philipp_2010 | deep acting (change of the inner emotional state - regulating feelings) | adaptative coping | 102 | -2.575829 |
| Martinez-inigo_2016 | deep acting | adaptative coping | 233 | -0.125 |
| Laugaa_2008 | traditional teaching coping | maladaptative coping | 259 | 2.575829 |
| Firoozabadi_2018 (OLBI) | affective rumination | maladaptative coping | 123 | 2.326348 |
| Philipp_2010 | surface acting (superficial expression of an emotion which is not actually felt - regulating expressions) | maladaptative coping | 102 | -1.281552 |
| Martinez-inigo_2016 | surface acting | maladaptative coping | 233 | 0.333333 |
| Feuerhahn_2013_2 | self efficacy | self efficacy | 87 | -3 |
| Taris_2010 | professional efficacy | self efficacy | 828 | -2.326348 |
| Gregersen_2014 | occupational self efficacy | self efficacy | 339 | -1.281552 |
| Fida_2018 | relational self-efficacy | self efficacy | 596 | -2.575829 |
| Gil-Monte_2008 | self efficacy | self efficacy | 316 | -1.281552 |
| Laugaa_2008 | self efficacy | self efficacy | 259 | -2.575829 |
| Park, 2016 | general self efficacy | self efficacy | 156 | 1.285714 |
| Pomaki, 2009 | goal self efficacy | self efficacy | 222 | -0.2 |
| Hochwälder_2008 | psychological empowerment (meaning, competence , self determination, impact) | self efficacy | 838 | 2.575829 |
| Spence-Laschinger_2008 | core self evaluation | self esteem | 134 | -2.17009 |
| Instrand_2011 OLBI | job performance-based self-esteem | self esteem | 308 | -1.281552 |
| Poulin_1993 | personnal variables ( self esteem) | self-esteem | 879 | -2.326348 |
| Lapointe_2011 | OBSE : organizational based self esteem | self-esteem | 224 | -1.281552 |
| Dahlin_2010 | Performance-based self-esteem | self-esteem | 186 | 1.281552 |
| Richter_2015 | performance-based self-esteem | self-esteem | 3378 | 2.326348 |
| Fusilier_2005 | type A behavior (excessive competitiveness and aggression and a fast-paced life style) | unvalued traits/characteristics | 260 | 2.575829 |
| Hudek-Knezevic_2011 | neurotcism | unvalued traits/characteristics | 118 | 0.622 |
| Hudek-Knezevic_2011 | extraversion | valued traits/characteristics | 118 | 1.680 |
| Hudek-Knezevic_2011 | agreeableness | valued traits/characteristics | 118 | -0.622 |
| Hudek-Knezevic_2011 | conscientiousness | valued traits/characteristics | 118 | 0.633 |
| Hudek-Knezevic_2011 | openess to experience | valued traits/characteristics | 118 | 0.516 |
| Fusilier_2005 | hardiness (perception of events as opportunities and challenges rather than stressors) | valued traits/characteristics | 260 | -1.281552 |
| Garbarino_2013 | extraversion | valued traits/characteristics | 289 | -1.282 |
| Garbarino_2013 | agreeableness | valued traits/characteristics | 289 | -1.282 |
| Garbarino_2013 | consciouentiousness | valued traits/characteristics | 289 | -1.282 |
| Garbarino_2013 | emotional stability | valued traits/characteristics | 289 | -3.291 |
| Garbarino_2013 | openess | valued traits/characteristics | 289 | 1.282 |
| Lindeman_2017 | agreeableness personalit trait | valued traits/characteristics | 55 | -2.575829 |
| Lindeman_2017 | emotional stability personality trait | valued traits/characteristics | 55 | -0.426148 |
| Lindeman_2017 | conscientiousness personality trait | valued traits/characteristics | 55 | 0.568051 |
| Lindeman_2017 | emotional intelligence | valued traits/characteristics | 55 | -2.326348 |
| Lorente Prieto_2008 | personal resources (emotional competences) | valued traits/characteristics | 274 | -2.5 |

# Job attitudes

| Article | Factor's original name | Included in | Sample size | Z-score |
|---|---|---|---|---|
| Lapointe_2011 | normative commitment | extrinsicaly motivated behavior | 260 | 2.32634789 |
| Lapointe_2011 | continuance-sacrifice commitment | extrinsicaly motivated behavior | 260 | 1.28155157 |
| Lapointe_2011 | continuance-alternatives commitment | extrinsicaly motivated behavior | 260 | 2.32634788 |
| Hudek-Knezevic_2011 | continuance commitment | extrinsicaly motivated behavior | 118 | -1.0110343 |
| Tonjes_2009 | performance-avoidance goal orientation | extrinsicaly motivated behavior | 80 | 2.57582932 |
| Childs_2012 | socially prescribed perfectionism | extrinsicaly motivated behavior | 69 | 1.28155157 |
| Lapointe_2011 | affective commitment | intrinsicaly motivated behavior | 260 | -1.2815516 |
| Fernet_2010 | self-determined work motivation | intrinsicaly motivated behavior | 276 | -1.2815516 |
| Petrou, 2015 | self-initiated resources seeking | intrinsicaly motivated behavior | 580 | -0.7891917 |
| Petrou, 2015 | self-initiated challenges seeking | intrinsicaly motivated behavior | 580 | -2.3263479 |
| Petrou, 2015 | self-initiated reducing demands | intrinsicaly motivated behavior | 580 | 2.32634787 |
| Instrand_2011 OLBI | goal orentiation | intrinsicaly motivated behavior | 308 | 1.28155159 |
| Tonjes_2009 | learning goals orientation | intrinsicaly motivated behavior | 80 | -2.3263479 |
| Childs_2012 | self oriented perfectionnism | intrinsicaly motivated behavior | 69 | 1.28155158 |
| Turgut_2016 | resistance to change | negative job attitude | 709 | 3.66666667 |
| Petrou, 2015 | sensitivity to change | negative job attitude | 580 | 2.57582934 |
| Garbarino_2013 | overcomitment | negative job attitude | 289 | 3.29052675 |
| Birkeland_2018 | obsessive passion | negative job attitude | 1263 | 9 |
| Fernet_2014 | obsessive passion | negative job attitude | 175 | 2.57582931 |
| Lavigne_2012 | obssesive passion | negative job attitude | 113 | 2.57582932 |
| Fritz_2006 (OLBI) | negative work reflection | negative job attitude | 221 | 2.57582929 |
| Gonzales-Morales_2010 | absenteeism rate | negative job attitude | 555 | -1 |
| Lu_2013 | sickness presenteeism | negative job attitude | 245 | 3.29052675 |
| Lapointe_2013 | organizational commitment | positive job attitude | 224 | -2.3263479 |
| Lapointe_2013 | commitment to the supervisor | positive job attitude | 224 | -1.2815516 |
| Hudek-Knezevic_2011 | affective-normative commitment | positive job attitude | 118 | -1.9953933 |
| Birkeland_2018 | hamonious passion | positive job attitude | 1263 | -9 |
| Fernet_2014 | hamonious passion | positive job attitude | 175 | -2.5758293 |
| Fritz_2005 OLBI | positif work reflection | positive job attitude | 87 | -1.959964 |
| Fritz_2006 (OLBI) | positive work reflection | positive job attitude | 221 | -1.2815516 |
| Pomaki_2009 | perception of goal attainability | positive job attitude | 222 | -1.8 |

# Personal events

| Article | Factor's original name | Included in | Sample size | Z-score |
|---|---|---|---|---|
| Fritz_2005 | social activity (week end) | leisure | 87 | -1.281552 |
| Fritz_2006 (OLBI) | relaxation | leisure | 221 | -1.281552 |
| Fritz_2006 (OLBI) | mastery | leisure | 221 | -2.326348 |
| Fusilier_2005 | physical exerices | leisure | 260 | -1.281552 |
| Ragsdale_2016 | cell phone attachement | leisure | 59 | -2.575829 |
| Fritz_2005 | non work hassles | stressfull life events | 87 | -1.281552 |
| Fritz_2006 (OLBI) | nonwork hassles | stressfull life events | 221 | 2.326348 |
| Fusilier_2005 | stressfull life events | stressfull life events | 260 | 2.326348 |
| Jensen_2017 | psychological health complaints | stressfull life events | 1702 | 3.290527 |
| Park, 2016 | mental heatlh | stressfull life events | 152 | 1.644737 |

## Work-family interface

| Article | Factor's original name | Included in | Sample size | Z-score |
|---|---|---|---|---|
| Innstrad_2008 (OLBI) | family to work conflict | family-work conflict | 1565 | 2.33 |
| Instrand_2011 OLBI | family work conflict | family-work conflict | 308 | 1.28 |
| Westman_2008 | family-work conflict | family-work conflict | 66 | 2.81 |
| Instrand_2011 OLBI | family work facilitation | family-work facilitation | 308 | 1.28 |
| Gregory_2015 | values | value congruency | 153 | -3.89 |
| Instrand_2011 OLBI | value congruency | value congruency | 308 | 1.28 |
| Jimenez_2017_1 | values (" my values and the organization's values are alike") | value congruency | 141 | 0.23 |
| Hertzberg_2016 | work home interface stress | work-family conflict | 274 | -3.29 |
| Hornung_2013 | work-family conflict | work-family conflict | 95 | 1.28 |
| Innstrad_2008 (OLBI) | work to family conflict (pressure at work hamper functioning at home) | work-family conflict | 1565 | 2.33 |
| Instrand_2011 OLBI | work-home conflict | work-family conflict | 308 | -2.33 |
| Jensen_2017 | work-family conflict | work-family conflict | 1702 | 3.29 |
| Lizano_2012 | work family conflict | work-family conflict | 335 | 5.54 |
| Lizano_2015 | work family conflict | work-family conflict | 361 | 2.58 |
| Mauno_2015 | work family conflict | work-family conflict | 814 | 2.58 |
| Ragsdale_2016 | work related cell phone use (during non work time) | work-family conflict | 59 | -1.28 |
| Richter_2015 | work– family conflict | work-family conflict | 3378 | 1.28 |
| Rubio_2015 | work-family conflict | work-family conflict | 242 | 2.33 |
| Travis_2016 | work-family conflict | work-family conflict | 362 | 3.25 |
| Westman_2008 | work-family conflict | work-family conflict | 66 | 2.81 |
| Innstrad_2008 (OLBI) | work to family facilitation | work-family facilitation | 1565 | -2.33 |
| Instrand_2011 OLBI | work-home facilitation | work-family facilitation | 308 | 1.28 |
| Mauno_2015 | work family enrichment | work-family facilitation | 814 | -2.33 |

## Perceived intermediate work consequences

| Article | Factor's original name | Included in | Sample size | Z-score |
|---|---|---|---|---|
| Figueiredo-Ferraz_2012 | work satisfaction | job satisfaction | 316 | -1.28 |
| Lindeman_2017 | total positive work experiences | job satisfaction | 55 | -2.97 |
| Poulin, 1993 | satisfaction with clients | job satisfaction | 879 | -2.33 |
| Dahlin_2010 OLBI | worries about futur endurance/competence | stress | 186 | 3.29 |
| Fusilier_2005 | stressfull work events | stress | 260 | 1.28 |
| Hornung_2013 | patient demands | stress | 95 | -2.58 |
| Laugaa_2008 | perceived stress | stress | 259 | 2.58 |
| Lizano_2012 | job stress | stress | 335 | 0.30 |
| McManus_2002 | stress | stress | 365 | 2.33 |
| Poulin, 1993 | job stress | stress | 879 | 3.29 |
| Taris_2001 | stressors (Students) | stress | 828 | -2.58 |
| Taris_2001 | stressors (colleagues) | stress | 828 | -2.58 |
| Van_der_Ploeg_2003 | job physicial strain | stress | 123 | 2.33 |

**Families of predictors and related theories**
In order to perform further analysis, factors were first classified into families and sub-families of predictors on the basis of the predictors' nature and the existent literature in order to create groups that were as homogenous as possible.

1. Classification overview and methodology

First, based on review of Maslach et al. (2001) on job burnout, we considered two main families of factors: situational factors and individual factors. Job characteristics and organizational characteristics were included in the former whereas personality characteristics, work attitudes and non-occupational factors were included in the latter.
Moreover, as a category at the intersection between work and personal life, we considered a third main family: work-life interface (Greenhouse & Allan, 2011; Rubio et al., 2015) which refers to factors of personal life that intersect with work factor or vice-versa. Finally, we considered the effect of other job outcomes (than exhaustion) as a fourth main family.
From the four main families, we categorized factors into subfamilies until all predictors of one subfamily 1) referred to a relatively homogenous construct and 2) had the same valence/direction (i.e. 2 subfamilies "maladaptive coping style" and "adaptative coping style" instead of one subfamily "coping style"). We will now describe the literature we leant on in in order to get to that result.

2. Situational characteristics

We referred to the Job-Demands-Control (JDC) model first proposed by Robert Karasek in 1979 and later modified by Johnson & Hall (1988) as the Job-Demands- Control-Support (JDCS) model to consider job demands, decision latitude (control) and social support as subfamilies of situational characteristics.

Job demands refers to requirements/stressors that are set at work including workload, time pressure, physical demands and emotional demands.
Decision latitude (control) is the extent to which an employee has potential control over decisions concerning when, where and how to perform work tasks including job variety (or skill discretion; degree to which job involves a variety of tasks, low repetitiveness, occasions for creativity and opportunities to learn new things) and decision authority ( the ability to make its own decisions about how the work is done - i.e. autonomy - as well as influence work team and policies). In regard to control, Rafferty et al. (2001) highlighted how "inconsistent findings may be related to the different ways in which control has been defined and suggest that future research on the JDC model should differentiate between dimensions of control". They indeed showed that exhaustion was more or less associated with higher control depending on how it was defined; authors founds positive association when defined as decision latitude – skill discretion plus decision authority – and autonomy (Landsbergis, 1988 ; de Jonge et al., 1996) , inconsistent association when defined as decision authority (significant in one study but not the other: Taris et al., 1999; de Rijk et al., 1998) and no relation when defined as skill discretion (Taris et al., 1999).
Social support is defined as "the perception and actuality that one is cared for, has assistance available from other people, and most popularly, that one is part of a supportive social network". Support can be of different type such as emotional support, companionship (sense of belonging) or informational (e.g. advice) and can come from many sources such as family, colleagues, supervisor and/or organizations.

According to those models, employees working in situation with high demands, low control and low social support /isolation (specific to JDCS model) experience the lowest well-being ((iso)-strain hypothesis). Conversely, high job control and social support can moderate the negative impact of job demands on well-being (buffer hypothesis).

Alongside, we referred to Job-Demand-Resources model proposed by Demerouti et al. (2001) as an alternative to the model to the JDC(S) models, to consider job resources as a subfamily of situational characteristics.
Job resources are defined as job-relevant features that help workers achieve work-related goals, lessen job demands, or stimulate personal growth.
According to this model, employee high strain and low well-being is a response to imbalance between demands and resources he or she has to deal with those demands.

Later on, in 2006, Akerboom and Maes proposed the organizational risk factor questionnaire derived from the Tripod accident causation model (Wagenaar, Hudson, & Reason, 1990; Wagenaar, Groeneweg, Hudson, & Reason, 1994) to investigate determinants of psychological well-being from a broader organizational perspective than the JDC(S) model does. These authors considered factors such as “organization (OR)” – which refers to the effectiveness of the organization’s structure and processes, and management strategies; communication (CO) – which refers to quality and effectiveness of communications between individuals; and social hindrance – operationalized as disrespectful, unequal and/or unfair treatment, lack of appreciation of extra-effort, etc. Ruehlman and Wolchik (1988) defined social hindrance as “behaviors that are perceived by either the actor or the target person as a) (un-)intentional interference with goal-directed activity or b) (in-) direct expressions of anger or other negative emotions or c) (in-)direct negative evaluations of the target person’s character or behavior”.
In view of our data and considering those definitions, we included social hindrance, informational climate, lack/conflicting information, enriching leadership and non-collaborative leadership as subfamilies of organizational characteristics.

3. Individual characteristics

We referred to Maslach et al. (2001) to consider two groups of subfamilies in the individual characteristics: personality characteristics (including intrinsic characteristics, coping strategies and self-evaluation) and job attitude (work-related attitudes including job involvement and unfavorable job attitude). We also considered non-occupational factors as part of the individual characteristics.
Subfamily “intrinsic characteristics” mainly refers to the Big Five personality traits proposed by Goldberg in 1981 and further developed by Costa & McCrae in the late 80s. The Big Five or “OCEAN” refers to a descriptive model of personality in 5 main traits including openness to experience, conscientiousness, extraversion, agreeableness and emotional stability (inverse of neuroticism). Other intrinsic characteristics including emotional intelligence, optimism, type a behavior and hardiness were also included in this subfamily.
Coping was defined by Lazarus & Folkman in 1984 as “cognitive and behavioral efforts aiming to control, reduce or tolerate demands that threaten or exceed and individual resources”. In short, coping refers to efforts to manage stress. In order to fit with the criterion pertaining to classification cited above, coping strategies were divided into two subfamilies, namely, adaptative coping (i.e. strategies that involve confronting problems directly, decrease the actual level of stress and improve long term level of functioning) and maladaptive coping

style (i.e. strategies that reduce the symptoms related to stress for a short time, but have no influence on the thing that is causing the stress ) and were considered separately.
Self-evaluation included self-esteem – introduced for the first time by William James in 1890, refers to "an individual's sense of value or self-worth, or the extent to which people value, appreciate or like themselves" – and self-efficacy – defined as the belief individuals have in their ability to succeed and their level of competence (Bandura & Adams, 1977). It is often argued that self-esteem and self-efficacy are distinct constructs (Lane et al., 2004). However, a reasonable assumption is that people who have high global self-esteem will predict higher probability of task success (high self-efficacy) and hence they relate both theoretically and empirically (Gardner & Pierce,1998).

In the subfamily "job attitude", we included predictors referring to job involvement (commitment, presenteeism, etc.) and unfavorable job attitudes (absenteeism, overcommitment, resistance to change). The diversity of predictors also enabled us to make the distinction between intrinsic job involvement (Georges, 1992) and extrinsic job involvement, which we considered as two other subfamilies. The former involves doing something because it is personally rewarding while the latter involves doing something for reward or to avoid punishment.
Finally, non-occupational factors included any determinants related to personal life events.

4. Work-life interface

Work-life interface refers to either positive or negative spillover, defined as a process whereby behavior in one domain establishes benefits/resources (positive spillover; enrichment) or detriments (negative spillover; conflict) which then, respectively, improve or impair performance/involvement in the other domain (Greenhaus & Allen, 2011). More precisely, work - life conflict occurs when the demands associated with one domain are incompatible with the demands associated with another domain whereas work-life facilitation refers to how participation in one role is made better or easier by participation in the other role (Wayne et al., 2004).
Several authors form out dataset investigated the effect of value congruence. Value congruence refers to the degree to which an individual's values match the ones found in their work environment. As "value congruence between individuals and the organizations in which they work results in a number of beneficial outcomes, the most common of which are associated with positive attitudes and behaviors" (Molina, 2016), we included all predictors pertaining to value congruence in subfamily work-life enrichment.

5. Perceived intermediate work consequences

The two main subfamilies we could form given our data are Job satisfaction and Stress/insecurity from work conditions. This classification was inspired Adriaenssens et al. (2015) operationalization of "predictors of stress–health".
