# Supplementary material for: Predictors of Occupational Burnout: A Systematic Review
Source: Int J Environ Res Public Health. 2021 Aug 31;18(17):9188. doi: 10.3390/ijerph18179188 (PMC8430894; doi:10.3390/ijerph18179188)
Supplement: Supplementary file 1 [file ijerph-18-09188-s001.zip › Table S3.pdf]

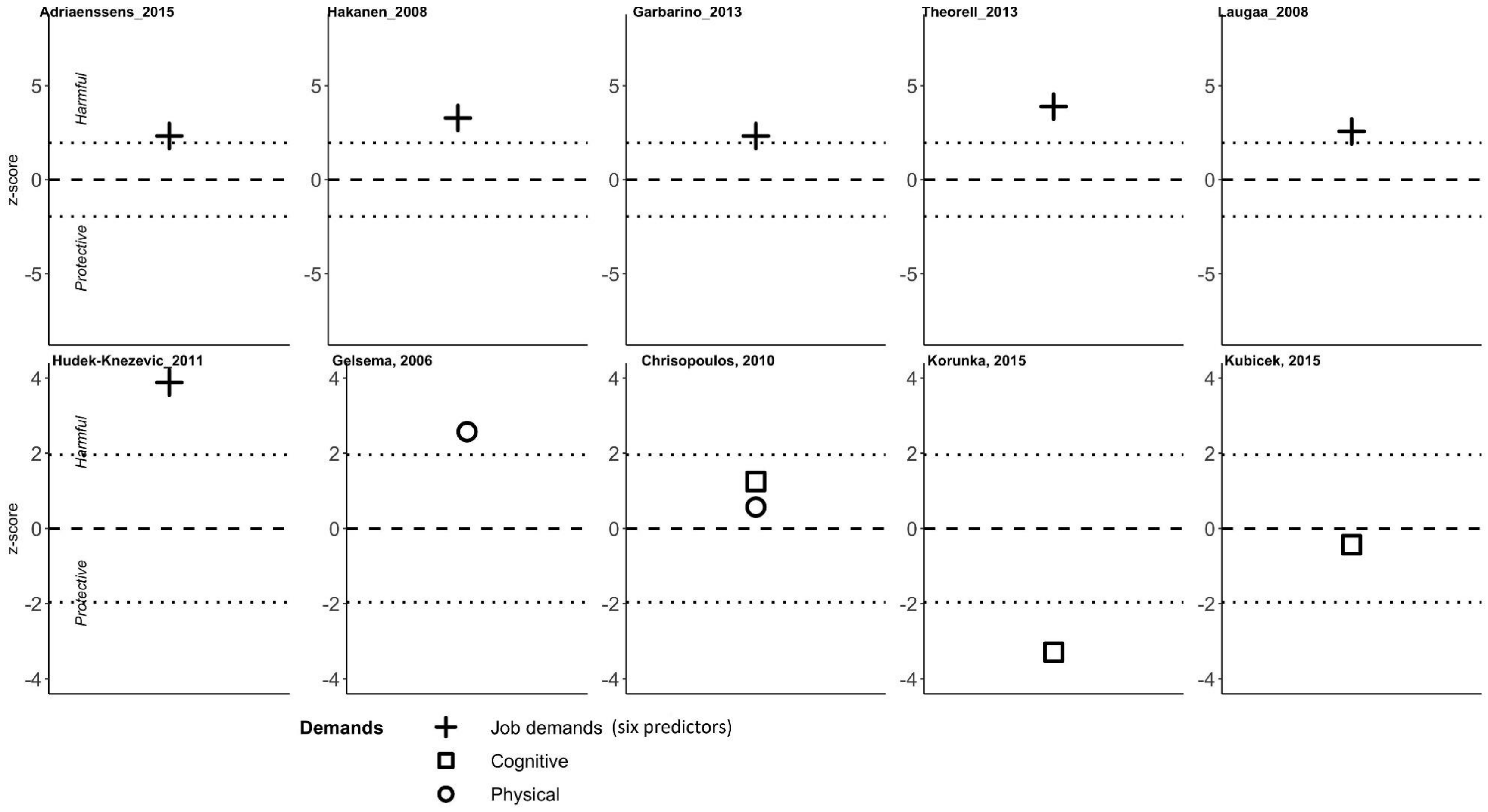

Adriaenssens_2015
Hakanen_2008
Garbarino_2013
Theorell_2013
Laugaa_2008
Hudek-Knezevic_2011
Gelsema, 2006
Chrisopoulos, 2010
Korunka, 2015
Kubicek, 2015
z-score
Harmful
Protective
Demands
Job demands (six predictors)
Cognitive
Physical

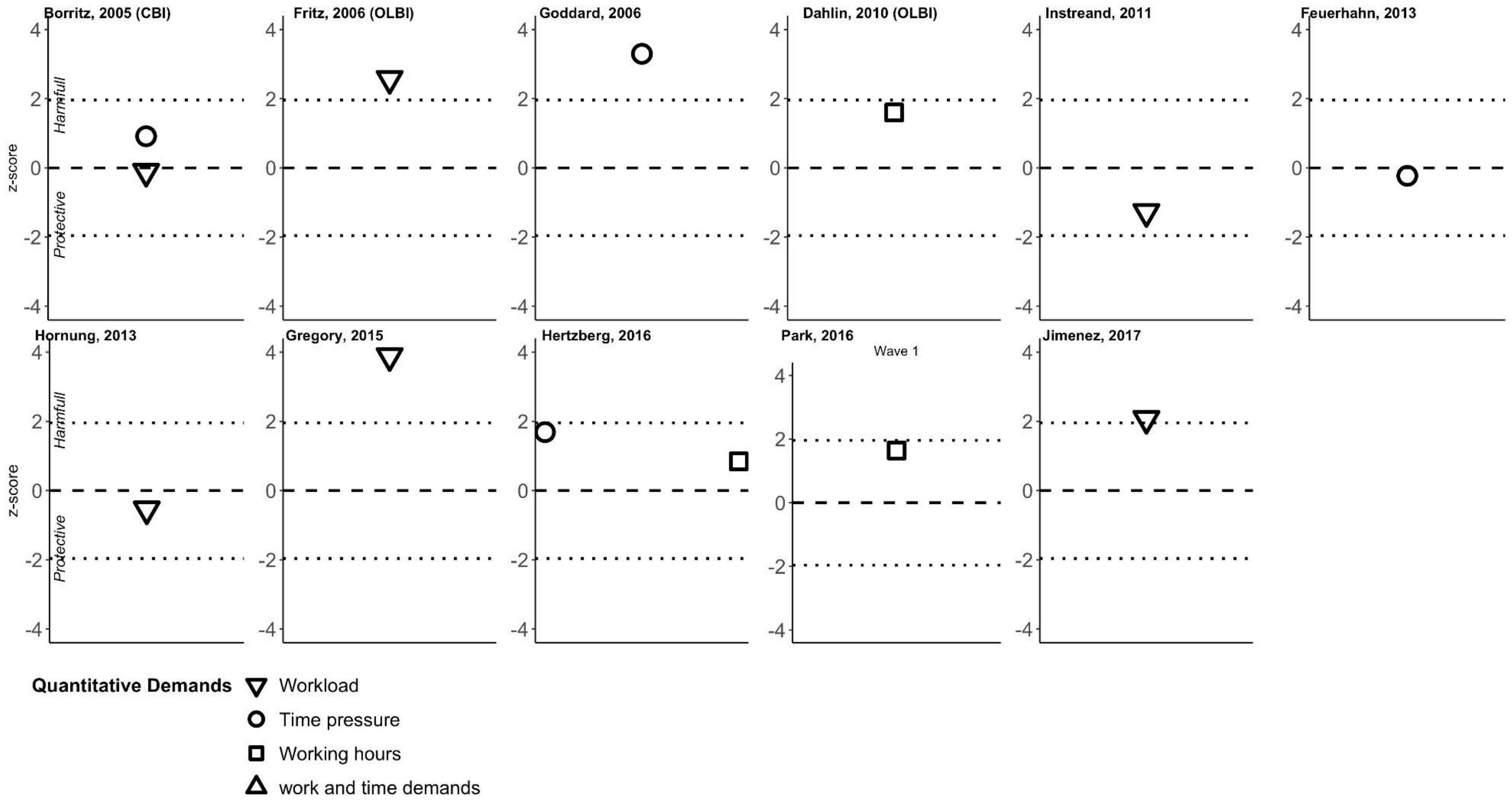

Borritz, 2005 (CBI)
Fritz, 2006 (OLBI)
Goddard, 2006
Dahlin, 2010 (OLBI)
Instreand, 2011
Feuerhahn, 2013
Hornung, 2013
Gregory, 2015
Hertzberg, 2016
Park, 2016
Wave 1
Jimenez, 2017
z-score
Harmfull
Protective
4
2
0
-2
-4
Quantitative Demands
Workload
Time pressure
Working hours
work and time demands

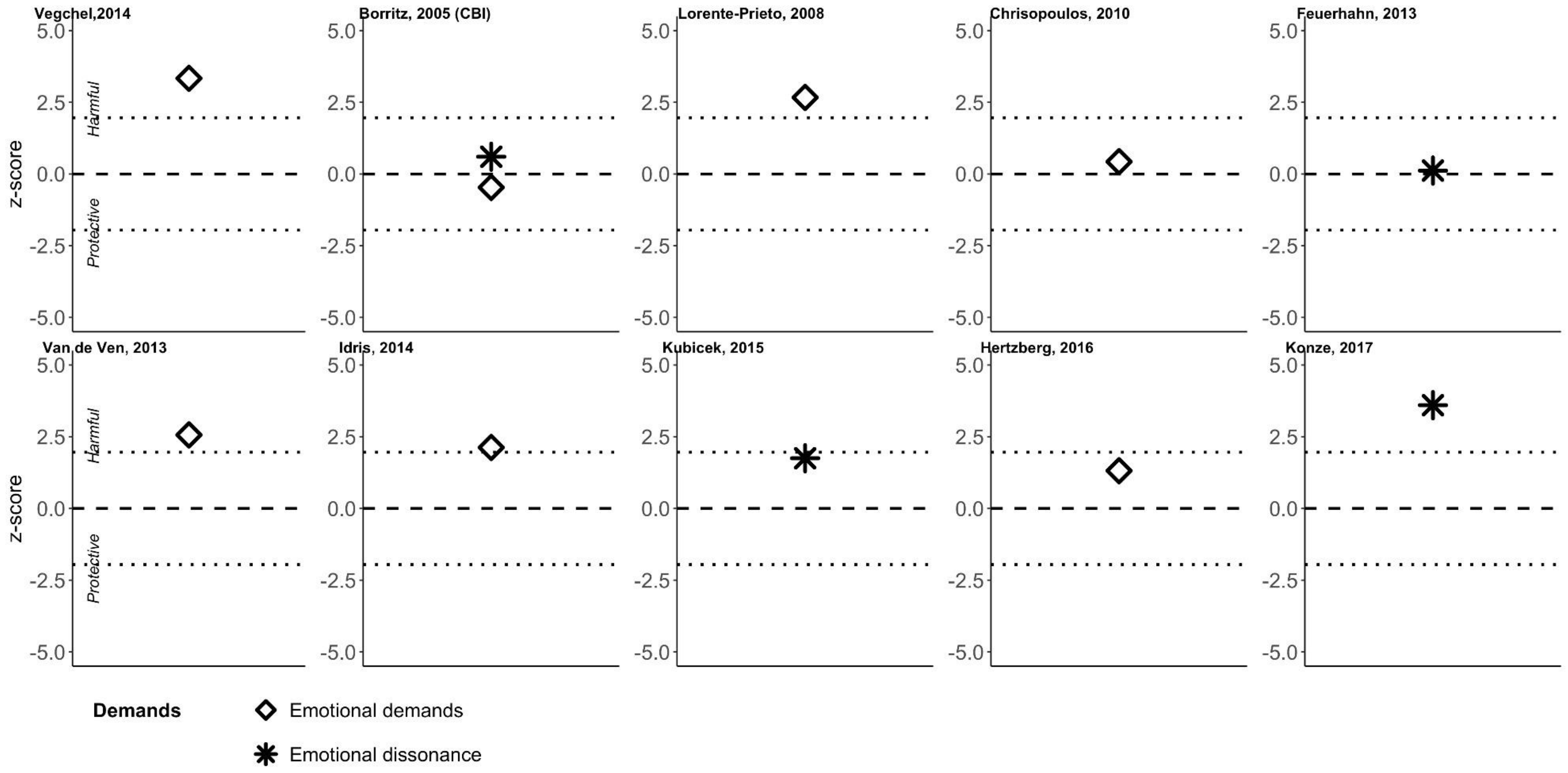
Vegchel,2014
Borritz, 2005 (CBI)
Lorente-Prieto, 2008
Chrisopoulos, 2010
Feuerhahn, 2013
Van de Ven, 2013
Idris, 2014
Kubicek, 2015
Hertzberg, 2016
Konze, 2017
z-score
Harmful
Protective
5.0
2.5
0.0
-2.5
-5.0
Demands
Emotional demands
Emotional dissonance

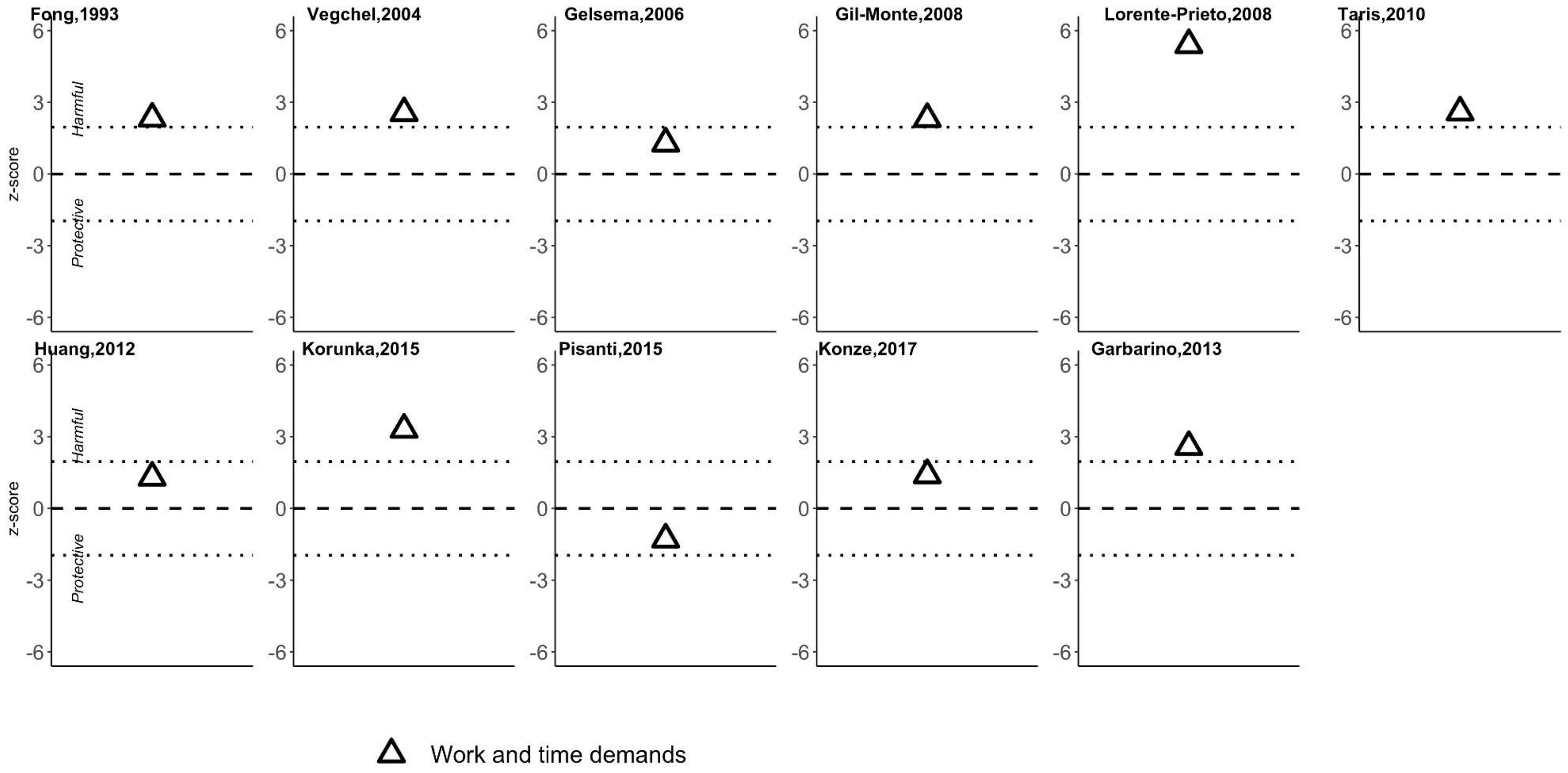

Fong,1993
Vegchel,2004
Gelsema,2006
Gil-Monte,2008
Lorente-Prieto,2008
Taris,2010
Huang,2012
Korunka,2015
Pisanti,2015
Konze,2017
Garbarino,2013
z-score
Harmful
Protective
6
3
0
-3
-6
Work and time demands

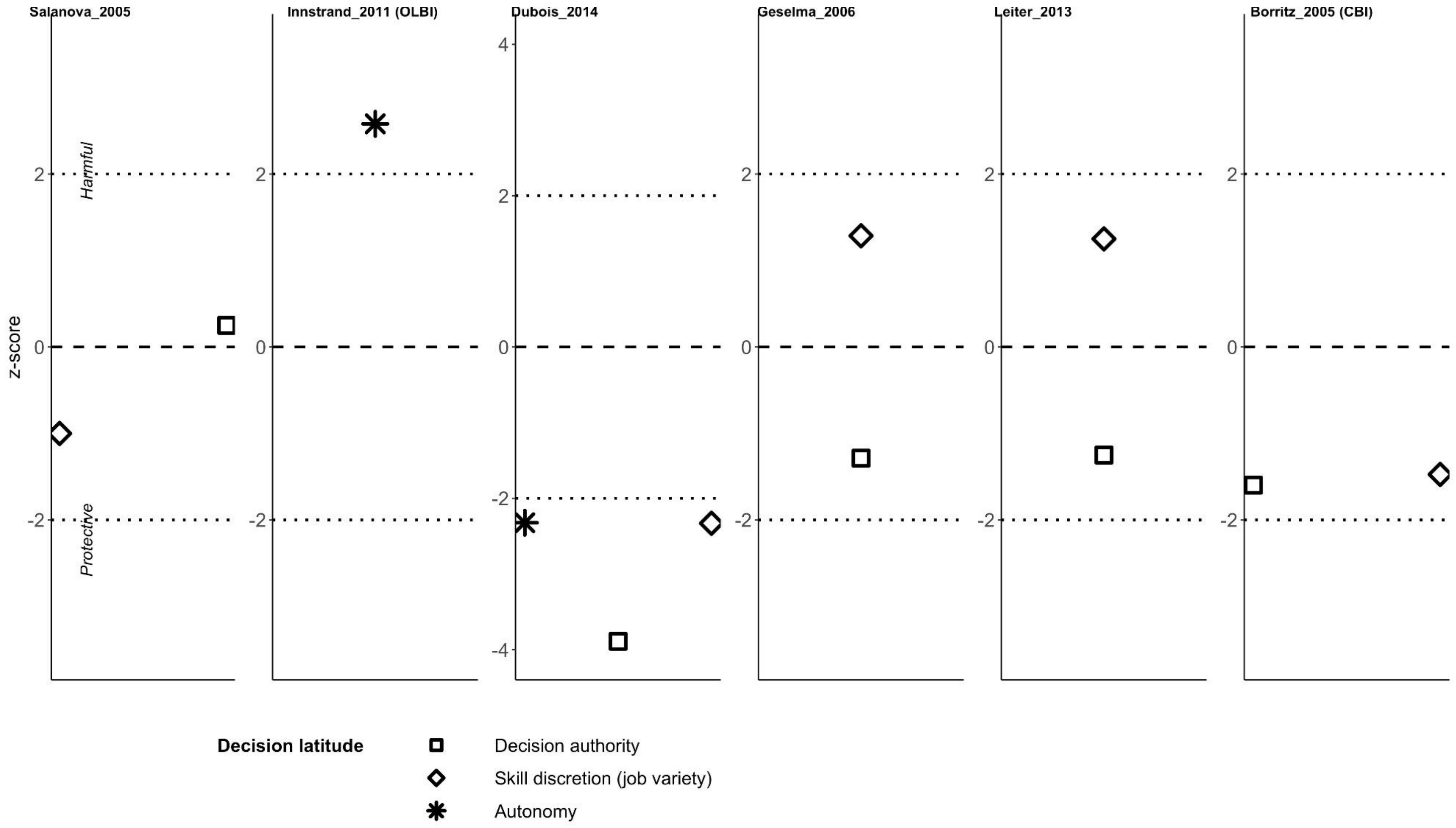

Salanova_2005
Innstrand_2011 (OLBI)
Dubois_2014
Geselma_2006
Leiter_2013
Borritz_2005 (CBI)
z-score
Harmful
Protective
4
2
0
-2
-4
Decision latitude
Decision authority
Skill discretion (job variety)
Autonomy

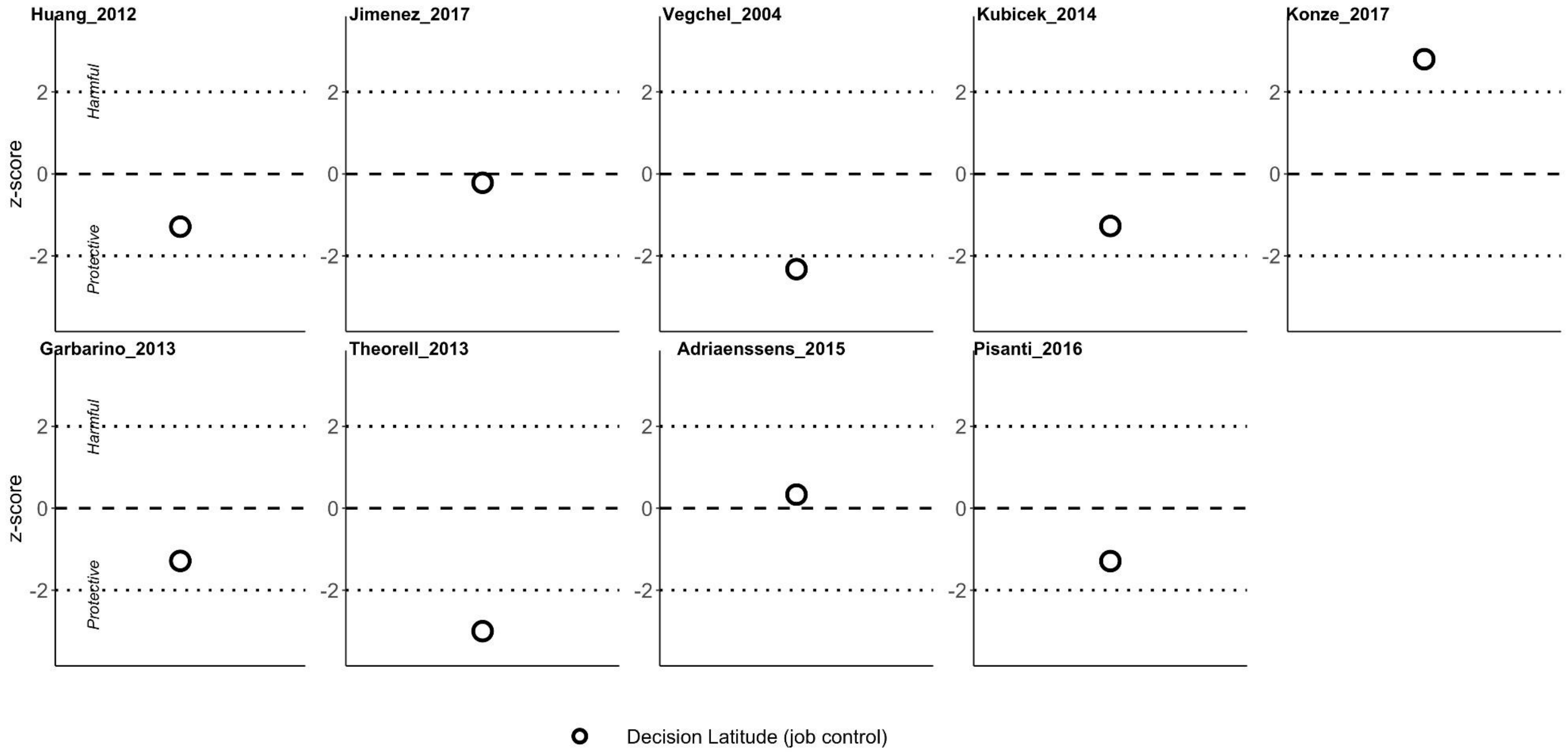

Huang_2012
Jimenez_2017
Vegchel_2004
Kubicek_2014
Konze_2017
Garbarino_2013
Theorell_2013
Adriaenssens_2015
Pisanti_2016
z-score
Harmful
Protective
2
0
-2
Decision Latitude (job control)

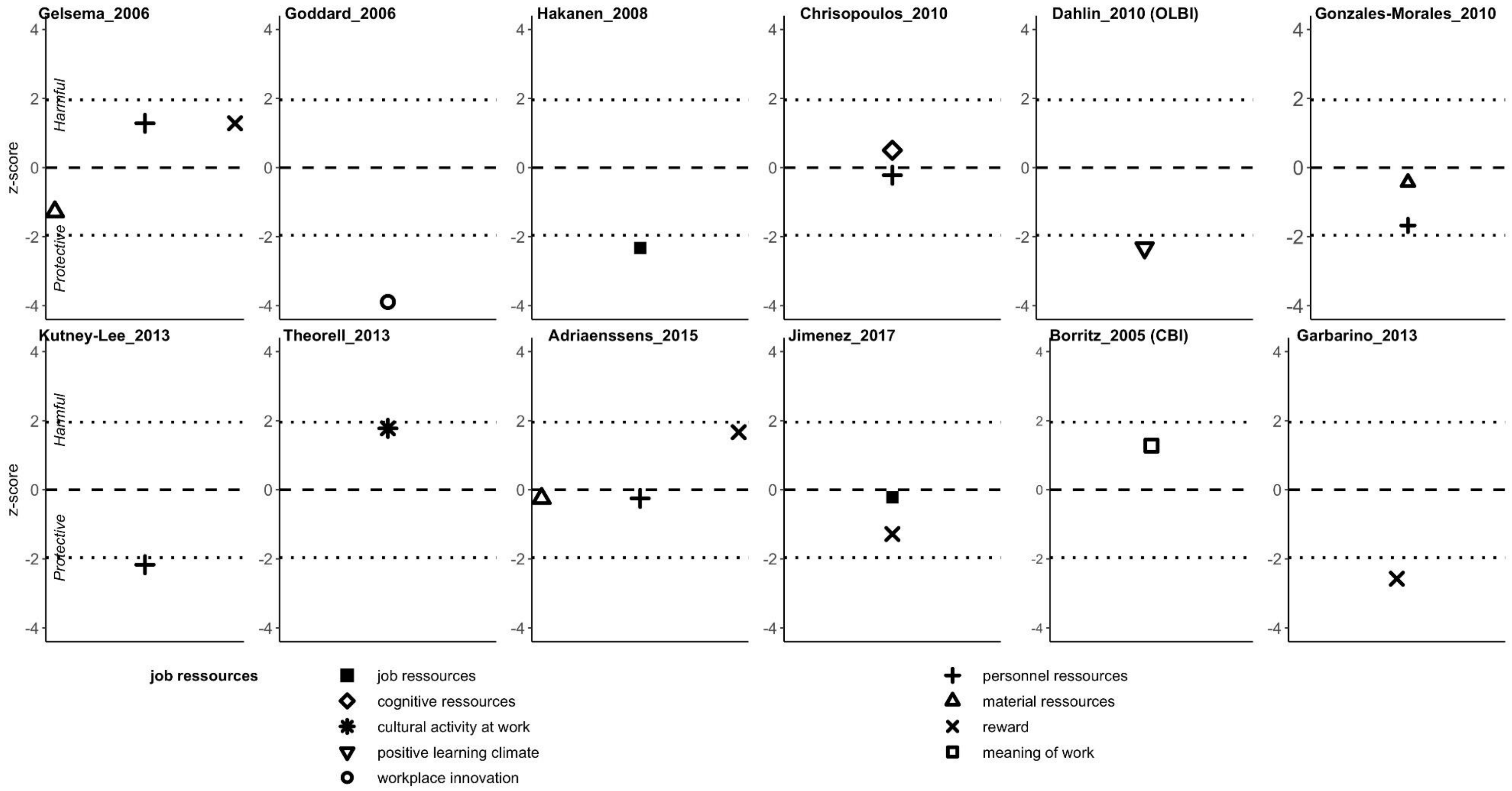
Gelsema_2006
Goddard_2006
Hakanen_2008
Chrisopoulos_2010
Dahlin_2010 (OLBI)
Gonzales-Morales_2010
Kutney-Lee_2013
Theorell_2013
Adriaenssens_2015
Jimenez_2017
Borritz_2005 (CBI)
Garbarino_2013
z-score
Harmful
Protective
4
2
0
-2
-4
job ressources
job ressources
cognitive ressources
cultural activity at work
positive learning climate
workplace innovation
personnel ressources
material ressources
reward
meaning of work

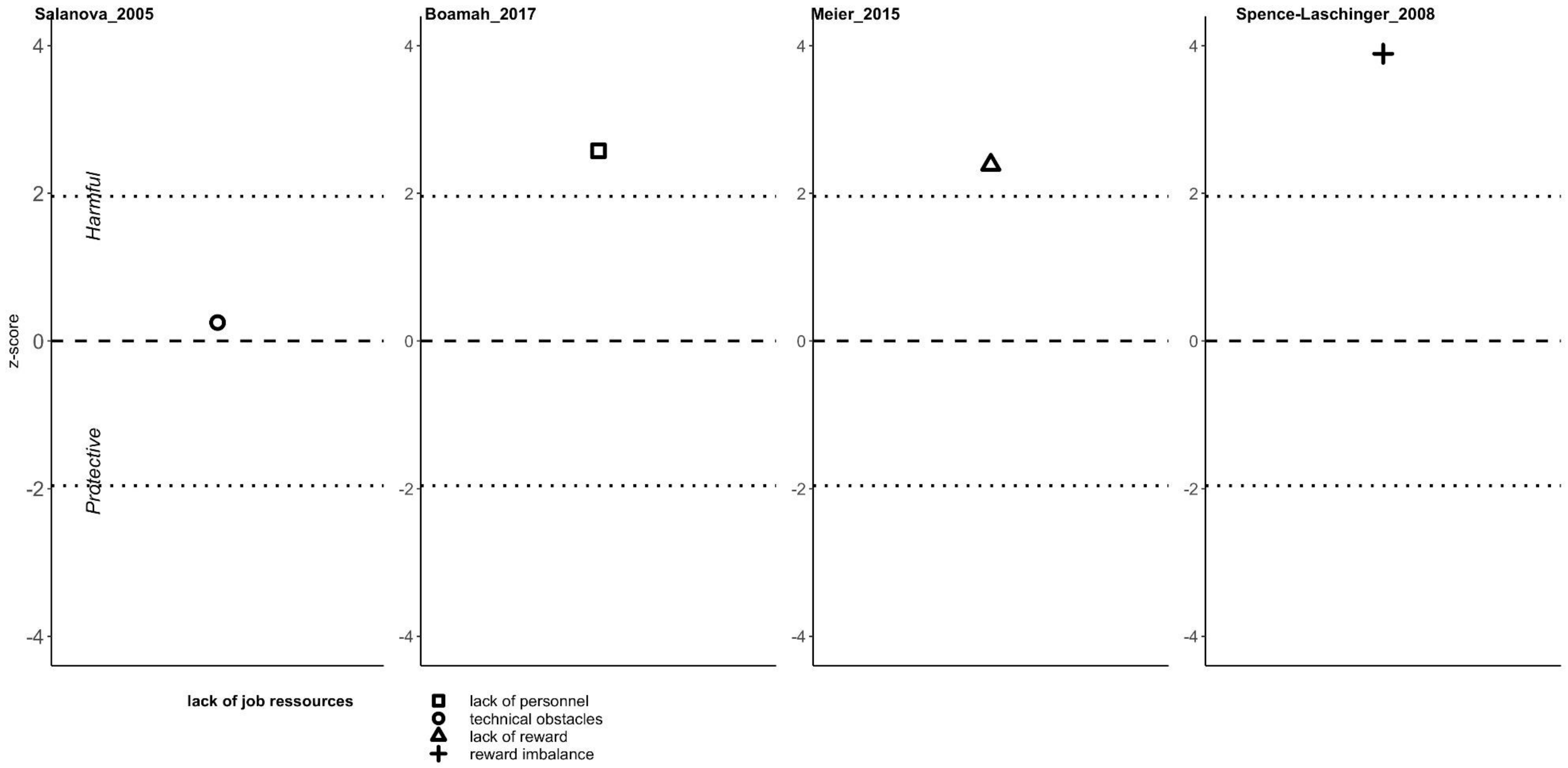

Salanova_2005
Boamah_2017
Meier_2015
Spence-Laschinger_2008
4
2
0
-2
-4
z-score
Harmful
Protective
lack of job ressources
lack of personnel
technical obstacles
lack of reward
reward imbalance

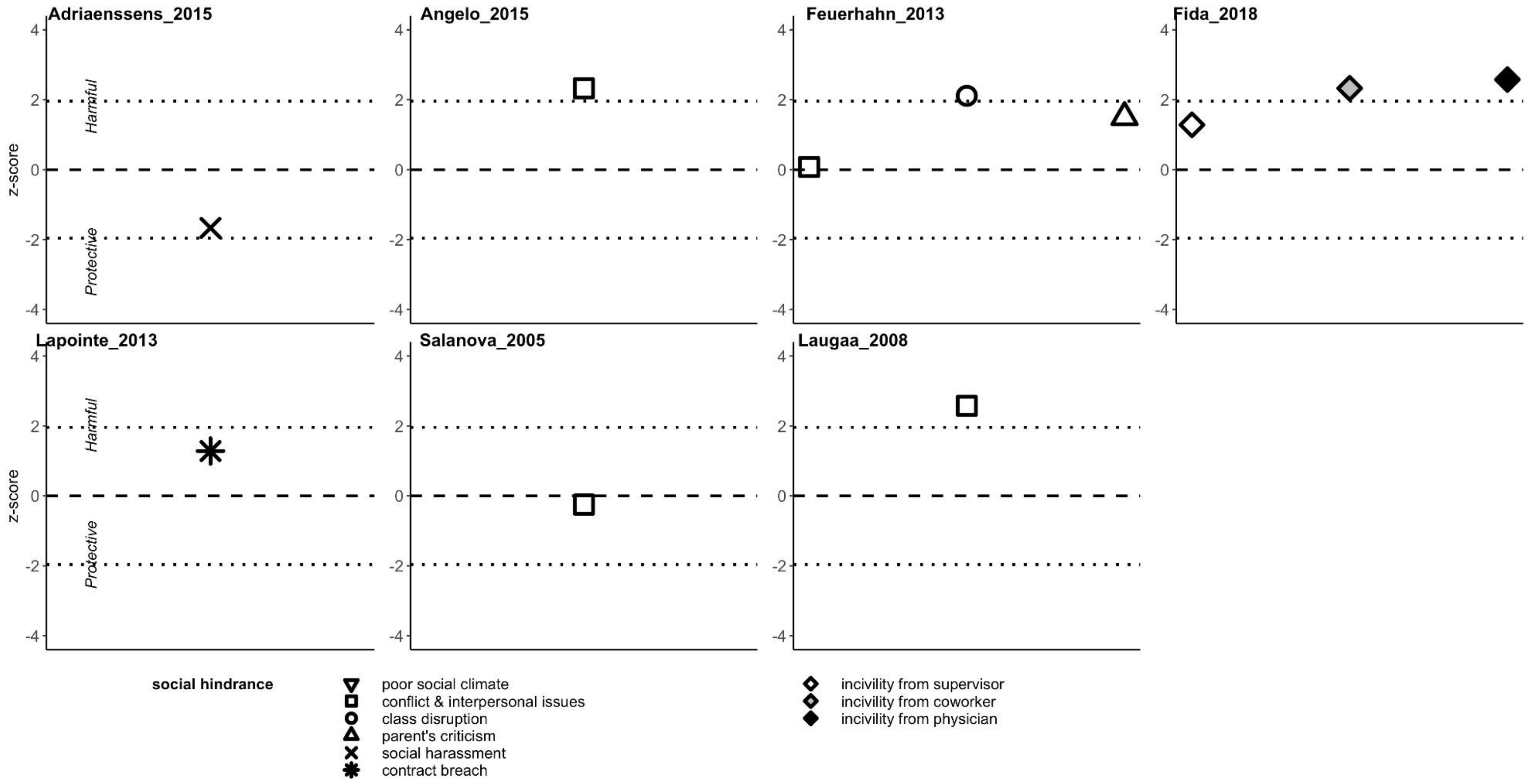

Adriaenssens_2015
Angelo_2015
Feuerhahn_2013
Fida_2018
Lapointe_2013
Salanova_2005
Laugaa_2008
z-score
Harmful
Protective
4
2
0
-2
-4
social hindrance
poor social climate
conflict & interpersonal issues
class disruption
parent's criticism
social harassment
contract breach
incivility from supervisor
incivility from coworker
incivility from physician

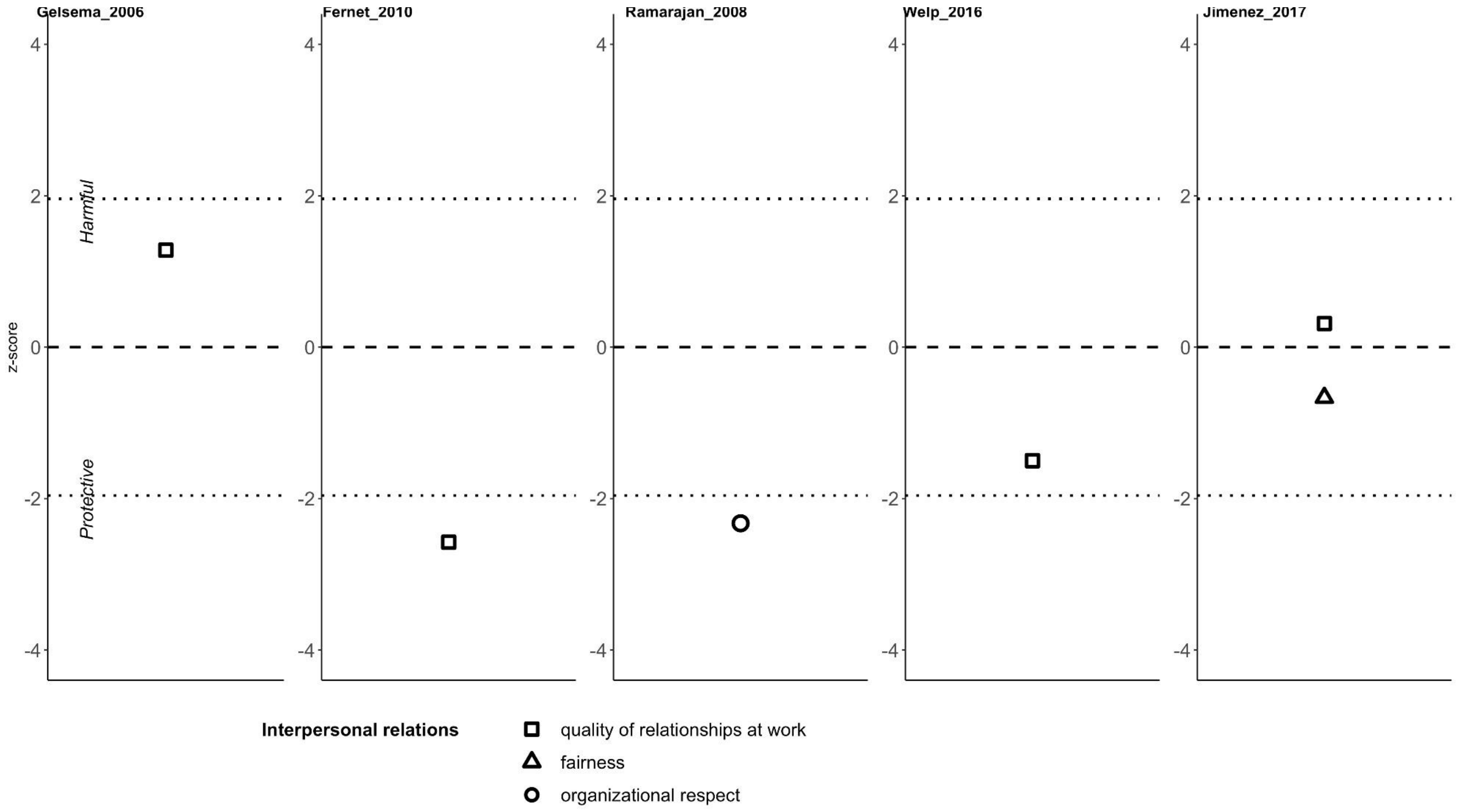
Gelsema_2006
Fernet_2010
Ramarajan_2008
Welp_2016
Jimenez_2017
z-score
Harmful
Protective
4
2
0
-2
-4
Interpersonal relations
quality of relationships at work
fairness
organizational respect

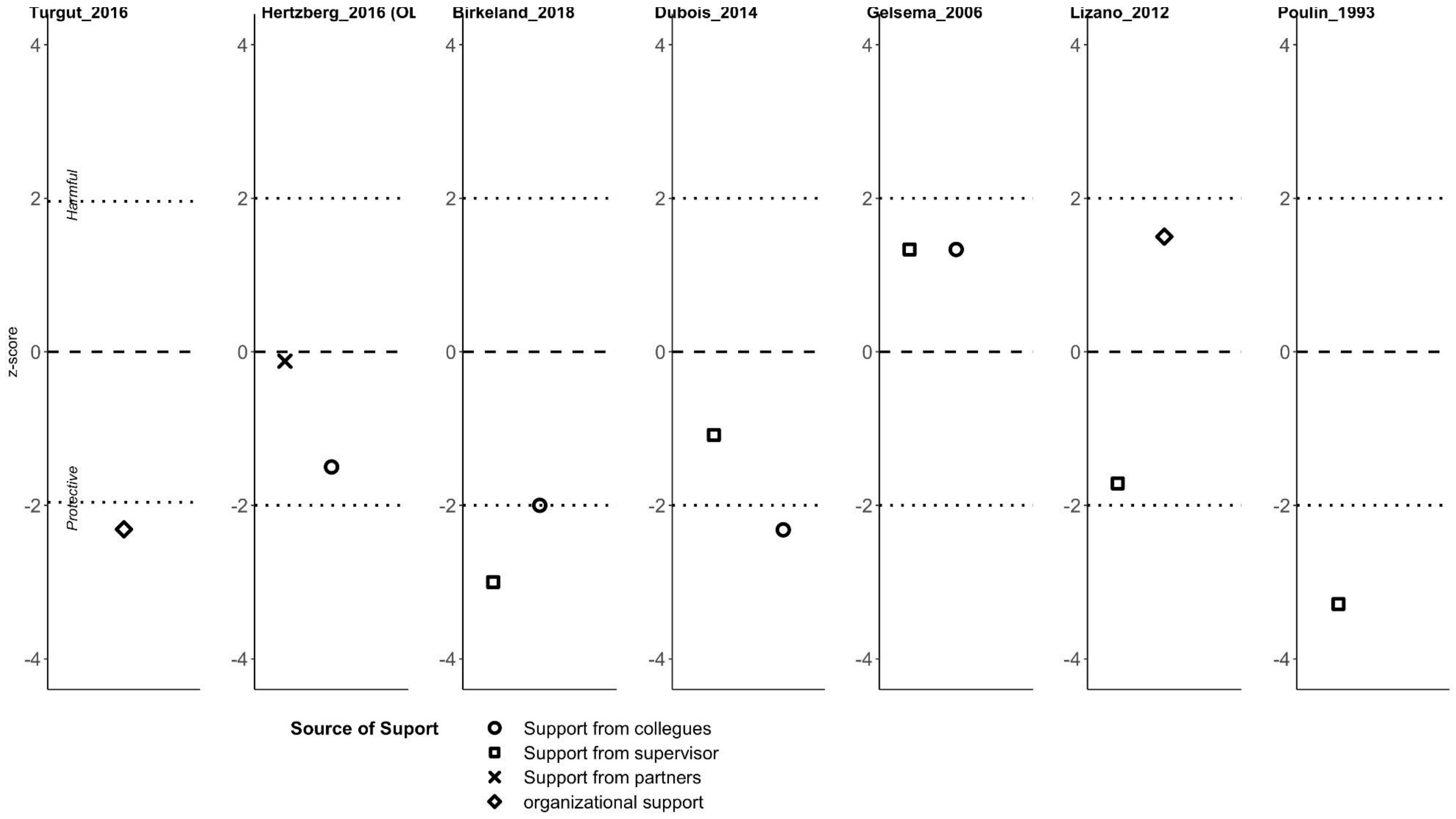
Turgut_2016
Hertzberg_2016 (OL
Birkeland_2018
Dubois_2014
Gelsema_2006
Lizano_2012
Poulin_1993
z-score
Harmful
Protective
4
2
0
-2
-4
Source of Suport
Support from collegues
Support from supervisor
Support from partners
organizational support

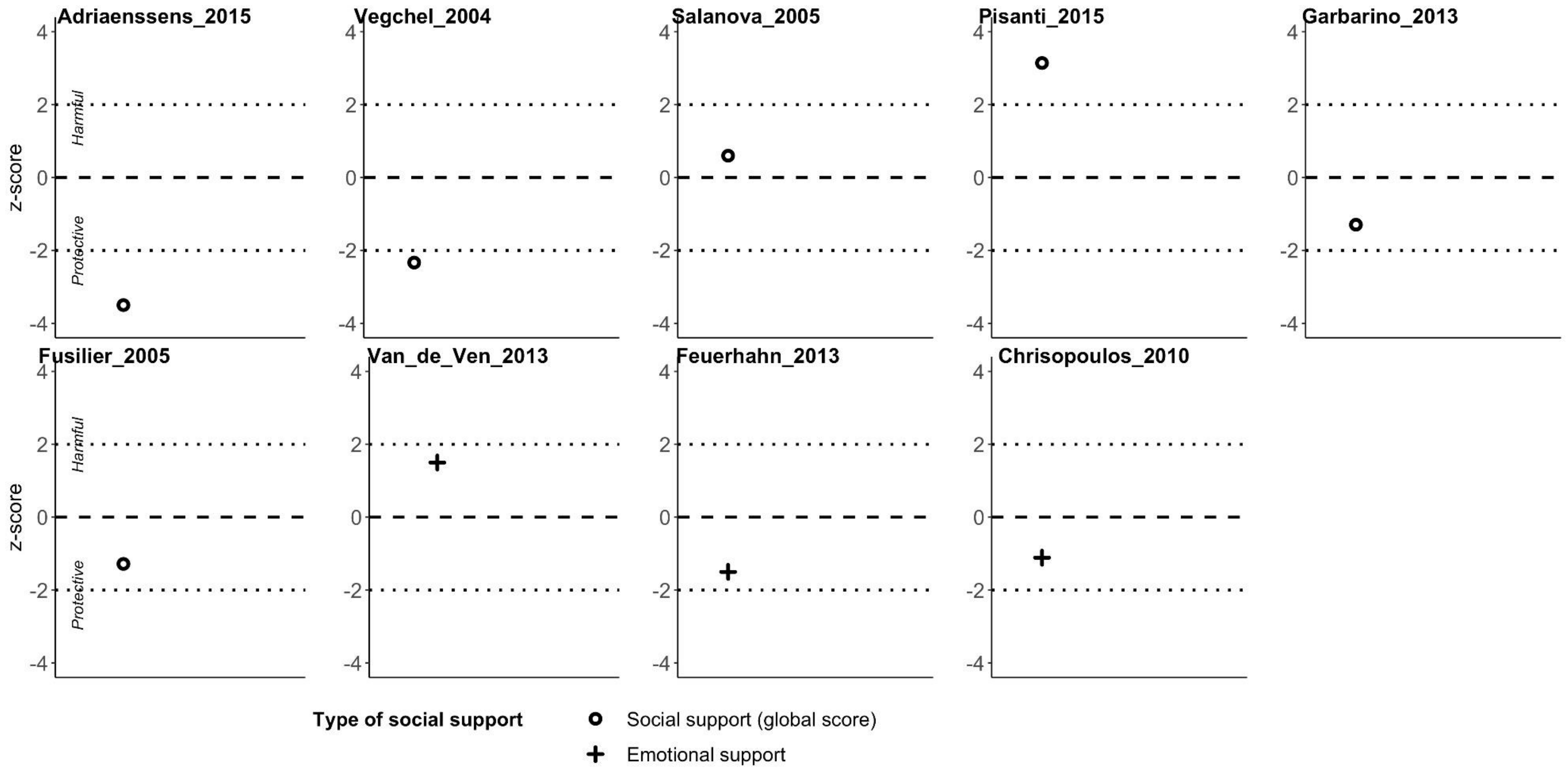
Adriaenssens_2015
Vegchel_2004
Salanova_2005
Pisanti_2015
Garbarino_2013
Fusilier_2005
Van_de_Ven_2013
Feuerhahn_2013
Chrisopoulos_2010
z-score
Harmful
Protective
4
2
0
-2
-4
Type of social support
Social support (global score)
Emotional support

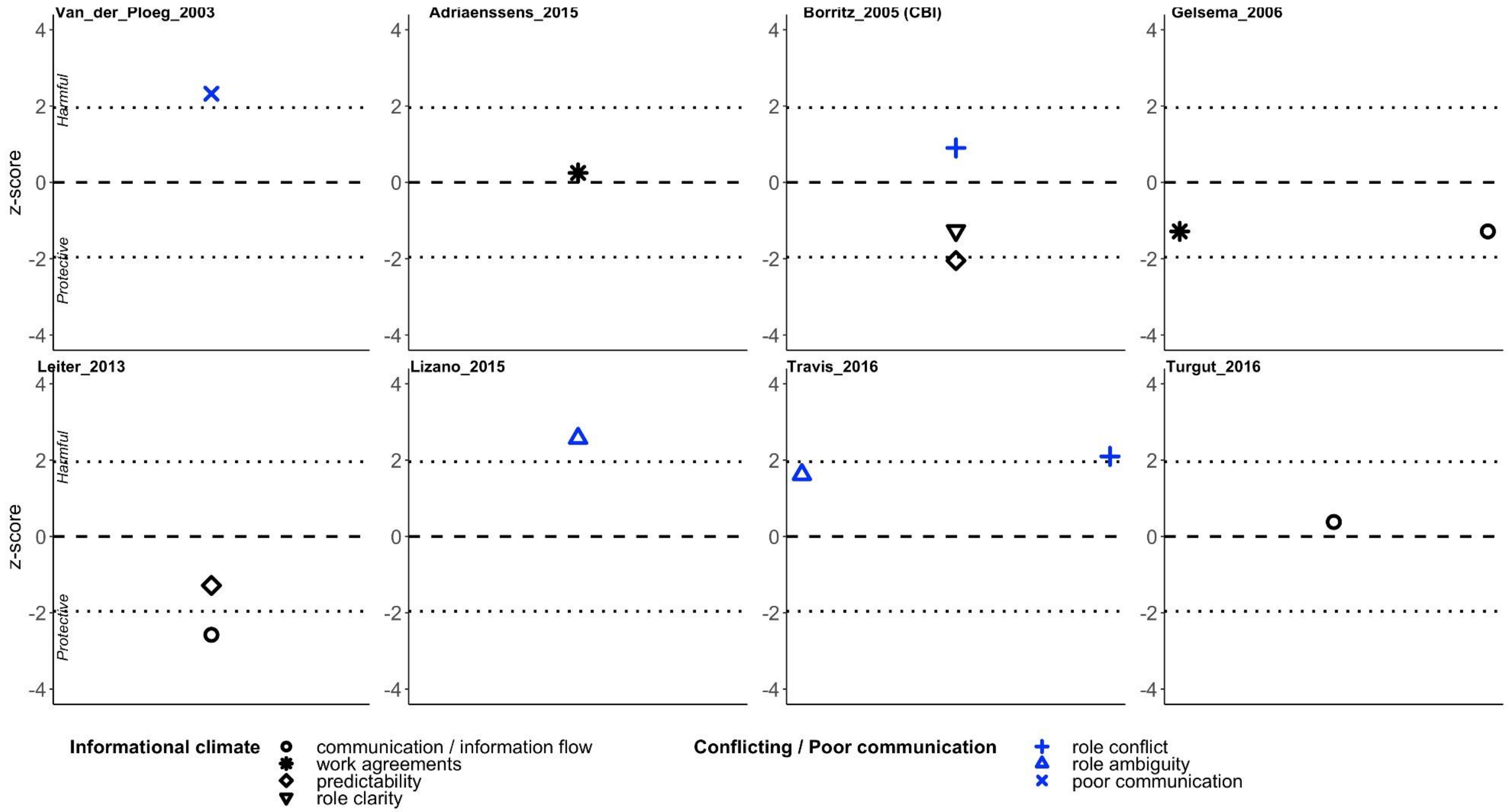
Van_der_Ploeg_2003
Adriaenssens_2015
Borritz_2005 (CBI)
Gelsema_2006
Leiter_2013
Lizano_2015
Travis_2016
Turgut_2016
z-score
Harmful
Protective
4
2
0
-2
-4
Informational climate
communication / information flow
work agreements
predictability
role clarity
Conflicting / Poor communication
role conflict
role ambiguity
poor communication

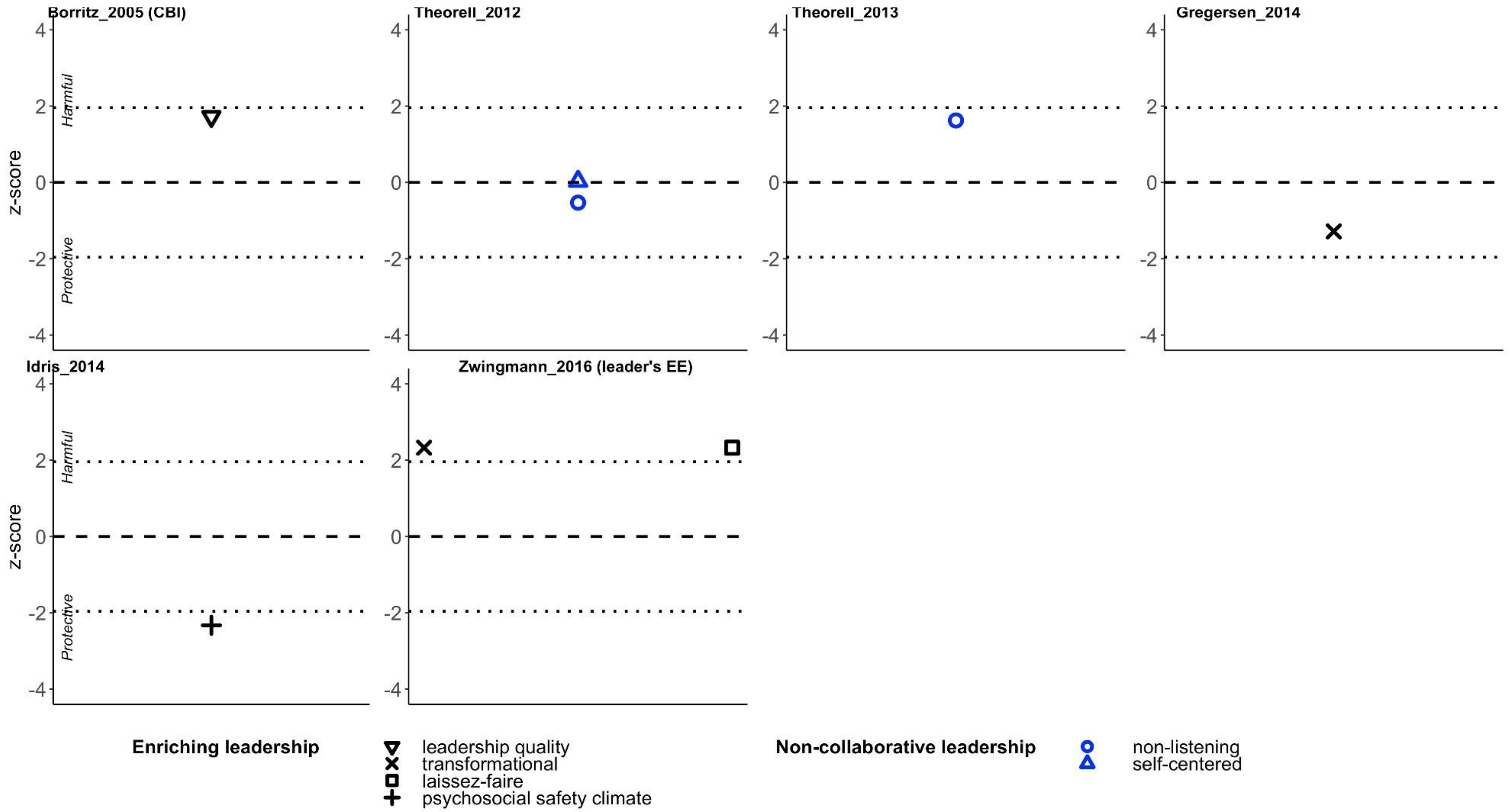

Borritz_2005 (CBI)
Theorell_2012
Theorell_2013
Gregersen_2014
Idris_2014
Zwingmann_2016 (leader's EE)
z-score
Harmful
Protective
4
2
0
-2
-4
Enriching leadership
leadership quality
transformational
laissez-faire
psychosocial safety climate
Non-collaborative leadership
non-listening
self-centered

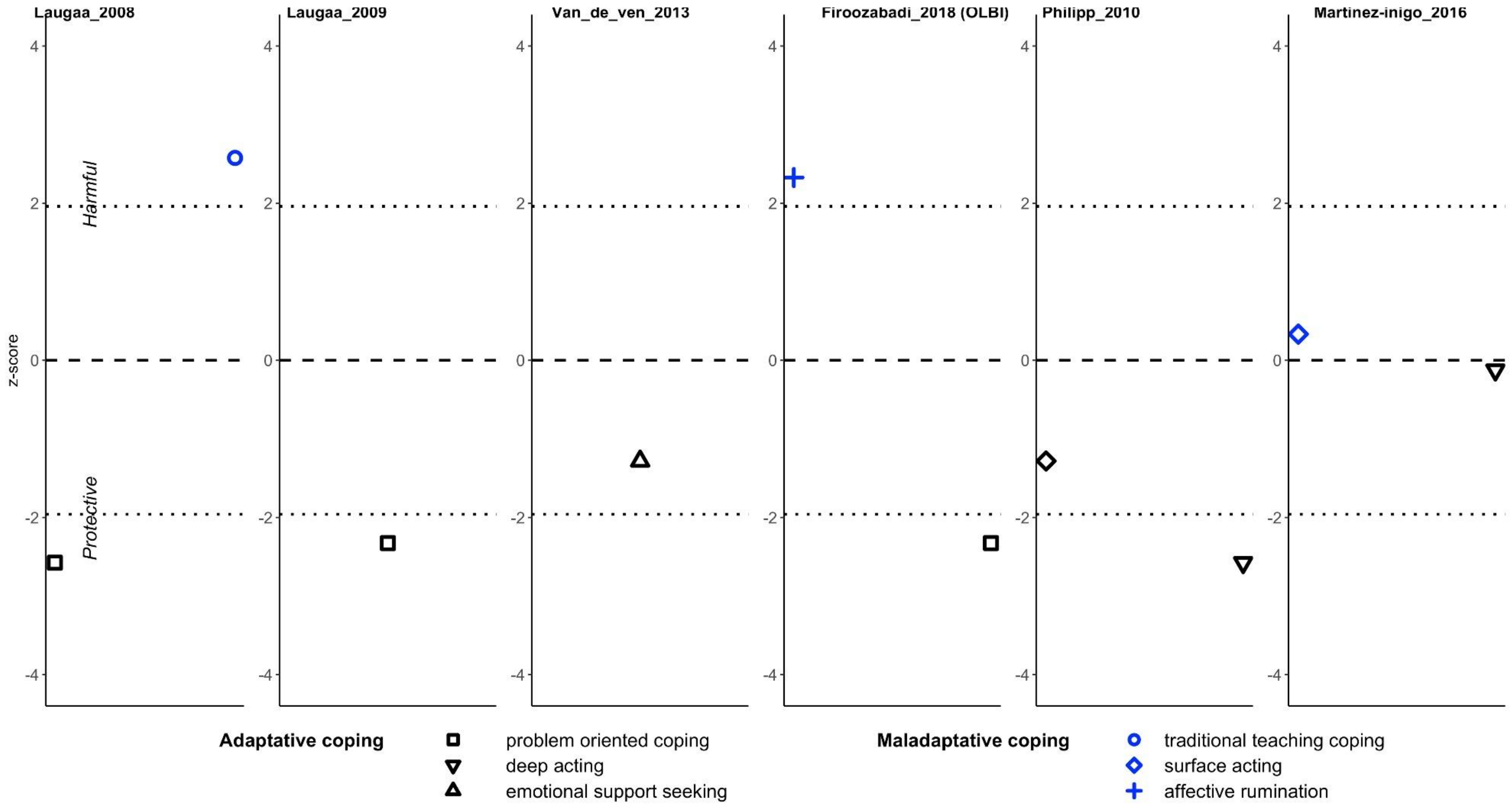

Laugaa_2008
Laugaa_2009
Van_de_ven_2013
Firoozabadi_2018 (OLBI)
Philipp_2010
Martinez-inigo_2016
z-score
Harmful
Protective
4
2
0
-2
-4
Adaptative coping
problem oriented coping
deep acting
emotional support seeking
Maladaptative coping
traditional teaching coping
surface acting
affective rumination

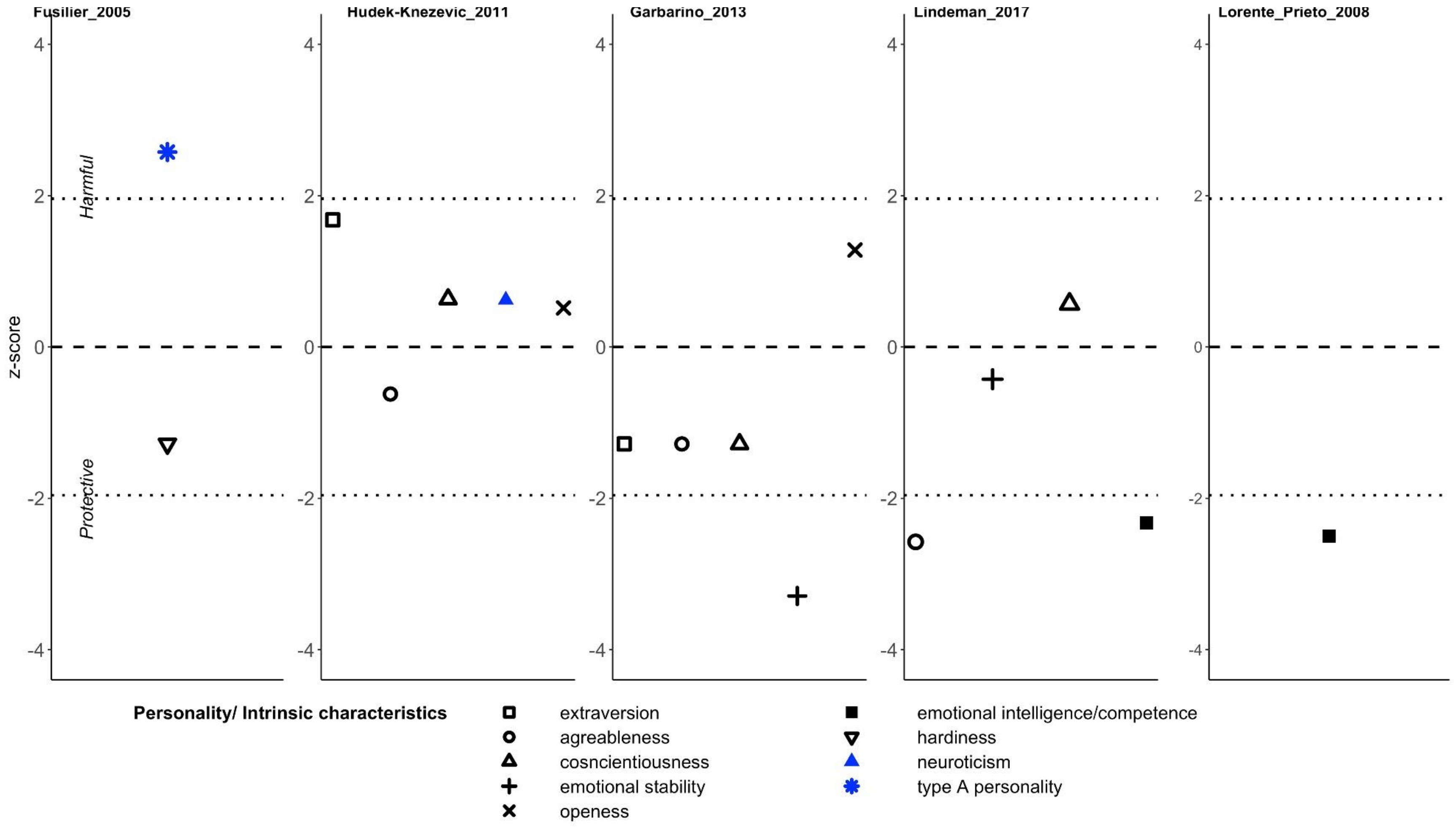

Fusilier_2005
Hudek-Knezevic_2011
Garbarino_2013
Lindeman_2017
Lorente_Prieto_2008
4
2
0
-2
-4
z-score
Harmful
Protective
Personality/ Intrinsic characteristics
extraversion
agreableness
cosncientiousness
emotional stability
openess
emotional intelligence/competence
hardiness
neuroticism
type A personality

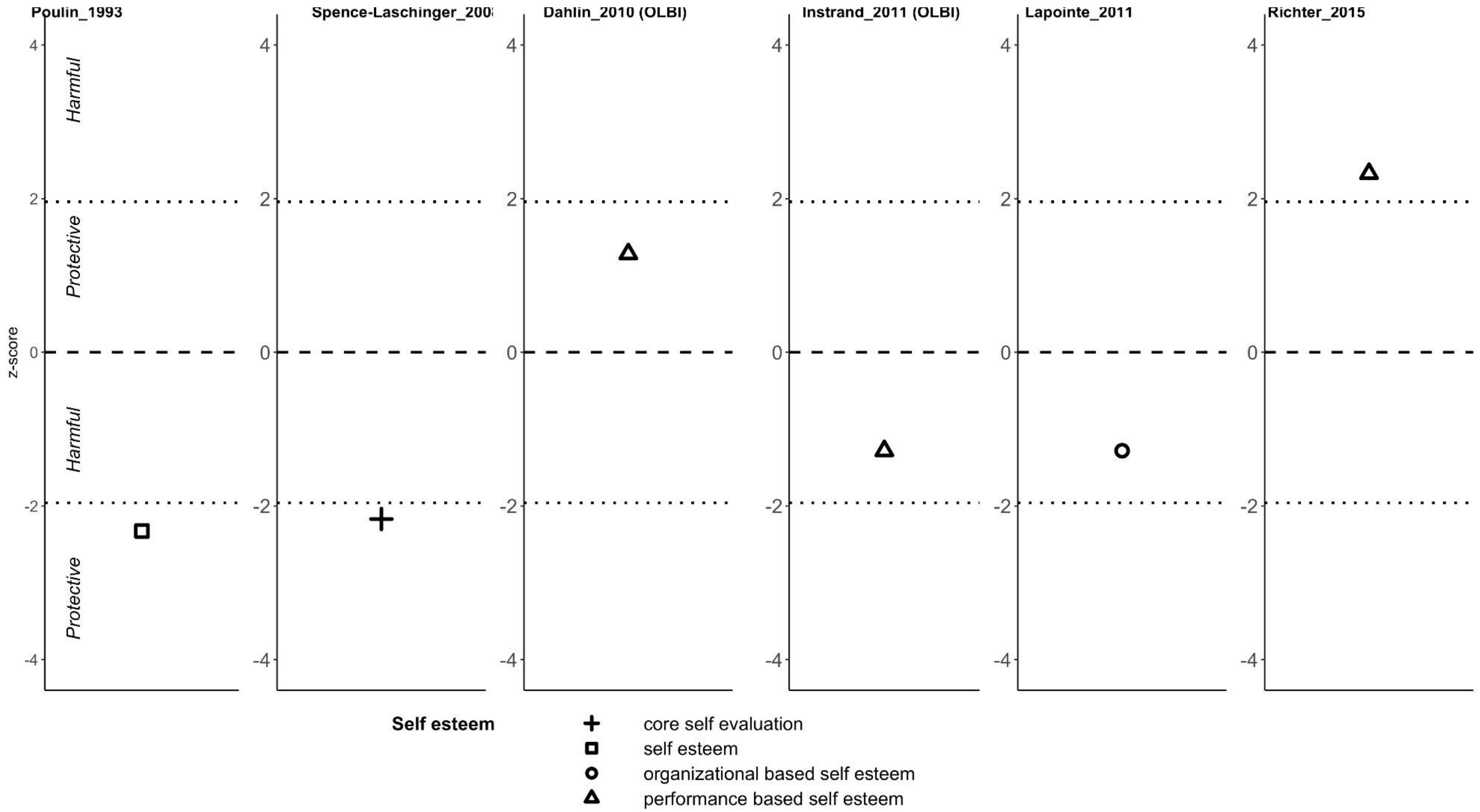
Poulin_1993
Spence-Laschinger_2008
Dahlin_2010 (OLBI)
Instrand_2011 (OLBI)
Lapointe_2011
Richter_2015
z-score
Harmful
Protective
Harmful
Protective
Self esteem
core self evaluation
self esteem
organizational based self esteem
performance based self esteem

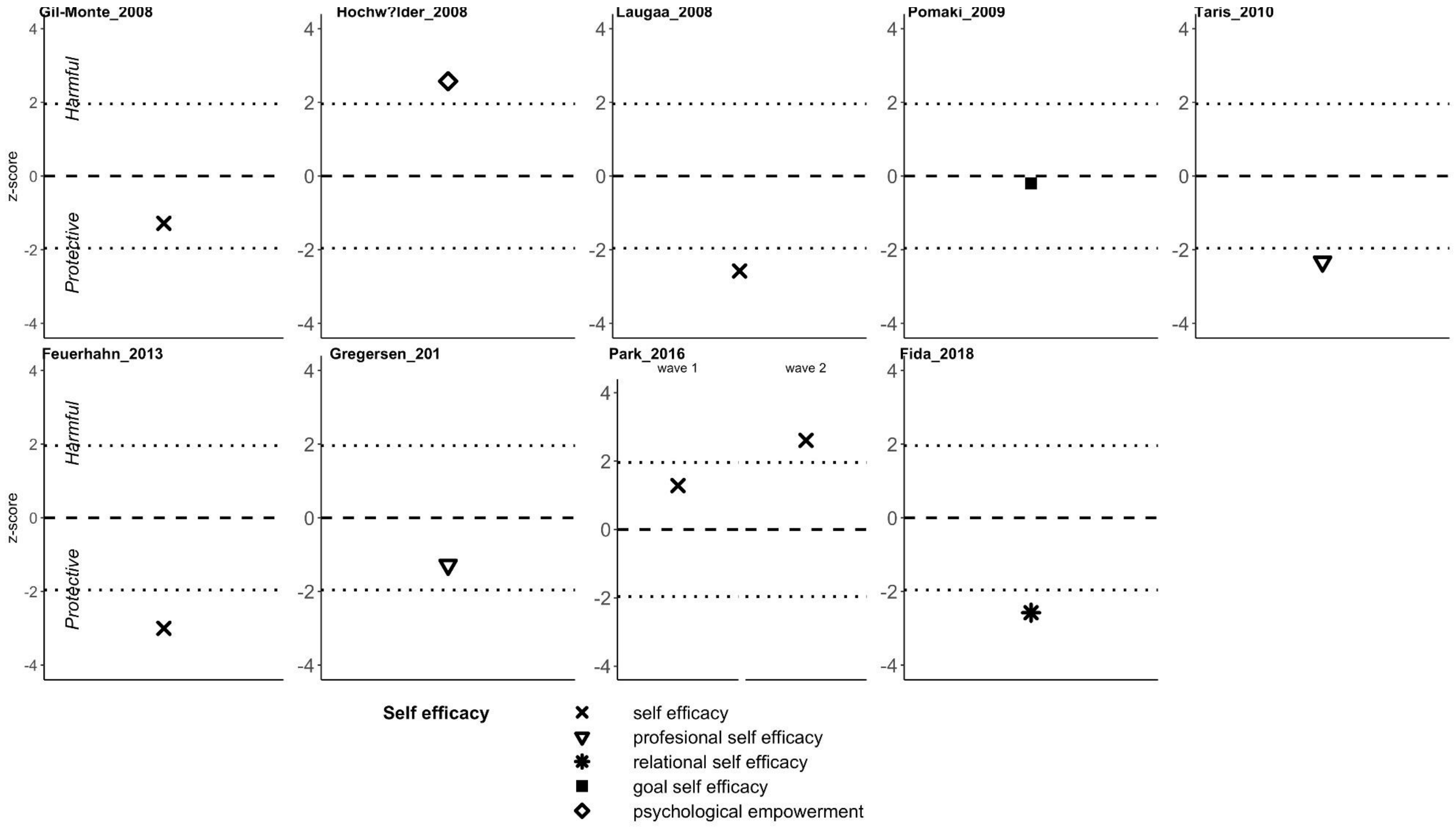
Gil-Monte_2008
Hochw?lder_2008
Laugaa_2008
Pomaki_2009
Taris_2010
Feuerhahn_2013
Gregersen_201
Park_2016
wave 1
wave 2
Fida_2018
z-score
Harmful
Protective
4
2
0
-2
-4
Self efficacy
self efficacy
profesional self efficacy
relational self efficacy
goal self efficacy
psychological empowerment

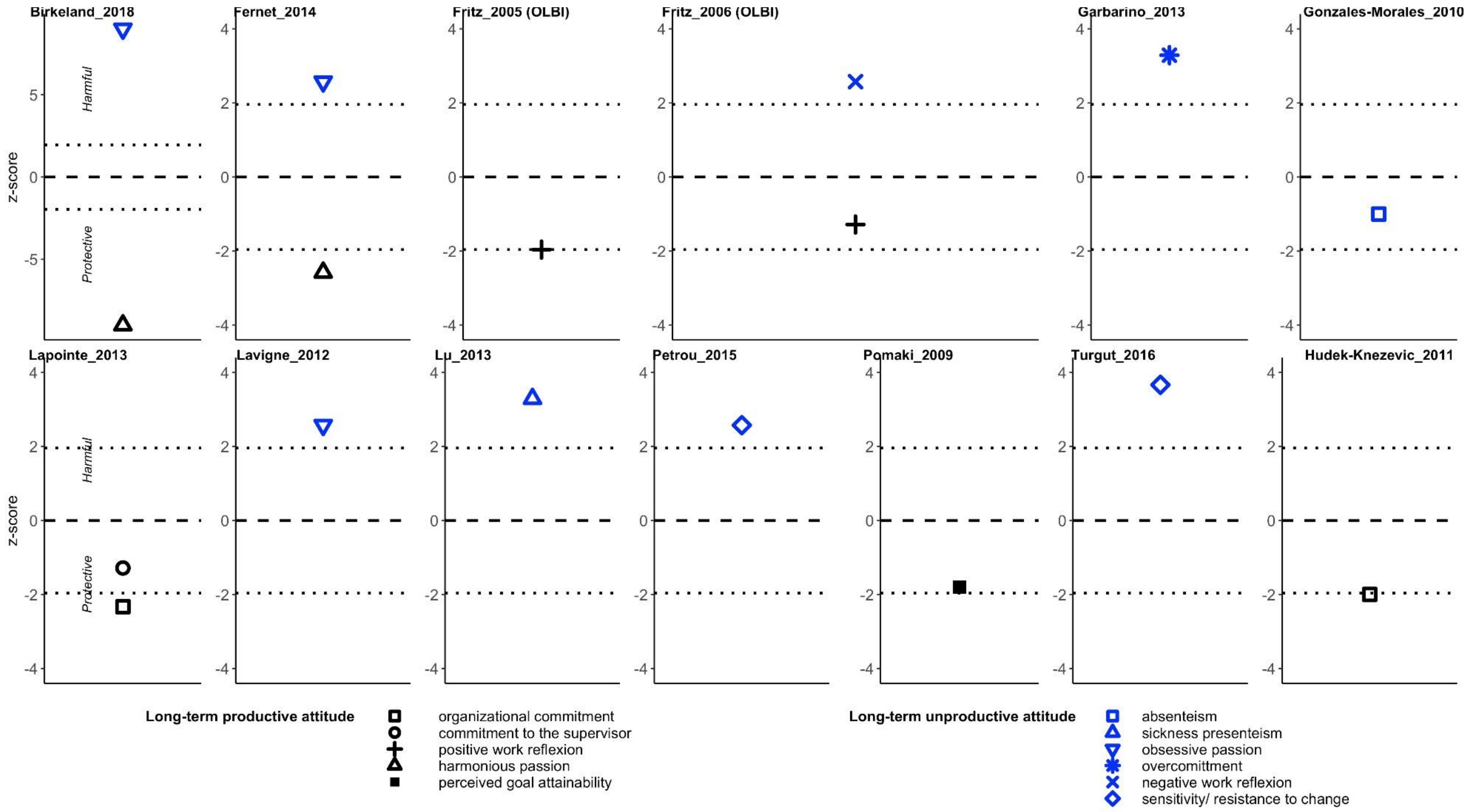
Birkeland_2018
Fernet_2014
Fritz_2005 (OLBI)
Fritz_2006 (OLBI)
Garbarino_2013
Gonzales-Morales_2010
Lapointe_2013
Lavigne_2012
Lu_2013
Petrou_2015
Pomaki_2009
Turgut_2016
Hudek-Knezevic_2011
z-score
Harmful
Protective
Long-term productive attitude
organizational commitment
commitment to the supervisor
positive work reflexion
harmonious passion
perceived goal attainability
Long-term unproductive attitude
absenteism
sickness presenteism
obsessive passion
overcomittment
negative work reflexion
sensitivity/ resistance to change

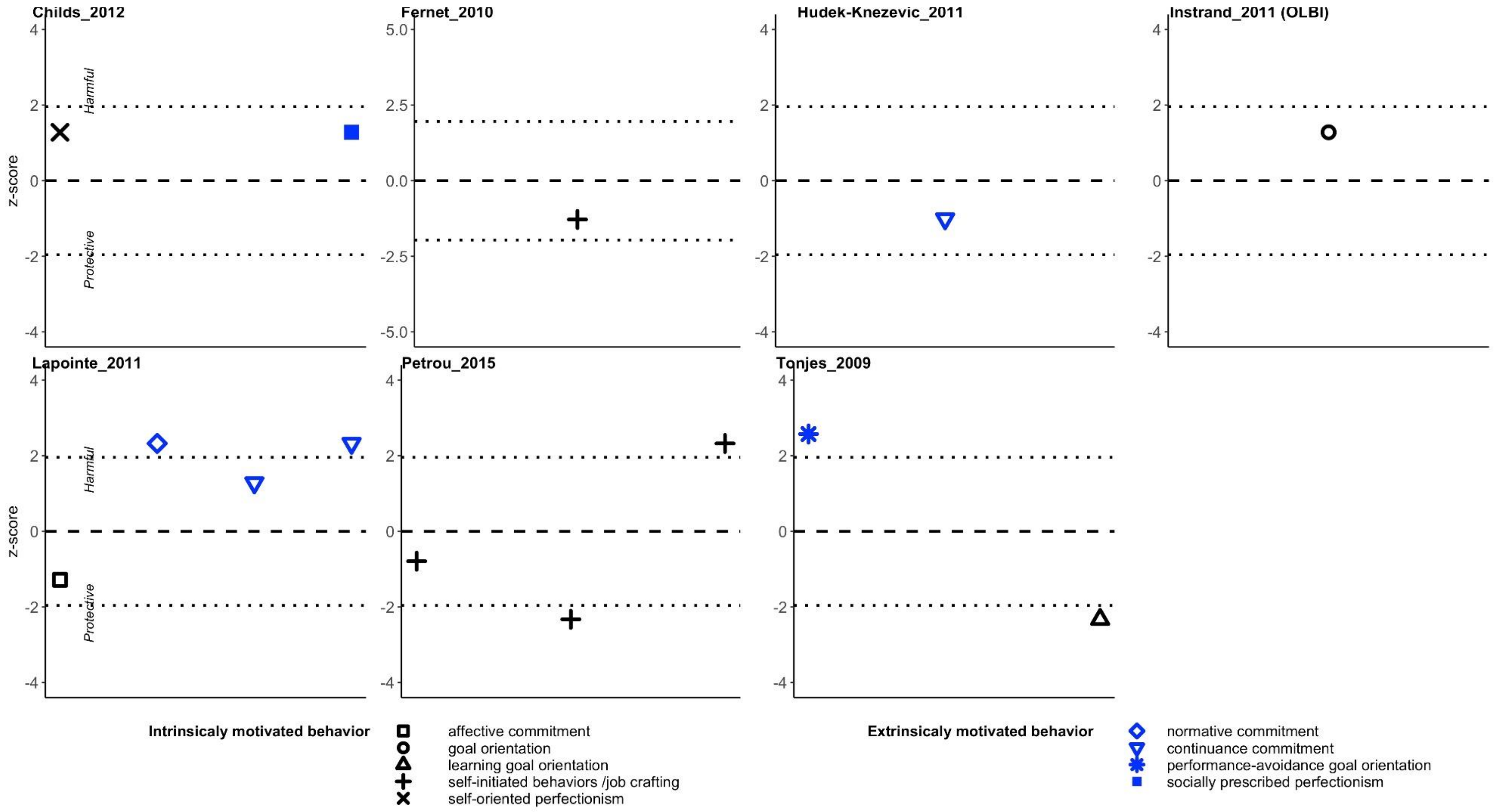

Childs_2012
Fernet_2010
Hudek-Knezevic_2011
Instrand_2011 (OLBI)
Lapointe_2011
Petrou_2015
Tonjes_2009
z-score
Harmful
Protective
Intrinsicaly motivated behavior
affective commitment
goal orientation
learning goal orientation
self-initiated behaviors /job crafting
self-oriented perfectionism
Extrinsicaly motivated behavior
normative commitment
continuance commitment
performance-avoidance goal orientation
socially prescribed perfectionism

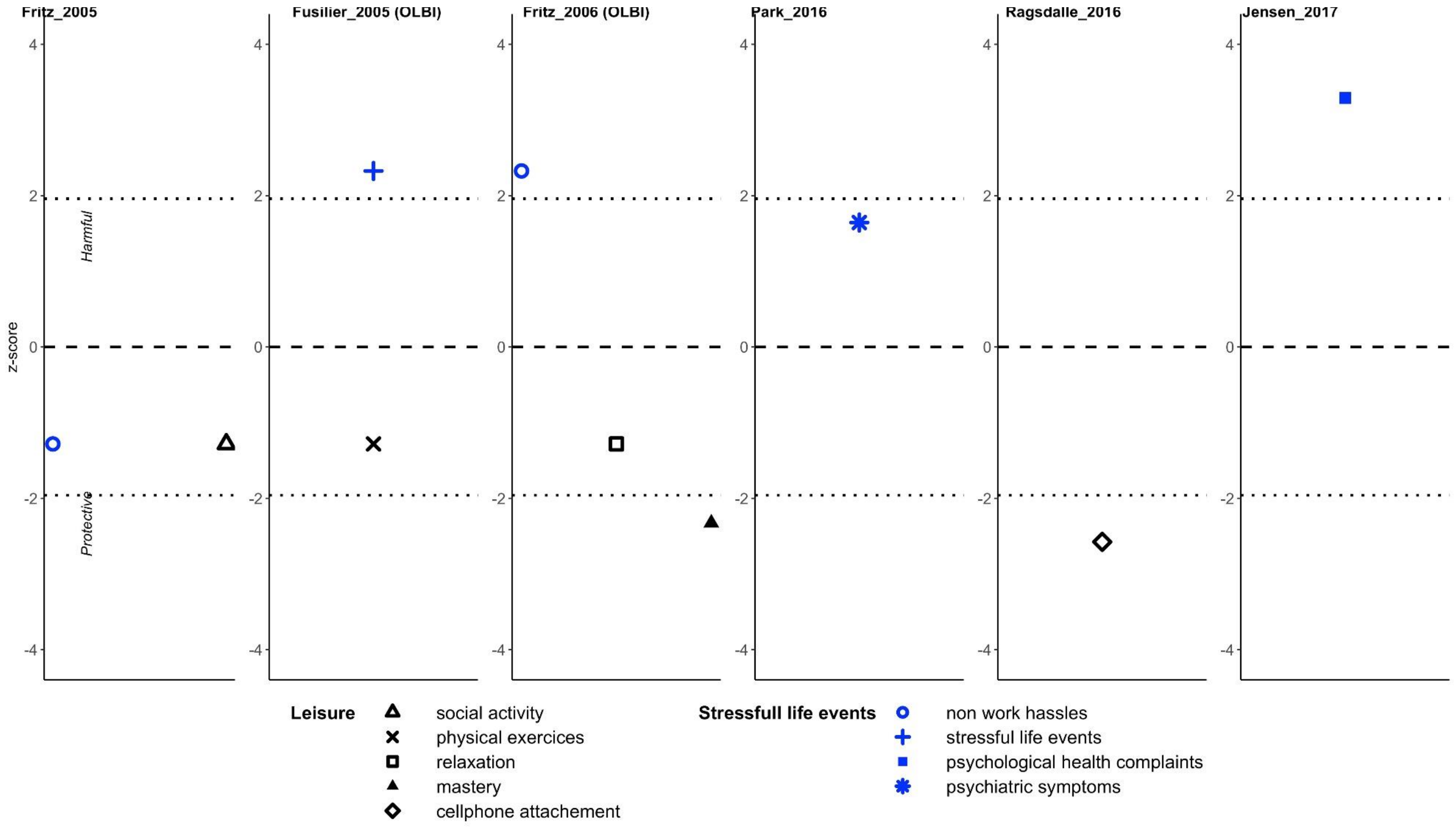

Fritz_2005
Fusilier_2005 (OLBI)
Fritz_2006 (OLBI)
Park_2016
Ragsdalle_2016
Jensen_2017
z-score
Harmful
Protective
4
2
0
-2
-4
Leisure
social activity
physical exercices
relaxation
mastery
cellphone attachement
Stressfull life events
non work hassles
stressful life events
psychological health complaints
psychiatric symptoms

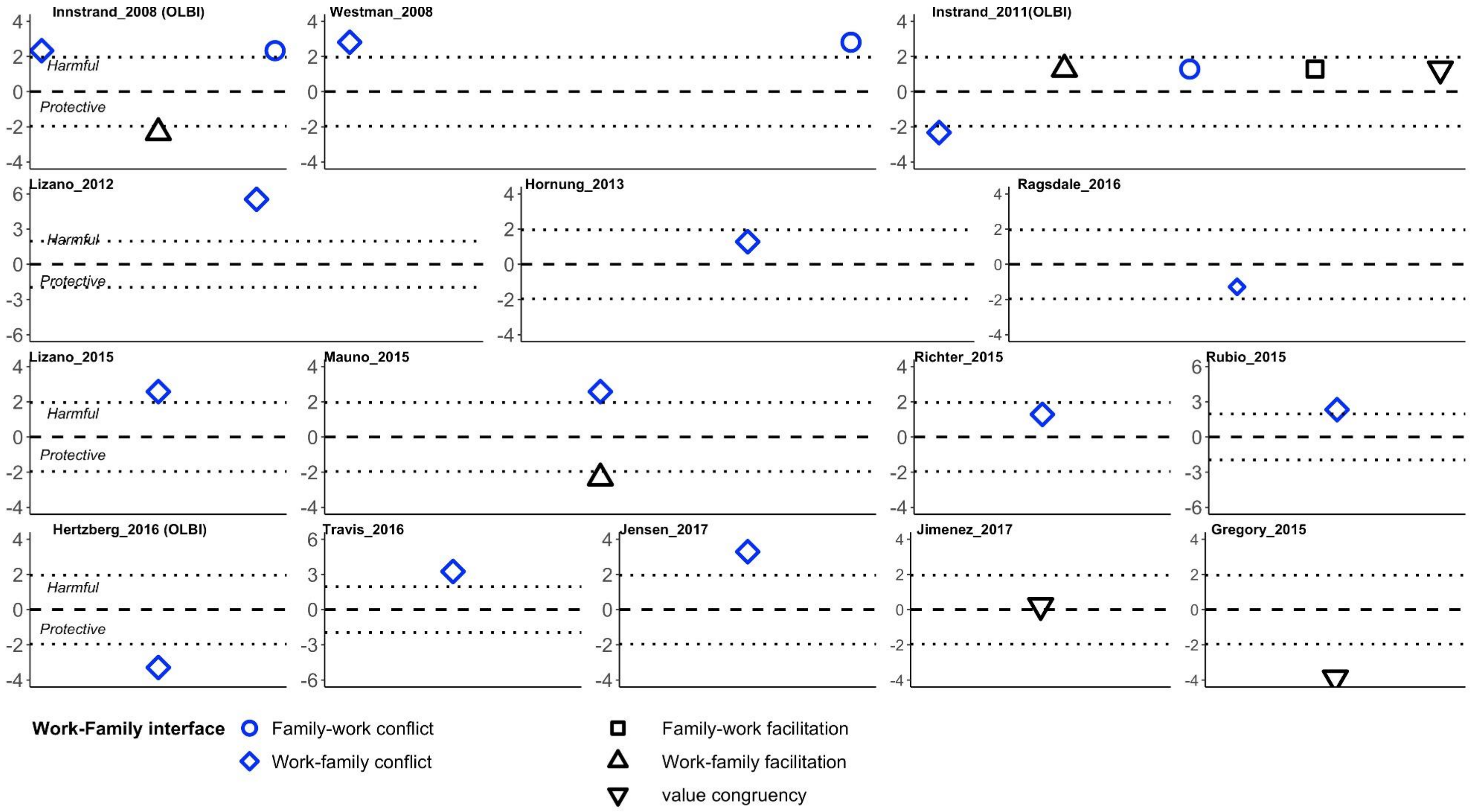
Innstrand_2008 (OLBI)
Westman_2008
Instrand_2011(OLBI)
Lizano_2012
Hornung_2013
Ragsdale_2016
Lizano_2015
Mauno_2015
Richter_2015
Rubio_2015
Hertzberg_2016 (OLBI)
Travis_2016
Jensen_2017
Jimenez_2017
Gregory_2015
Harmful
Protective
Work-Family interface
Family-work conflict
Work-family conflict
Family-work facilitation
Work-family facilitation
value congruency

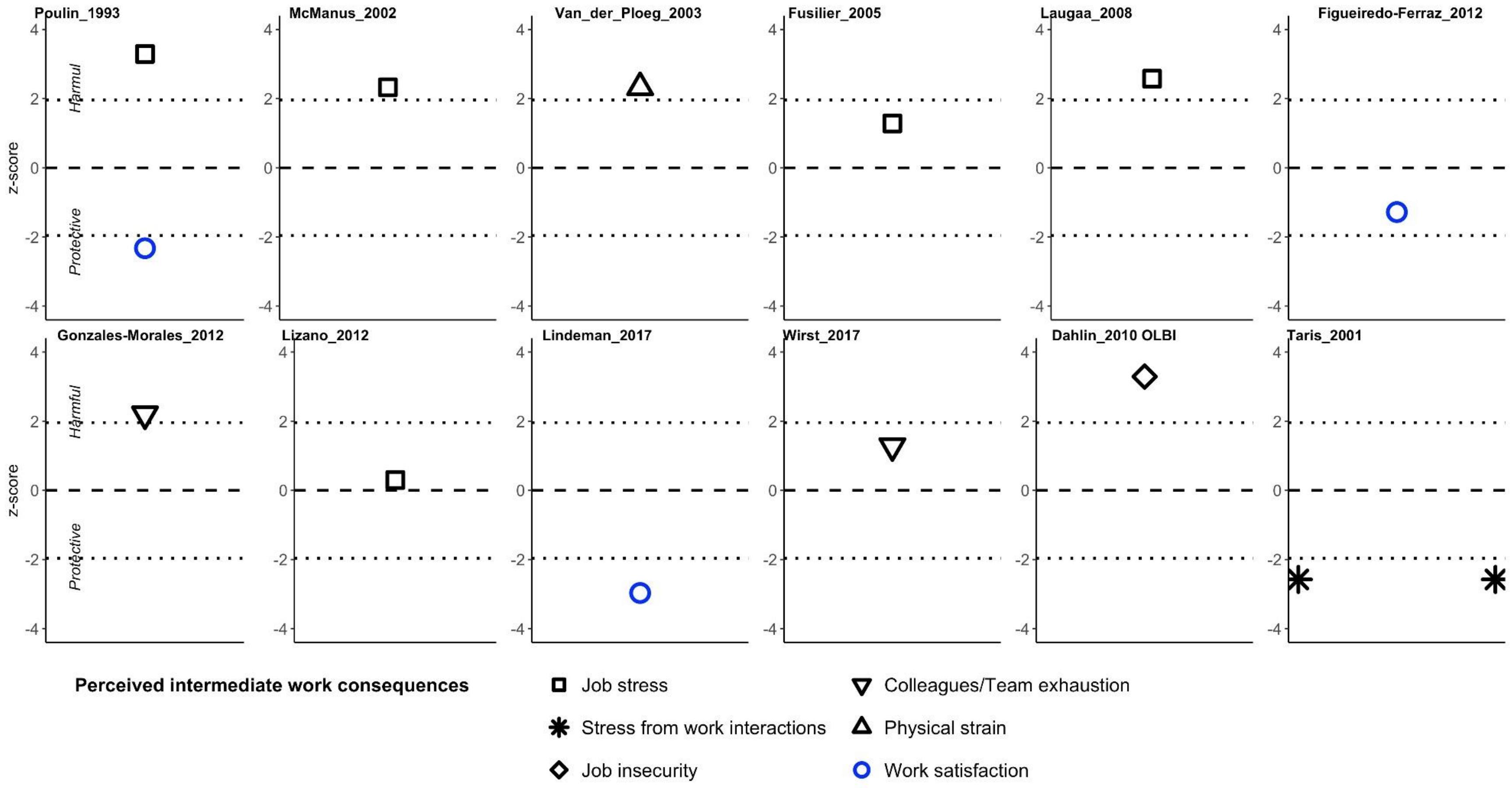
Poulin_1993
McManus_2002
Van_der_Ploeg_2003
Fusilier_2005
Laugaa_2008
Figueiredo-Ferraz_2012
Gonzales-Morales_2012
Lizano_2012
Lindeman_2017
Wirst_2017
Dahlin_2010 OLBI
Taris_2001
z-score
Harmul
Harmful
Protective
4
2
0
-2
-4
Perceived intermediate work consequences
Job stress
Stress from work interactions
Job insecurity
Colleagues/Team exhaustion
Physical strain
Work satisfaction
